# Analysis of public RNA-sequencing data reveals biological consequences of genetic heterogeneity in cell line populations

**DOI:** 10.1038/s41598-018-29506-3

**Published:** 2018-07-25

**Authors:** Erik Fasterius, Cristina Al-Khalili Szigyarto

**Affiliations:** 1 School of Chemistry, Biotechnology and Health, KTH-Royal Institute of Technology, Stockholm, Sweden; 2 Science for Life Laboratory, KTH–Royal Institute of Technology, Solna, Sweden

## Abstract

Meta-analysis of datasets available in public repositories are used to gather and summarise experiments performed across laboratories, as well as to explore consistency of scientific findings. As data quality and biological equivalency across samples may obscure such analyses and consequently their conclusions, we investigated the comparability of 85 public RNA-seq cell line datasets. Thousands of pairwise comparisons of single nucleotide variants in 139 samples revealed variable genetic heterogeneity of the eight cell line populations analysed as well as variable data quality. The H9 and HCT116 cell lines were found to be remarkably stable across laboratories (with median concordances of 99.2% and 98.5%, respectively), in contrast to the highly variable HeLa cells (89.3%). We show that the genetic heterogeneity encountered greatly affects gene expression between same-cell comparisons, highlighting the importance of interrogating the biological equivalency of samples when comparing experimental datasets. Both the number of differentially expressed genes and the expression levels negatively correlate with the genetic heterogeneity. Finally, we demonstrate how comparing genetically heterogeneous datasets affect gene expression analyses and that high dissimilarity between same-cell datasets alters the expression of more than 300 cancer-related genes, which are often the focus of studies using cell lines.

## Introduction

As the number of gene expression experiments continue to increase, so do the availability of datasets in publicly available data repositories, such as the Gene Expression Omnibus (GEO)^1^. Comparisons of in-house data and public datasets enable researchers to contrast their results to existing information in a biologically meaningful way, while meta-analyses of public datasets can yield biologically and technically relevant information that the individually analysed constituent datasets cannot^2^. The scientific context of different studies vary greatly, but the chosen context does not, however, preclude the possibility of subsequently investigating other scientific questions, making re-analysis of previously published data an important venture to achieve novel insights^3^. Indeed, some of the earliest “Big Data” articles’ citations have been mainly attributed to novel results from re-analyses of the data rather than the original conclusions themselves^4^. Re-analyses are also an efficient use of scientific resources, as new conclusions can be drawn without needing to perform new and costly sequencing experiments. Integration of different data types (*e*.*g*. transcriptomics and genomics) is also becoming more relevant, as they can reveal a more complete biological picture^5^. Integration of data collections are especially important, since they can yield insights into biological questions difficult to assess by direct experiment^6^. Before any such project can start, however, investigations need to be made to ensure that included datasets are comparable. There are several reasons why a dataset might be disqualified from inclusion, such as low data quality or non-equivalent biological sources^2^.

Cell lines are commonly used as *in vitro* models for cancer and drug testing, but a considerable problem is that of cell line *authenticity*: whether or not the cells used are biologically equivalent to their original source^7^. While cell lines are excellent, easy-to-use sources of unlimited experimental materials that side-steps the ethical and practical issues related to using human samples, authenticity remains a major concern. Unauthentic cells can arise because of several reasons, such as cross-contamination by another cell line, mislabelling at the lab or genetic drift due to long-term culturing. It has been shown that between 15% and 20% of all cell lines are misidentified or contaminated^8^. Mycoplasma infections also affect the cells, but can be avoided by performing routine tests and using proper culturing techniques^9^. The HeLa cell line is among the most frequent sources of cross-contamination due to its ubiquitous use in laboratories across the globe. Not only do problems arise during culturing and experimentation on cell lines, but it is now apparent that many have become contaminated at the time of their creation^10^.

Analysing short tandem repeats (STRs) in the cell line of interest and comparing the results to a database is the *de facto* standard recommended by the American Type Culture Collection (ATCC), but analysis of single nucleotide variants (SNVs) is also becoming increasingly used^11,12^. There are, however, problems with using STR profiling as the basis for cell line authenticity, such as microsatellite instability and genetic heterogeneity^13,14^. Researchers have recently shown that a batch of the MCF7 cell line possessed genetic heterogeneity that affected its phenotype, while still yielding a perfect STR match to the ATCC reference^15^.

As RNA sequencing (RNA-seq) has been shown to be highly robust across both platforms, laboratories and experimental designs^16^, we previously developed a method to analyse RNA-seq for cell line authentication^17^. The method uses the vast amounts of sequence information available from RNA-seq experiments to compare variants with the *Catalogue of Somatic Mutations in Cancer* (COSMIC) database on a larger scale than conventional STR or SNV profiling does^18^. While SNVs are traditionally analysed with genomic methods, it has previously been shown that 40% to 80% of variants discovered using whole genome sequencing are also found by RNA-seq^19^. There are numerous studies empirically proving that RNA variant analysis can yield novel biological insights^20–22^. This highlights the ability of RNA-seq to also be used for variant analysis (in addition to standard gene expression studies), greatly increasing its utility. One of the strengths of the method is its capacity for re-analysis of existing sequencing data, allowing it to investigate any publicly available RNA-seq datasets as well as novel data. Another advantage is its potential to analyse variants across the entire transcriptome, rather than a preset number of STRs or SNVs, thus greatly increasing its statistical power. In addition to filling the need for new and robust methods for cell line authentication highlighted by Freedman *et al*.^23^, the method both authenticates cell lines to a high degree of certainty as well as provides detailed information about deviations from known variants in the cells. We also demonstrated that our method could potentially be used for transcriptome-wide authentications, taking the totality of overlapping SNVs in each dataset into account. This would represent an improvement over *e*.*g*. STR panels, as the global analysis of individual mutations is important for many diseases^24^.

Herein, we present one of the largest studies of genetic heterogeneity and comparability in public datasets to date using the previously published method, performing several thousands of pairwise comparisons of 139 samples across 85 RNA-seq datasets and eight cell lines in the GEO. We characterise the varying degrees of genetic heterogeneity present in the different cell lines and confirm that this heterogeneity has an effect on gene expression and cellular functions, reveal that H9 and HCT116 are remarkably stable while HeLa possesses a high degree of variation, and highlight a mislabelled MCF7 dataset. We thus demonstrate the importance of checking that public data used for new analyses are based on biologically equivalent sources and provide a general workflow demonstrating how this can be achieved.

## Results

### Selection of GEO datasets and experimental design

In order to investigate the comparability of cell line data, the GEO was queried and filtered to only include cell lines with at least ten RNA-seq datasets to ensure statistical power and a collection of samples representative for the cell line populations currently used in research. The cell lines were additionally required to be available in the COSMIC database. Due to the nature with which metadata is stored in the GEO this process had to be performed in several steps, the last of which required manual curation. Datasets were chosen to yield at least 50 × 10^6^ reads on average across one or more samples in order to control for biases in sequencing depth, contain a mix of single- and paired-end data and to be of “wild type” origin; samples that contained gene knockouts, genetic transformations, treatments or other perturbations (according to the metadata) were ignored, as such samples could confound the underlying biology being compared. A total of 139 samples from 85 different datasets and eight cell lines were selected to be analysed (Table 1): A549 (lung carcinoma), H9 (lymphoma), HCT116 (colon carcinoma), HeLa (cervical adenocarcinoma), K562 (leukaemia), MCF7 (breast adenocarcinoma), MDAMB231 (breast adenocarcinoma) and U2OS (osteosarcoma).

**Table 1:** Summary of the available and analysed cell line datasets. High quality samples from almost half of all the available datasets in the GEO were analysed, covering on average 68 million reads per dataset.

Cell line	Total datasets	Total samples	Analysed datasets	Analysed samples	Average reads per dataset
A549	13	79	7	12	48 × 10^6^
H9	13	833	10	19	61 × 10^6^
HCT116	26	272	15	20	66 × 10^6^
HeLa	66	530	18	29	85 × 10^6^
K562	17	126	8	12	75 × 10^6^
MCF7	33	301	12	19	77 × 10^6^
MDAMB231	11	153	8	17	64 × 10^6^
U2OS	11	151	7	11	65 × 10^6^
Total	190	2,445	85	139	68 × 10^6^

### Comparisons of the generated SNV profiles with COSMIC data fail to evaluate all datasets

**Figure 1:**
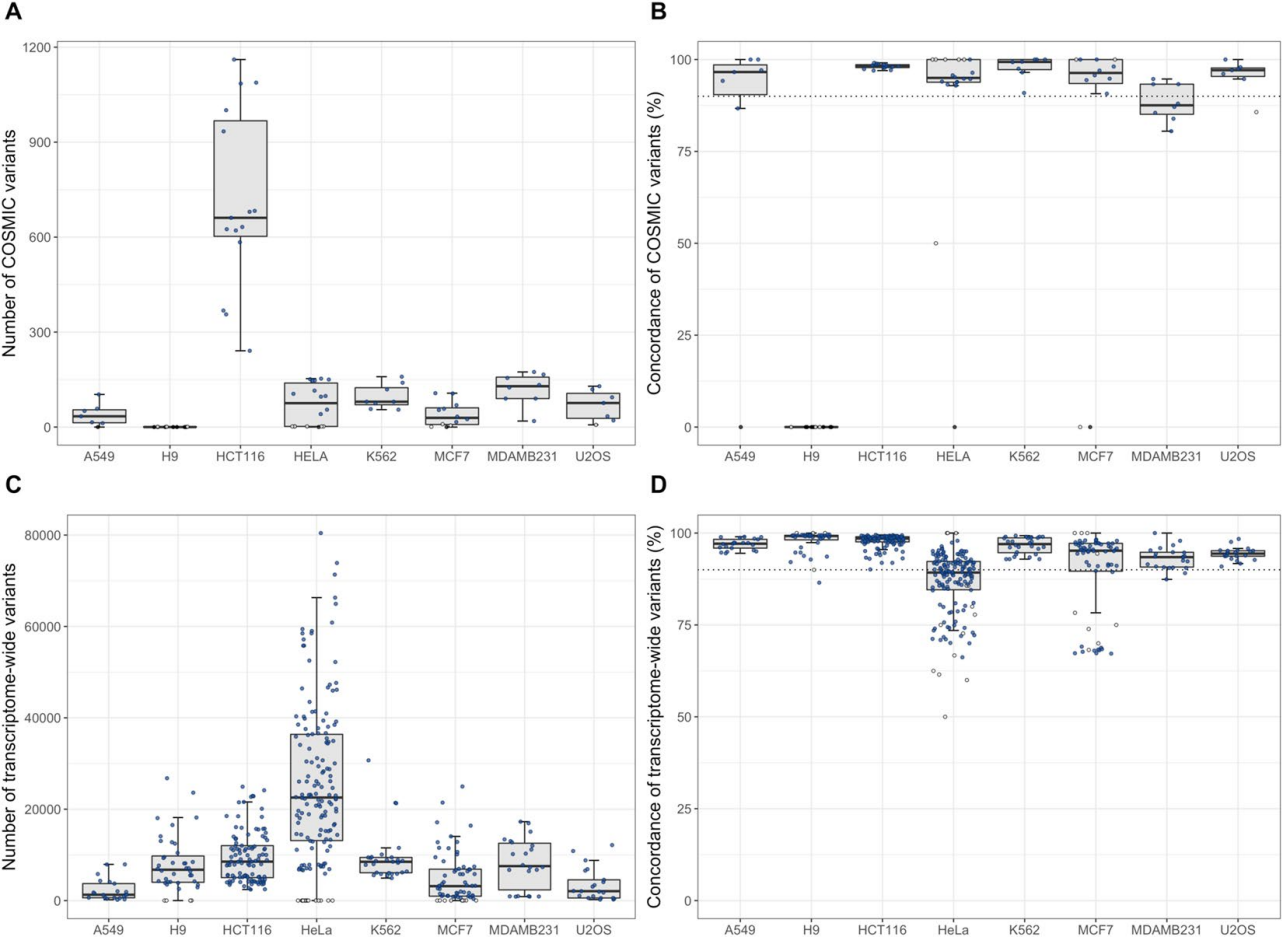
Overview of the comparisons between the identified variants in the GEO datasets. (**A**) The number of variants overlapping with COSMIC variants, (**B**) the concordance of COSMIC overlaps, (**C**) the number of overlapping variants for each pairwise transcriptome comparison, and (**D**) the concordance of each pairwise transcriptome comparison. Empty circles in the COSMIC boxplots represent dataset with less than 10 COSMIC variant overlaps, while in the transcriptome comparisons represent a pairwise comparison with less than 50 overlapping variants. Black circles in the COSMIC boxplots are dataset with zero overlapping COSMIC variants. Other values are represented with blue circles.

The selected datasets were analysed using the cell line authentication method previously presented, which includes the best-practice RNA-seq variant calling pipeline from GATK^17^. The method compares all statistically significant variants passing several quality thresholds found in a given RNA-seq dataset generated from the analysis of a specific cell line with those found in the COSMIC database for the same cell line, yielding a high throughput sequencing counterpart to existing authentication methods. All of the examined cell lines have a comparable number of SNVs listed in the COSMIC database (between 400 and 600) with the exception of HCT116, which has 2,777 in total (Table 2). We define *overlap* as the number of variants that are present in both samples for any given pairwise comparison (*i*.*e*. the RNA-seq datasets and the COSMIC database); between tens and hundreds of the COSMIC SNVs overlap with those found in the analysed datasets (Fig. 1A). Four of the cell lines (A549, H9, HeLa and MCF7) have at least one dataset without any overlapping variants whatsoever (SFigure 1). The H9 cell line represents a clear deviation as only a single or none of the annotated COSMIC SNVs were found in its datasets.

**Table 2:** Comparison of SNVs identified in cell lines using RNA-seq data and annotated SNVs in the COSMIC database. COSMIC variants are found for all the cell lines, with the exception of H9. The median concordances are above 95% for all cells except MDAMB231, with CVs going as high as 47%.

Cell line	Total COSMIC SNVs	Median overlapping SNVs	Median concordance	Coefficient of variation: concordance
A549	433	34	96.9%	44.4%
H9	527	0	—	—
HCT116	2,777	661	98.3%	0.6%
HeLa	466	76	95.2%	28.0%
K562	554	80	99.4%	3.2%
MCF7	524	29	96.4%	46.8%
MDAMB231	607	129	87.6%	5.8%
U2OS	418	76	97.1%	4.9%

In order to measure the similarity between the RNA-seq variants and the COSMIC profiles, the *concordance* is defined as the proportion of matching SNVs (*i*.*e*. SNVs with the same genotype in both samples) relative to the overlap (*i*.*e*. SNV matches ÷ total SNV overlaps). Six out of seven cell lines (discounting H9) have a median concordance above 90% (a previously employed threshold for cell authenticity)^12^, while MDAMB231 has 87.6% (Fig. 1B). Four cell lines (A549, HeLa, MDAMB231 and U2OS) have at least one dataset with an individual concordance below 90% (SFigure 1). Concordance CVs range from 0.6% to 46.8%, indicating an inherent variability in COSMIC SNV comparisons (Table 2).

The number of variants differing from the COSMIC database does not, however, necessarily reflect the biological effect of the mismatched SNVs. The annotated SNV impacts (HIGH, MODERATE, LOW or MODIFIER, where HIGH impact variants have negative effect on protein function) more clearly reflect the biology of the SNVs. The distribution of matched and mismatched SNV impacts is comparable to previous cell line data (SFigure 2). However, a large proportion of the mismatched COSMIC SNVs are homozygous variants (83.2%; SFigure 3), indicating that there is a difference between the COSMIC database and the RNA-based SNVs found in the datasets.

These results indicate that the COSMIC-based cell authentication method is useful, but that its coverage of annotated SNVs is insufficient for a thorough investigation of dataset comparability.

### Transcriptome-wide variant analysis reveal cell line heterogeneity

By examining transcriptome-wide SNVs instead of only considering annotated COSMIC variants the analysis will gain statistical power and create opportunities for functional assessment, in addition to provide opportunities for completing and adding to the COSMIC data. Such an analysis can capture biological variation in entire cell line populations and better highlight problems with data quality. We thus performed several thousands of transcriptome-wide, pairwise dataset comparisons using the totality of the high-quality SNVs from the variant calling pipeline. Boxplots of the total number of overlapping variants and the concordance for each comparison is shown in Fig. 1C,D. As can be seen in Table 3, there are thousands of transcriptome-wide variants found in the different datasets, ranging from over 100,000 overlapping variants for HeLa to 1,280 for A549. The median overlapping variants of comparisons between different cell lines is 3,569, with a median concordance of 65.2%, yielding an estimate of the baseline similarity between two arbitrary cell lines. A concordance of approximately 65% is thus a strong indication that the two cell lines being compared are not equivalent.

**Table 3:** Transcriptome-wide analysis of SNV identified per cell line in analysed data sets. Tens of thousands of variants are found for each cell line, with several thousands of overlaps across the different datasets. The concordances are highly stable across all cell lines: most CVs are below 4%.

Cell line	Median variants	Median overlapping SNVs	Median concordance	Coefficient of variation: concordance	Median score
A549	5,106	1,280	97.1%	1.5%	96.6
H9	18,903	6,750	99.2%	3.8%	99.1
HCT116	26,273	8,525	98.5%	1.9%	98.5
HeLa	136,724	22,554	89.3%	10.2%	89.0
K562	12,983	8,490	97.0%	2.3%	97.0
MCF7	10,887	3,162	95.2%	12.7%	95.0
MDAMB231	15,422	7,547	93.5%	3.2%	93.1
U2OS	12,505	2,060	94.4%	1.9%	94.0

The transcriptome-wide concordance of the HeLa cell line is the only one to drop below a median of 90%; MDAMB231, on the other hand, reaches 93.5% (SFigure 4). The H9 cell line possesses a median overlap of 6,750 and concordance above 99%. HCT116 show similar results, with 8,525 SNVs and a median concordance of 98.5%. The coefficients of variation are below 4% in six out of eight cases, with HeLa and MCF7 remaining the highest (slightly above 10%). The high concordances and low variability of the H9 and HCT116 cell lines could indicate a higher genomic stability, compared to the other analysed cell lines.

It is also possible to evaluate variants in specific genes of interest by looking at the whole transcriptome. The HCT116 cell line, for example, should have a heterozygous *C/T* genotype at a site in the KRAS gene, known as the G13D mutation. By looking at this site in all the investigated datasets, we can confirm this known mutation in the HCT116 samples (STable 1). Such an analysis is possible for any known mutation and constitutes an important part of evaluating biological equivalency not only on a transcriptome-wide level, but also on specific gene products.

There are three datasets from the H9, HeLa and MCF7 cell lines that have a low number of identified SNVs in total (13, 68 and 42, respectively), compared to the other transcriptome-wide datasets (SFigure 4B,D,F). The pairwise concordances of these datasets have a wide range, going from 0% up to 100% (across both different- and same-cell comparisons), most likely due to random SNV matches across a small number of variants. In order to account for such datasets, we aimed to weigh the concordances in an unfavourable way for comparisons with few variants. Comparing the transcriptome-wide variants between two samples or datasets can be thought of as a binomial experiment: each individual variant comparison is a trial where matching variants are successes. We thus define the *similarity score* as (*s* + *a*) ÷ (*n* + *a* + *b*), where *s* is the number of matching variants, *n* the number of overlapping variants, *a* = 1 and *b* = 5. The variables *a* and *b* were selected to yield a cutoff equivalent to the one used by Yu *et al*.^12^, resulting in a lower bound of 44 perfectly matching variants yielding a score of 90; this better utilises the increased statistical power from the greater number of variants in transcriptome-wide analyses and better highlights potential problems with data quality.

**Figure 2:**
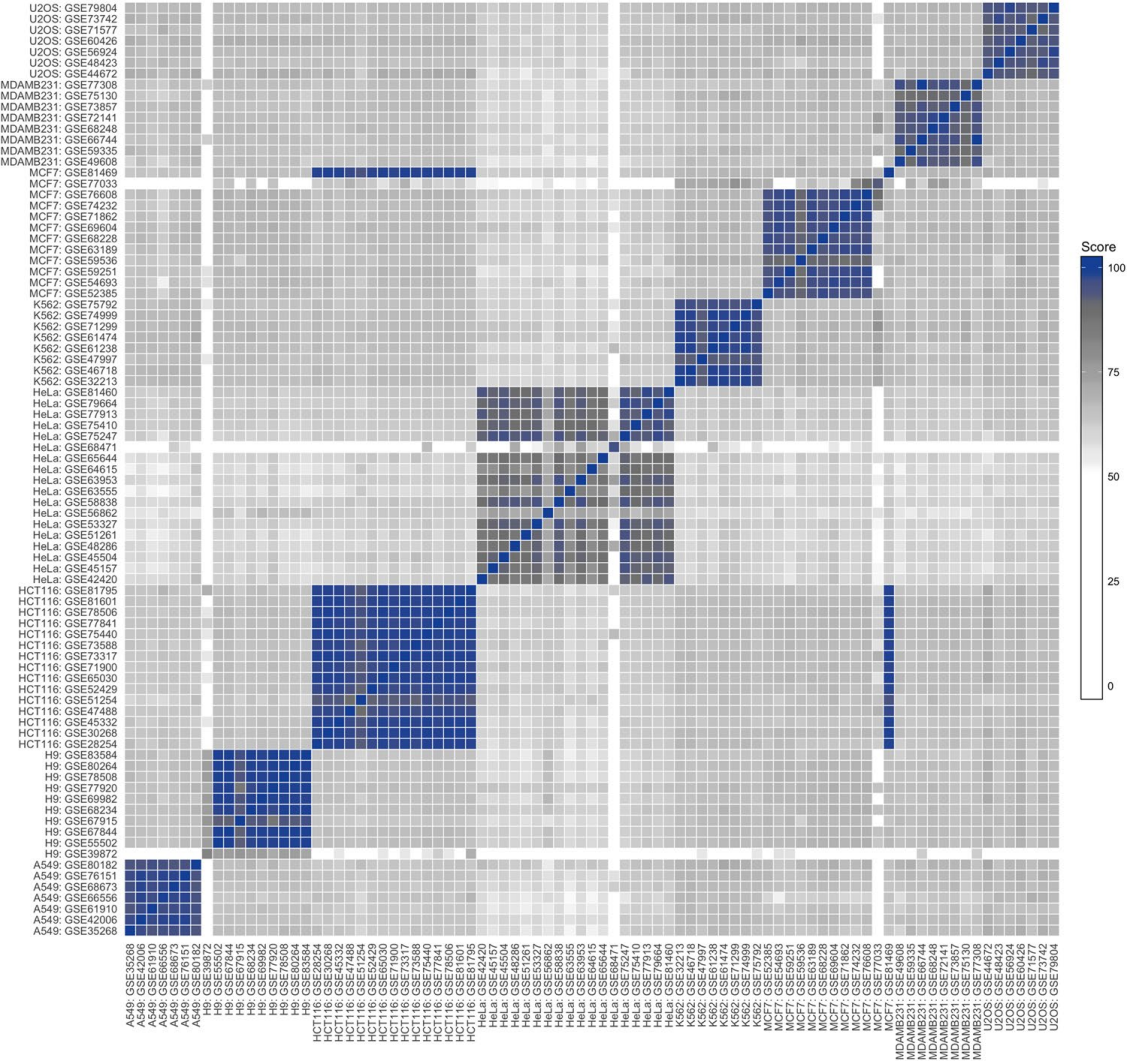
Heatmap of transcriptome-wide cell line similarity scores of pairwise comparisons of all datasets included in the study: scores below 50 are shown as pure white, with a grey colour gradient from 50 to 90 and a blue gradient up to a score of 100. The highest similarities are found for same-cell comparisons across the diagonal, with HeLa being the most genetically heterogenous. Three datasets have very few variants to compare, while a single MCF7 dataset is more similar to HCT116 than other MCF7 datasets.

In order to visualise the large-scale analysis of all the datasets investigated herein, a heatmap of the similarity score for each of the thousands of pairwise comparisons performed is shown in Fig. 2 (see SFigures 5 to 12 for individual cell line heatmaps). The highly similar datasets are clearly grouped according to cell line, but most same-cell groups also possess varying levels of genetic heterogeneity. The lowest same-cell similarity scores are for HeLa: most are around 90 but some go as low as 70. Strikingly, one dataset from the MCF7 cell line has poor similarity with the other MCF7 datasets (around 70) while showing excellent score with all the HCT116 datasets (above 97). While this dataset has no publication associated with it at the time of writing (making a deeper investigation difficult), it is clear that this dataset contains data from HCT116 mislabelled as MCF7. It has previously been shown that certain cell lines have a higher incidence of mislabelling depending on their country of origin^14,25^. No such bias can be seen in the datasets in the present study (STable 2).

The three datasets that showed mixed concordances are seen as mostly blank columns/rows, highlighting their deviation from the bulk of datasets and need for further investigation. The reason for the low number of variants in the GSE39872 dataset (H9) could be due to the imprecise manner in which metadata is submitted and stored in the GEO: the *molecule* and *library strategy* metadata columns (which were used as selection criteria) are listed as “total RNA” and “RNA-seq”, respectively, but details in the corresponding article reveals that this data is of small RNA-seq origin, which explains the low number of variants resulting from this dataset. The GSE68471 dataset (HeLa) is likewise listed as “total RNA”, but is in fact from low complexity histone-specific libraries from a method for sequencing 3′-ends of RNA, while the GSE77033 dataset (MCF7) uses *nanoCAGE*^26^, a method for identifying 5′-ends of transcripts and discovery of new promoters. These three datasets, in addition to the mislabelled MCF7 dataset, are not included in any subsequent analyses.

Taken together, these results demonstrate that transcriptome-wide variant analyses efficiently capture the biological variation needed to investigate genetic heterogeneity and dataset comparability, as well as its capacity to highlight problems in data quality and metadata storage.

### Genetic heterogeneity in cell line populations affect gene expression

**Figure 3:**
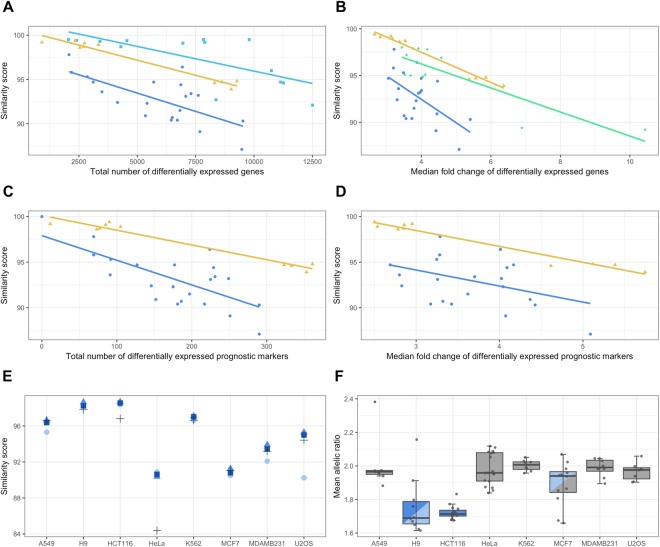
Correlation between similarity score and gene expression. Significant correlations (α = 0.01) between the similarity score versus the total number of DEGs (**A**) and the median fold change of DEGs (**B**) of each same-cell pairwise comparison: H9 is teal (correlation coefficient −0.71, number of DEGs), HCT116 yellow (−0.98 and −0.99 for the number of DEGs and median fold change, respectively), MCF7 green (−0.84, fold change only) and MDAMB231 blue (−0.68 and −0.58); (**C**,**D**), the same correlations as above, but only using differentially expressed prognostic cancer-markers: −0.98 for both DEGs and fold change in HCT116; −0.61 and −0.40 for MDAMB231; (**D**), visualisation of impact-specific similarity scores per cell line, where light blue circles signify HIGH impact, blue triangles MODERATE, marine blue squares LOW and black crosshairs MODIFIER; (**E**), ANOVA analysis with differences in chromosomal aberrations shown as boxplots of the mean allelic ratios for each cell line, where groups with differing colours are significantly different from each other.

The transcriptome-wide analyses demonstrate that the different cell lines have distinct degrees of heterogeneity: H9 and HCT116 are highly stable across datasets, while the others are more variable. These results raise the important question as to whether comparability of multiple datasets as defined in our study obscures or alters the results of common transcriptomic analyses. We thus performed differential gene expression analysis on each pairwise, same-cell dataset in order to evaluate the effect of the genetic heterogeneity present in public cell line datasets. As abundance measures (such as TPM, “transcripts per million”) are commonly available in many expression databases, this analyses was performed using the *Kallisto* software for isoform abundance estimation^27^. These abundances were subsequently summed to the gene-level by the *TXimport* R package and analysed with *edgeR* for differential expression, as it has been shown that such workflows have high accuracy^27–30^. Only datasets with at least two replicates were considered for this analysis, where genes with a two-fold expression change at a significance level of 0.01 were considered as differentially expressed genes (DEGs). The similarity score per comparison was correlated with its total number of DEGs and its median fold change at a significance level of 0.01. Figure 3A,B shows the statistically significant correlations, indicating that there is a clear negative correlation between the score and both DEG parameters, ranging from −0.58 to −0.99. As overall dataset quality is a possible confounding factor for this analysis, we also performed correlations of the number of overlapping variants for each cell line as well as the different dates at which each dataset was submitted to the GEO. Neither of these factors had a significant effect on the analysis (STable 3). Enrichment analysis of manually curated KEGG functional units was also performed for DEGs of the significantly correlated cell lines, indicating that there is a generally higher proportion of enriched categories for low similarity scores than for high ones (SFigure 13).

In order to investigate if the genetic heterogeneity present in cell line populations also affect the expression of genes known to be related to cancer we performed the same correlations as above, but for existing tissue-specific prognostic cancer markers previously published by the *Human Protein Atlas* project^31^. The H9, K562 and U2OS cells were not used for this analysis, as they do not have a corresponding HPA tissue. It is clear that the same relationship between the similarity score and DEGs still holds even for prognostic markers (Fig. 3C,D), indicating that the genetic heterogeneity can affect not only the global expression profile of the datasets, but also specific genes likely of great interest to researchers.

Another (albeit cruder) way of measuring gene expression similitude is the global correlation of all genes between pairwise samples. While the statistic rigorousness of a differential expression analysis (as above) is dismissed, such an analysis has the potential to give a wider perspective, as not only samples with replicates can be included. Indeed, the same pattern as above can be seen not only for four of the cell lines, but U2OS as well - the same holds true for correlations of only the prognostic markers (SFigure 15 and 16). These results demonstrate that the genetic heterogeneity present in cell populations has a profound effect on gene expression, both on the global level and for cancer-related genes.

In order to more fully investigate the possible origins of the genetic heterogeneity, we hypothesised that it might be due to genetic drift through either genomic instability, many years of culturing in and between different laboratories, or both. If so, a general accumulation of mutations is to be expected, particularly for low impact variants. As can be seen in Fig. 3E, the MODIFIER impact category has the lowest score in four of the cell lines, with a pronounced drop for HeLa in particular (and HCT116 to a lesser degree), indicating a higher proportion of mismatched lower impact variants. Indeed, HeLa has the highest proportions of MODIFIER variants, in addition to being the only cell line to have proportionally more MODERATE than LOW mismatching SNVs, possibly due to its ability to produce heterogeneously stable cell populations (SFigure 17D). Interestingly, the impact distribution of U2OS is clearly divergent, showing more mismatched HIGH impact variants, indicating that it might have larger phenotypic variation than the other cell lines (SFigure 17H).

Common causes of genetic drift are chromosomal instability (CIN) and abnormal chromosomal numbers (aneuploidy), both of which are recognised as hallmarks of cancer and are often found together^32,33^. In order to further investigate the presence of CIN and aneuploidy, we analysed the GEO data with an orthogonal RNA-seq method, which examines the *allelic ratio* (defined as *major allele* ÷ *minor allele*) across the entire transcriptome^34^. The mean allelic ratio of H9 and HCT116 (approximately 1.7) is significantly different from that of the other cell lines (closer to 2.0; Fig. 3F), indicating that a lower level of chromosomal aberrations may be the cause of their relative genomic stability compared to other cell lines (confidence intervals for each comparison are shown in SFigure 18). These results the genetic heterogeneity in public cell line data which can have profound effects on gene expression measurements.

## Discussion

A growing number of researchers use publicly available expression data to compare with their own results, but the accuracy of such analyses have yet to be assessed on a large scale. An increasingly apparent problem is that of the biological source of the samples used, as the results may be inaccurate or skewed if the datasets being compared are not genetically equivalent. While this applies to any kind of biological sample studied it is especially evident in cell line research, where the prevalence of contaminated, misidentified and otherwise unauthentic cells is increasing^8^. We thus sought to evaluate the comparability, authenticity and heterogeneity of RNA-seq cell line population data deposited in the GEO database using the methodology previously described^17^ on a large scale, performing several thousands of pairwise comparisons of high-quality SNVs across 139 samples and 85 datasets from eight different cell lines of varying origin.

While comparing SNVs found in a dataset to those in the COSMIC database can be used for cell authentication, it is problematic for cell lines with fewer COSMIC variants; the H9 cell line with its practically non-existent COSMIC overlap is the prime (but not only) example of this. Any result based on a small number of variants is less reliable than one based on many variants; the A549 cell line, for example, has below 50 overlapping COSMIC variants in four out of seven datasets. The reasons for the low COSMIC overlap for the H9 cell line are unclear; it has the third most SNVs of all studied cell lines, and the transcriptome-wide analysis indicate that they are highly similar. An analysis of various COSMIC metadata for each cell line highlights H9 and HeLa as having an almost zero proportion of “verified” variants, *i*.*e*. variants that have been found in more than one dataset (SFigure 19). While this might be explained by numerous existing strains for HeLa, not so for H9. The reason for the low overlap of H9 may then be a combination of few COSMIC variants and a high level of unverified variants. Another issue with COSMIC authentications is the fact that most of the mismatched variants have a homozygous genotype according to the transcriptomic data. This is possibly due to allele-specific expression, which has been shown to be common and highly variable in humans, occurring in up to 22% of SNVs in human cell lines^35,36^. Such homozygous variants are thus expected when using a genome-based database like COSMIC, but make the results more difficult to assess. As the COSMIC database is manually curated, a positive result could be sufficient as an assessment of cell line authenticity, but drawing definitive conclusions from small variant sets remains problematic.

In order to alleviate these problems, we performed transcriptome-wide analyses on all the datasets investigated in this study in a pairwise manner, thus including the totality of SNVs and possible biological variation in each dataset. The most striking result is as previously mentioned for the H9 cell line, which has the highest median transcriptome-wide concordance of all the cell lines investigated (reaching 99.6% for several pairs), clearly showing that its datasets most likely originate from biologically equivalent sources; similar results were found for HCT116. While the concordance is a useful measure of similarity, it doesn’t account for cases with few variant overlaps, such as for the three datasets from H9, HeLa and MCF7. By unfavourably weighing the concordances of comparisons with few overlaps these datasets are more easily incorporated into the analysis, enabling consideration of a single parameter (the similarity score) rather than two (concordance and overlap). The discrepancy between the GEO metadata and the detailed information available in the corresponding publications of these datasets demonstrate the difficulty of performing a bioinformatic analysis across experiments, but also highlights how important dataset assessment is; such information is vital to any researcher wishing to analyse publicly available data.

Another striking finding is that a single MCF7 dataset is unequivocally more similar to HCT116 than to the other MCF7 datasets. There is no published article associated with this dataset, making a thorough investigation of the reasons for this disparity difficult to perform. It does, however, present a clear-cut case where use of public datasets without proper evaluation can lead researchers to draw erroneous conclusions. If such a dataset was to be used for *e*.*g*. a baseline for MCF7 cell lines against a drug treatment or for direct comparisons with other breast cancer cell lines the results would be meaningless at best and potentially disastrous at worst. Our analysis does not dismiss the data itself in its entirety, far from it: seeing as this dataset so clearly comes from HCT116 cells it could potentially be incorporated into experimental designs where this would be appropriate.

The stability of H9 and HCT116 has been previously shown, corroborating our results^37,38^. The variation seen in HeLa is unsurprising, given its history of producing stable, heterogenous cell populations^39^. While variations in HeLa’s karyotype have been inconsistently reported, it is clear that such changes have an effect on the phenotype of the cells^40^. Indeed, HeLa, MCF7 and MDAMB231 have been established to be genetically unstable^38,41,42^. While the datasets analysed herein were not chosen with suitability for differential expression as primary criteria, the correlations of similarity score versus the number of DEGs and fold change indicate that the genetic heterogeneity observed has a significant effect on gene expression in the H9, HCT116, MCF7, MDAMB231 and U2OS cell lines. This was also seen for cancer-related genes, as upwards of several hundreds of prognostic markers were differentially expressed between genetically dissimilar same-cell datasets. The magnitude of the DEGs are also significantly affected, as the median fold change for the same datasets can reach as high as well above five. While neither date of submission nor number of overlapping variants were significant confounding factors for this analysis, there might be others. As this analysis is performed on the SNV-level, larger copy number variations (CNVs) might affect the analysis. The mean allelic ratios for H9 and HCT116 does, however, indicate that this is likely not the case. Experimental factors not included in the GEO metadata might also play a role, such as RNA quality or the polymerase used for library preparation^43^. These results demonstrate that the genetic heterogeneity also affects genes that are likely to be key components of cancer research, thus representing a critical confounding factor for such studies. While the number of datasets and replicates puts a limitation on the enrichment analysis, there are indications that the genetic heterogeneity also has an effect on general cellular functions. These conclusions are corroborated by gene expression correlations, both globally and for the prognostic markers.

We hypothesised that this heterogeneity could be due to genetic drift as a result of genetic instability, supported by a greater proportion of mismatched low impact variants. The genetic instability that leads to genetic drift and accumulation of mutations in cancer may have several causes, commonly including chromosomal instability and aneuploidy^33^. The analysis of mean allelic ratios separate H9 and HCT116 as having fewer chromosomal aberrations than the others, explaining their high stability across datasets. Such biological information is highly pertinent for any researcher about to start a cell line-based study: what level of genetic stability is relevant for the scientific question examined? A stable cell line may be more suitable for studies examining a specific gene or pathway (increased comparability of datasets), while a more varied cell line might be more applicable for drug screenings (taking greater biological diversity into account). It is thus not only the patient- or organ-of-origin of a cell line that is important: careful consideration of the overall goal of the experimental design also needs to be taken into account. Additionally, scientific findings obtained using cell lines have to be carefully scrutinised in the light of the inherent intra- and inter-genetic variation encountered in cell line populations. The U2OS cell line, for example, has a higher proportion of mismatched higher impact variants than the others, meaning that special care needs to be taken to ensure that none of the variants affect genes important to the scientific question at hand. Such considerations highlight one of the major strengths of our general methodology: the ability to investigate the biological effects of any variant differing between samples. This is particularly relevant given the previously demonstrated varying phenotype for perfectly matched STR profiles of the MCF7 cell line^15^.

**Figure 4:**
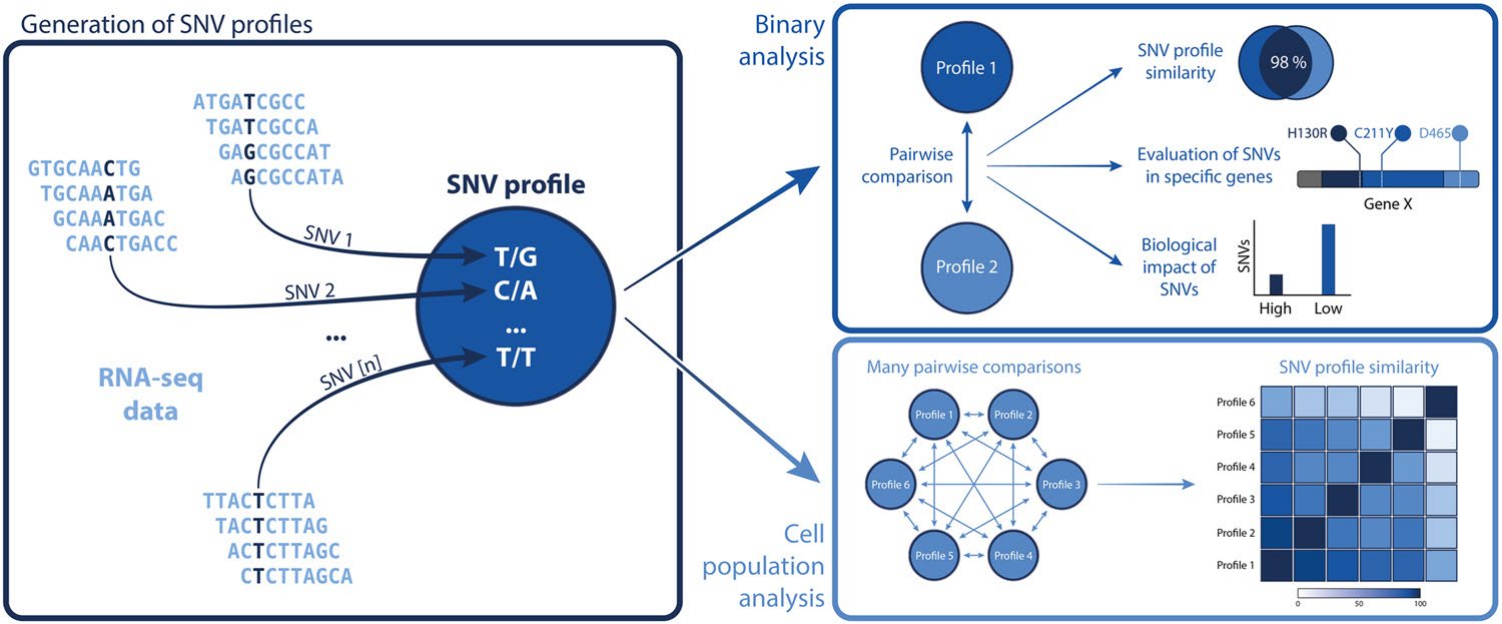
Representation of the general methodology. The workflow comprises several steps starting with the creation of an SNV profile by finding all variants in a given RNA-seq dataset (left panel). This SNV profile is then compared to other profiles, either in a pairwise manner (top right panel) or to many others in *e*.*g*. a population of datasets (bottom right panel). Results include global similarities between profiles, details on specific genes of interest and biological impact of SNVs differing across datasets, both for single- and population-scale analyses.

There are several factors that need to be considered in regards to the biological equivalency of two or more samples. The biology of any sample should be investigated by calculating its global similarity to a reference (preferably transcriptome-wide), if such is available. Any mismatched higher impact variants that might affect genes related to the biological question at hand need to be examined, as well as previously known variants. The impact of variants should also be investigated, in particular for those variants present in a pathway of interest. This is all given that the variant calling itself is performed with the most appropriate methods and software available, including choice of genome assembly and quality metrics, as done here. Not only cell line data could be analysed in this manner, but also data from tissues, cancer tumours or organoids. By following these guidelines (visualised in Fig. 4) researchers gain valuable information pertaining to heterogeneity, comparability and authenticity of their samples, leading to more well-informed choices regarding experimental designs and subsequently more biologically relevant conclusions.

In summary, we have performed a large-scale study of cell line heterogeneity in public RNA-seq data and highlighted important considerations for any researcher wishing to use such data in their analyses. We show that genetic heterogeneity in cell line populations has a great effect on gene expression measures as well as cancer hallmarks. The H9 and HCT116 cell lines are remarkably stable across many different studies and laboratories, possibly due to a smaller degree of chromosomal instability than the other cell lines (such as HeLa). We have demonstrated the importance of evaluating datasets by biological equivalency on both global and gene level as well as data quality and experimental conditions. Our methodology can help scientists make informed decisions *vis-à-vis* biological equivalency between both new sequencing data and any of the numerous publicly available datasets, representing a step forward both in terms of analysing dataset comparability, cell line heterogeneity and the conclusions drawn from their experiments.

## Materials and Methods

### Filtration and selection of GEO data

Selection and acquisition of data from the GEO was performed using an array of tools and online software packages: the NCBI *E-utilities*^44^, the *GEOquery* R package^45,46^ and the *SRAdb* R package^47^. The GEO was queried with the NCBI *E-utilities* for human RNA-seq datasets, and the metadata contained in the associated SOFT format files were parsed and filtered using a custom R script together with *GEOquery*. The SOFT files from GEO contain detailed metadata on each dataset not easily accessible through the online queries (such as cell type and experimental methods), making them a good choice for selection and filtering of metadata in a large-scale, programmatic manner. Only cell lines with at least ten RNA-seq datasets in the GEO and available in the COSMIC database^18^ were considered. As metadata specific for the raw sequencing data (such as file size and the number of sequenced bases) is only available in the SRA, the SRA-specific metadata was collated with the GEO metadata using the *SRAdb* R package and the NCBI E-*utilities*. This process was performed on November 16th, 2016; the final metadata contains 80 different metadata fields per sample (such as study and sample IDs, protocols, treatments and sequencing platforms) and can be found in SData 1, while details on each cell line are available in SData 2.

### Analysis of genetic heterogeneity

Analysis of SNVs and cell line authentication of the biological samples analysed in the selected datasets was performed as previously described^17^. Briefly, raw data from each sample was downloaded from the SRA using the *fastq-dump* utility from the *SRA toolkit*, followed by read alignment, variant calling (including confident homozygous reference sites) and filtering using STAR, GATK and in-house scripts, respectively^46,48,49^. In addition to comparisons of cell line-specific SNVs that were downloaded from the COSMIC database, the transcriptome-wide variants were also compared in a pairwise manner to each other. All parts of the analysis utilised the *GRCh38* assembly. Files containing the results of both the COSMIC and transcriptome-wide comparisons are included in SData 3–6.

### Differential expression analysis

Gene expression estimation was performed using the *Kallisto* (0.43.0)^27^ and *TXimport* (1.2.0)^28^ software, followed by differential gene expression analysis with *edgeR* (3.16.1)^29^. Genes with fold changes greater than two and FDR ≤ 0.01 were counted as differentially expressed. The list of prognostic markers was downloaded from table S8 in the Pathology Atlas publication, and DEG analysis was performed as above^31^. Gene expression correlations were performed using log-normalisation of (TPM + 1). KEGG Module enrichment was performed using the *clusterProfiler* (3.5.5)^50^ R package. Data on DEGs, expression correlations and enrichments is available in SData 7–10, while a list of all prognostic markers is available in SData 11. The cell lines H9, K562 and U2OS were not used for investigating prognostic markers, as they do not have a corresponding HPA tissue.

### Analysis of chromosomal aberrations

The eSNP-Karyotyping analysis^34^ was performed as previously described on SNVs that passed all of the following GATK variant calling filtering criteria: Fisher strand value ≥ 30, quality by depth ≥ 2, clusters with no more than 2 variants within a 35 base pair window, total allelic depth ≥ 10 and minor allelic ratio ≥ 0.2. Moving medians of allelic ratios were calculated across all chromosomes, yielding a measure of the mean allelic ratio across the entire transcriptome. All statistical testing was performed at significance levels of 0.01; a detailed RMarkdown document for reproducing all figures is available in the supplementary information.

## Electronic supplementary material

Supplementary information
