# Supplementary material for: Analysis of public RNA-sequencing data reveals biological consequences of genetic heterogeneity in cell line populations
Source: Sci Rep. 2018 Jul 25;8:11226. doi: 10.1038/s41598-018-29506-3 (PMC6060100; doi:10.1038/s41598-018-29506-3)
Supplement: Supplementary file 1 — Supplementary information [file 41598_2018_29506_MOESM1_ESM.pdf]

Analysis of public RNA-sequencing data reveals biological consequences of genetic heterogeneity in cell line populations: Supplementary Information

Erik Fasterius[1] and Cristina Al-Khalili Szigyarto[1,2]

[1]School of Chemistry, Biotechnology and Health,
KTH Royal Institute of Technology, Stockholm, Sweden
[2]Science for Life Laboratory, KTH Royal Institute of Technology, Stockholm, Sweden

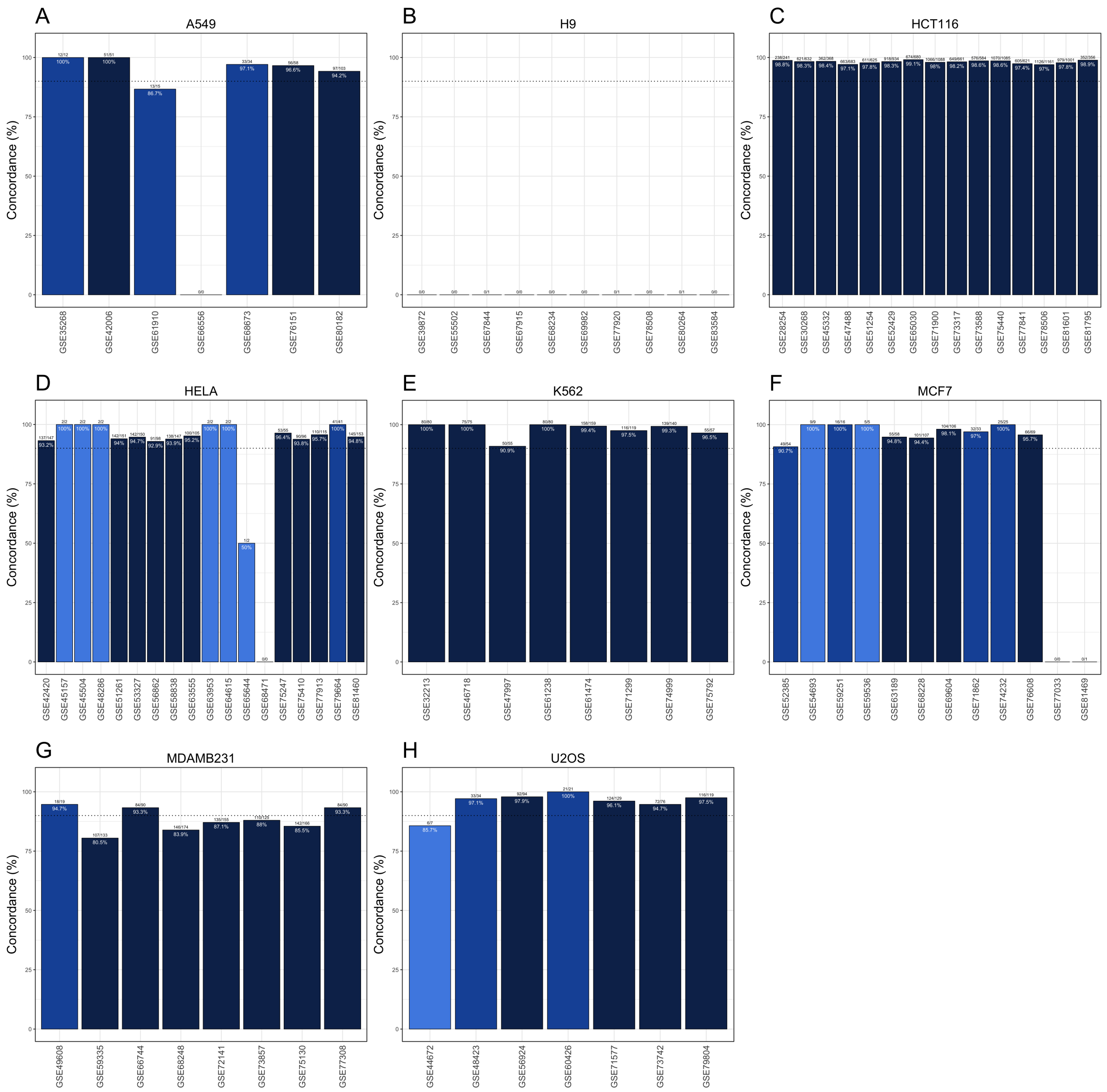

Supplementary Figure 1: COSMIC authentication overviews for each analysed cell line: **(A)** A549, **(B)** H9, **(C)** HCT116, **(D)** HeLa, **(E)** K562, **(F)** MCF7, **(G)** MDAMB231, **(H)** U2OS. Datasets with less than 10 overlapping variants are coloured light blue, while those with between 10 and 50 are coloured blue and those with 50 or more are coloured marine blue. The H9 cell line has no variants overlapping with COSMIC SNVs whatsoever, while most datasets have at least some overlap.

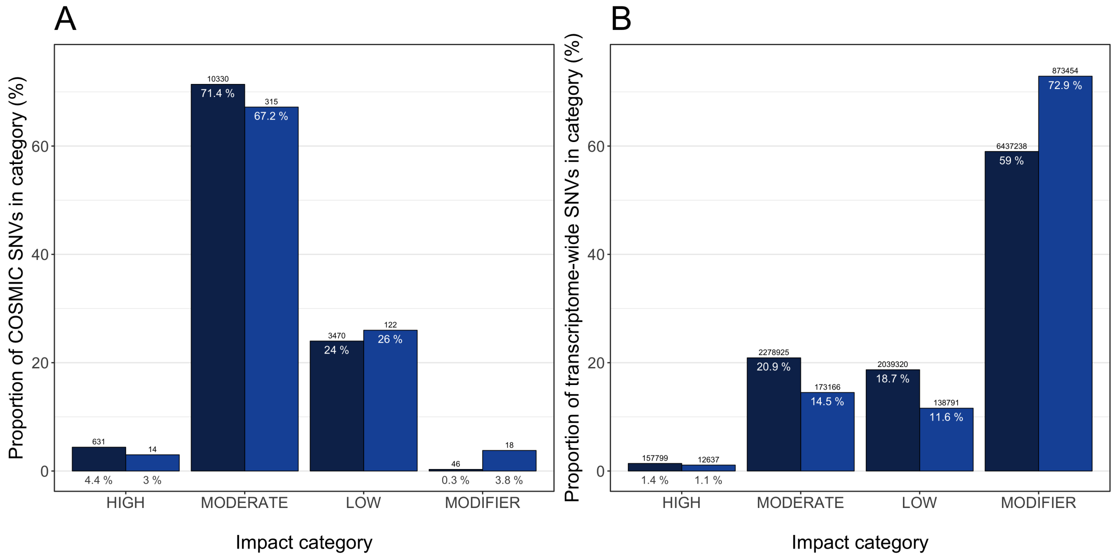

Supplementary Figure 2: Distribution of matching (marine blue) and mismatching (blue) of **(A)** COSMIC and **(B)** transcriptome-wide SNVs across the four impact categories for all the cell lines investigated in the study. As the COSMIC variants contain manually curated data from known mutations it is not surprising that its impact distribution is skewed towards the higher categories, while the transcriptome-wide distribution is more skewed towards low impact variants.

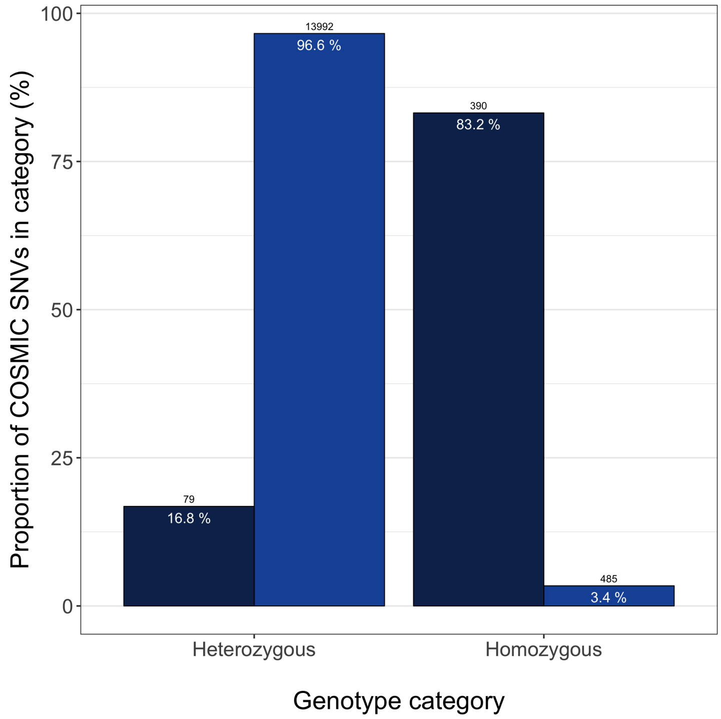

Supplementary Figure 3: Distribution of matching (marine blue) and mismatching (blue) heterozygous and homozygous variants in the RNA-seq data for COSMIC authentications in all the cell lines investigated in the study. While beyond the scope of this study, this could potentially be an indication of RNA-editing or allele-specific expression, *i.e.* due to comparing the mostly DNA-level data from COSMIC to RNA-seq variants.

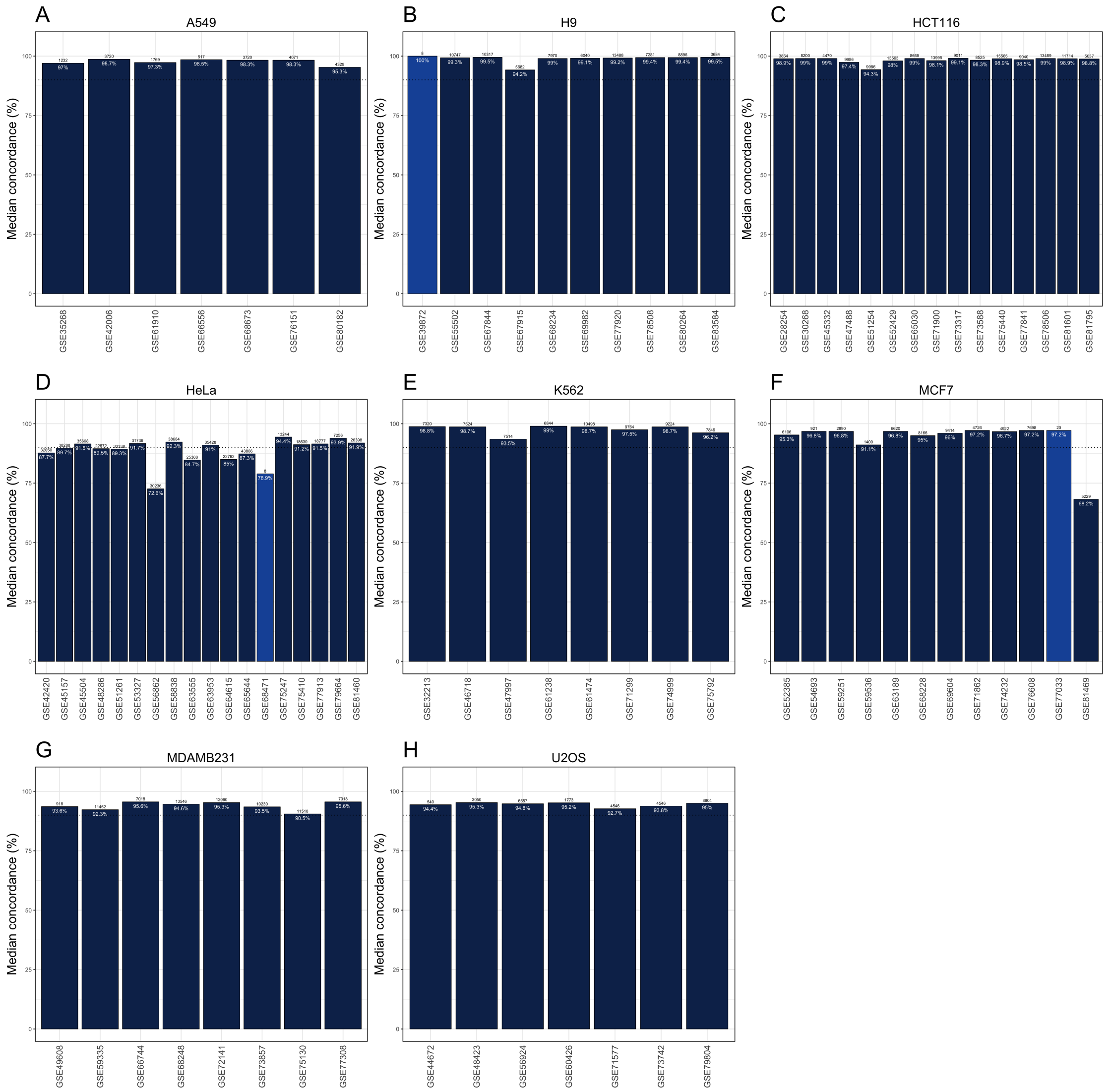

Supplementary Figure 4: Transcriptome-wide authentication overviews for each analysed cell lines: **(A)** A549, **(B)** H9, **(C)** HCT116, **(D)** HeLa, **(E)** K562, **(F)** MCF7, **(G)** MDAMB231, **(H)** U2OS. Datasets with less than 10 overlapping variants are coloured light blue, while those with between 10 and 50 are coloured blue and those with 50 or more are coloured marine blue. The H9 cell line that showed no overlap with the COSMIC variants whatsoever is here revealed to be remarkably stable for transcriptome-wide comparisons across all studied datasets (excepting one with low data quality).

Supplementary Table 1: Genotypes of all HCT116 datasets for the KRAS^{G13D} mutation (GRCh38: chromosome 12, position 25 245 347), expecting a heterozygous C/T genotype. Two datasets did not have any confident variant call for this position, and thus no conclusions can be drawn regarding their KRAS^{G13D} status, positive or otherwise.

Dataset	Allele 1	Allele 2	Depth	Allelic depth
GSE45332	C	T	9	[4, 5]
GSE47488	C	T	9	[4, 5]
GSE51254	C	T	22	[12, 10]
GSE52429	C	T	37	[25, 12]
GSE65030	C	T	10	[7, 3]
GSE71900	C	T	47	[28, 19]
GSE73317	C	T	18	[13, 5]
GSE73588	C	T	19	[8, 11]
GSE75440	C	T	76	[36, 40]
GSE77841	C	T	9	[5, 4]
GSE78506	C	T	25	[18, 7]
GSE81601	C	T	361	[188, 173]
GSE81795	C	T	7	[4, 3]

Supplementary figures 5 to 12 show transcriptome-wide, pairwise comparisons for all cell lines studied, individually. Similarity score below 50 is shown as pure white, with a grey colour gradient from 50 to 90 and a blue gradient up to 100.

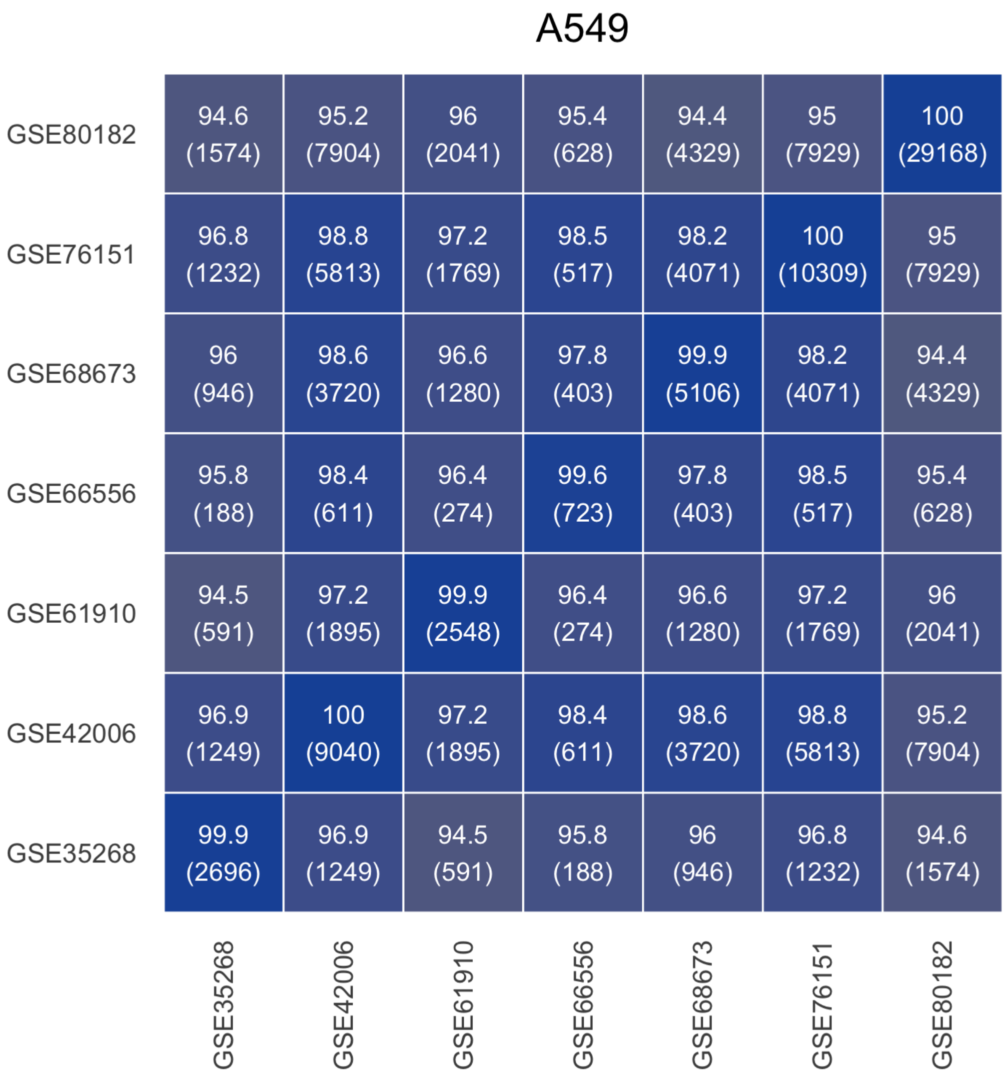

Supplementary Figure 5: Transcriptome-wide, pairwise comparisons for the A549 cell line.

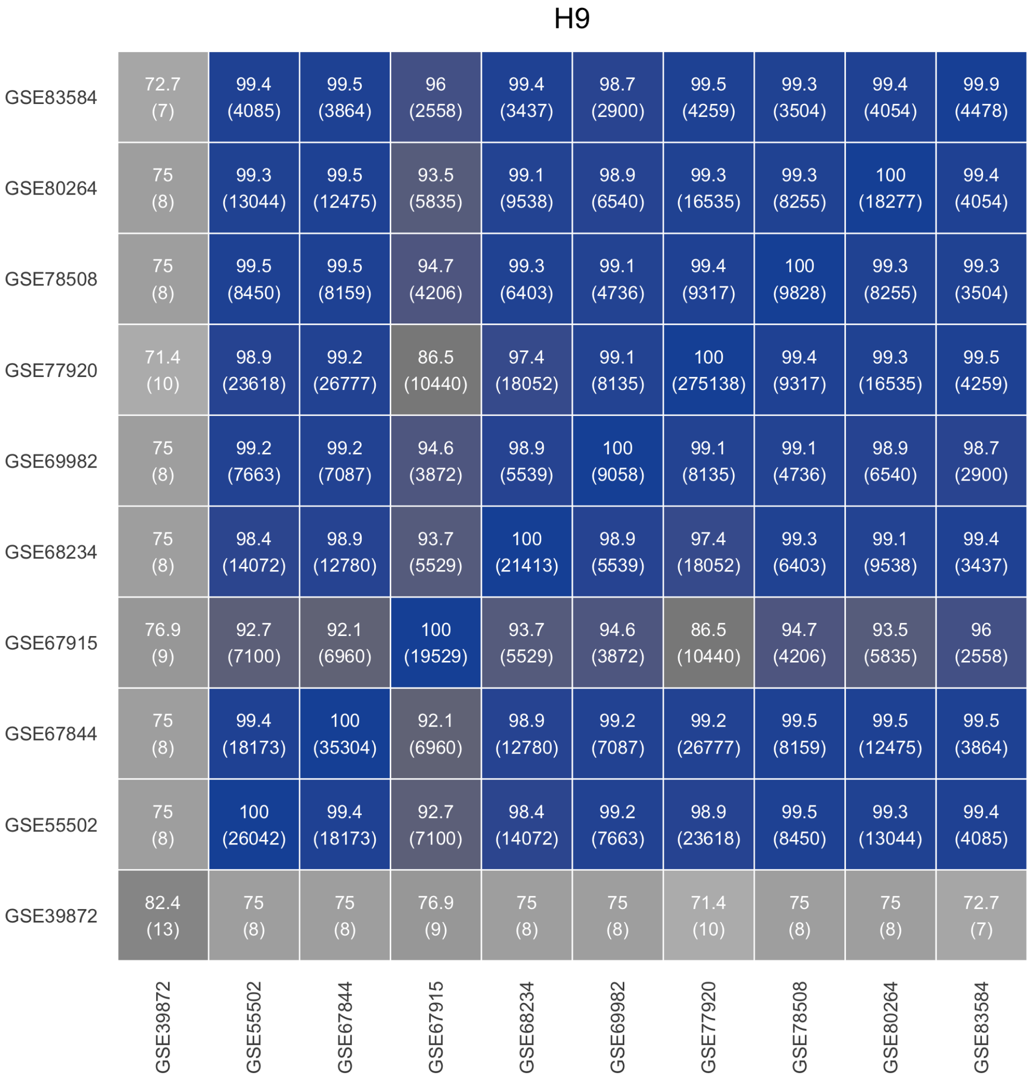

Supplementary Figure 6: Transcriptome-wide, pairwise comparisons for the H9 cell line.

HCT116

	GSE28254	GSE30268	GSE45332	GSE47488	GSE51254	GSE52429	GSE65030	GSE71900	GSE73317	GSE73588	GSE75440	GSE77841	GSE78506	GSE81601	GSE81795
GSE81795	98.6 (2484)	99 (4453)	98.7 (2745)	96.9 (4570)	93 (4158)	97.6 (5612)	98.9 (5107)	97.7 (5418)	99 (5025)	98.1 (4056)	99.1 (5527)	98.5 (5037)	99.2 (5490)	98.9 (5140)	99.9 (5915)
GSE81601	98.9 (3991)	99.1 (9029)	99.1 (5125)	97.4 (14101)	95.1 (13230)	98.4 (18458)	99.1 (9974)	98.4 (18508)	99.1 (10353)	98.4 (11714)	98.6 (22922)	98.5 (11275)	98.9 (20107)	100 (32465)	98.9 (5140)
GSE78506	98.9 (4103)	99.4 (10097)	99.1 (5332)	97.9 (15733)	95.7 (14829)	98.5 (21582)	99.3 (11364)	98.5 (22704)	99.3 (11519)	98.8 (13489)	99.1 (24924)	98.8 (12572)	100 (33423)	98.9 (20107)	99.2 (5490)
GSE77841	98.9 (3864)	98.9 (7985)	98 (4158)	97.7 (11030)	93.9 (7992)	97.7 (11884)	98.6 (8665)	97.5 (12025)	98.7 (9040)	97.6 (7678)	98.6 (11860)	100 (15351)	98.8 (12572)	98.5 (11275)	98.5 (5037)
GSE75440	98.9 (4253)	99.4 (9557)	99.2 (5564)	97.7 (15565)	96.8 (18310)	98.7 (24142)	99.2 (11212)	98.7 (22838)	99.4 (11288)	98.9 (15688)	100 (87571)	98.6 (11860)	99.1 (24924)	98.6 (22922)	99.1 (5527)
GSE73588	97.6 (3602)	98.5 (5764)	98.2 (4636)	96 (8525)	91.9 (13779)	96.5 (13563)	98.3 (8012)	96.9 (13995)	98.6 (7733)	100 (26273)	98.9 (15688)	97.6 (7678)	98.8 (13489)	98.4 (11714)	98.1 (4056)
GSE73317	99.1 (3591)	99.4 (8200)	99.1 (4313)	98 (8847)	94.7 (7779)	98.5 (11368)	99.2 (9011)	98.5 (11389)	100 (12065)	98.6 (7733)	99.4 (11288)	98.7 (9040)	99.3 (11519)	99.1 (10353)	99 (5025)
GSE71900	97.5 (4096)	98.6 (9927)	98 (5225)	95.5 (16079)	93.1 (15705)	96.9 (21512)	98.2 (11092)	100 (40944)	98.5 (11389)	96.9 (13995)	98.7 (22838)	97.5 (12025)	98.5 (22704)	98.4 (18508)	97.7 (5418)
GSE65030	98.8 (3450)	99.1 (7608)	98.9 (4278)	97.6 (8512)	94.6 (7883)	98.4 (10916)	100 (12266)	98.2 (11092)	99.2 (9011)	98.3 (8012)	99.2 (11212)	98.6 (8665)	99.3 (11364)	99.1 (9974)	98.9 (5107)
GSE52429	97.5 (4111)	98.5 (9838)	98 (5220)	95.5 (15938)	91.9 (14452)	100 (46738)	98.4 (10916)	96.9 (21512)	98.5 (11368)	96.5 (13563)	98.7 (24142)	97.7 (11884)	98.5 (21582)	98.4 (18458)	97.6 (5612)
GSE51254	93.2 (3578)	94.8 (5994)	94.2 (4470)	90 (9986)	100 (67446)	91.9 (14452)	94.6 (7883)	93.1 (15705)	94.7 (7779)	91.9 (13779)	96.8 (18310)	93.9 (7992)	95.7 (14829)	95.1 (13230)	93 (4158)
GSE47488	97.4 (4049)	97.8 (8477)	97.2 (3988)	100 (51201)	90 (9986)	95.5 (15938)	97.6 (8512)	95.5 (16079)	98 (8847)	96 (8525)	97.7 (15565)	97.7 (11030)	97.9 (15733)	97.4 (14101)	96.9 (4570)
GSE45332	98.9 (2423)	99.1 (3667)	100 (6155)	97.2 (3988)	94.2 (4470)	98 (5220)	98.9 (4278)	98 (5225)	99.1 (4313)	98.2 (4636)	99.2 (5564)	98 (4158)	99.1 (5332)	99.1 (5125)	98.7 (2745)
GSE30268	98.8 (3143)	100 (10528)	99.1 (3667)	97.8 (8477)	94.8 (5994)	98.5 (9838)	99.1 (7608)	98.6 (9927)	99.4 (8200)	98.5 (5764)	99.4 (9557)	98.9 (7985)	99.4 (10097)	99.1 (9029)	99 (4453)
GSE28254	99.9 (4971)	98.8 (3143)	98.9 (2423)	97.4 (4049)	93.2 (3578)	97.5 (4111)	98.8 (3450)	97.5 (4096)	99.1 (3591)	97.6 (3602)	98.9 (4253)	98.9 (3864)	98.9 (4103)	98.9 (3991)	98.6 (2484)

Supplementary Figure 7: Transcriptome-wide, pairwise comparisons for the HCT116 cell line.

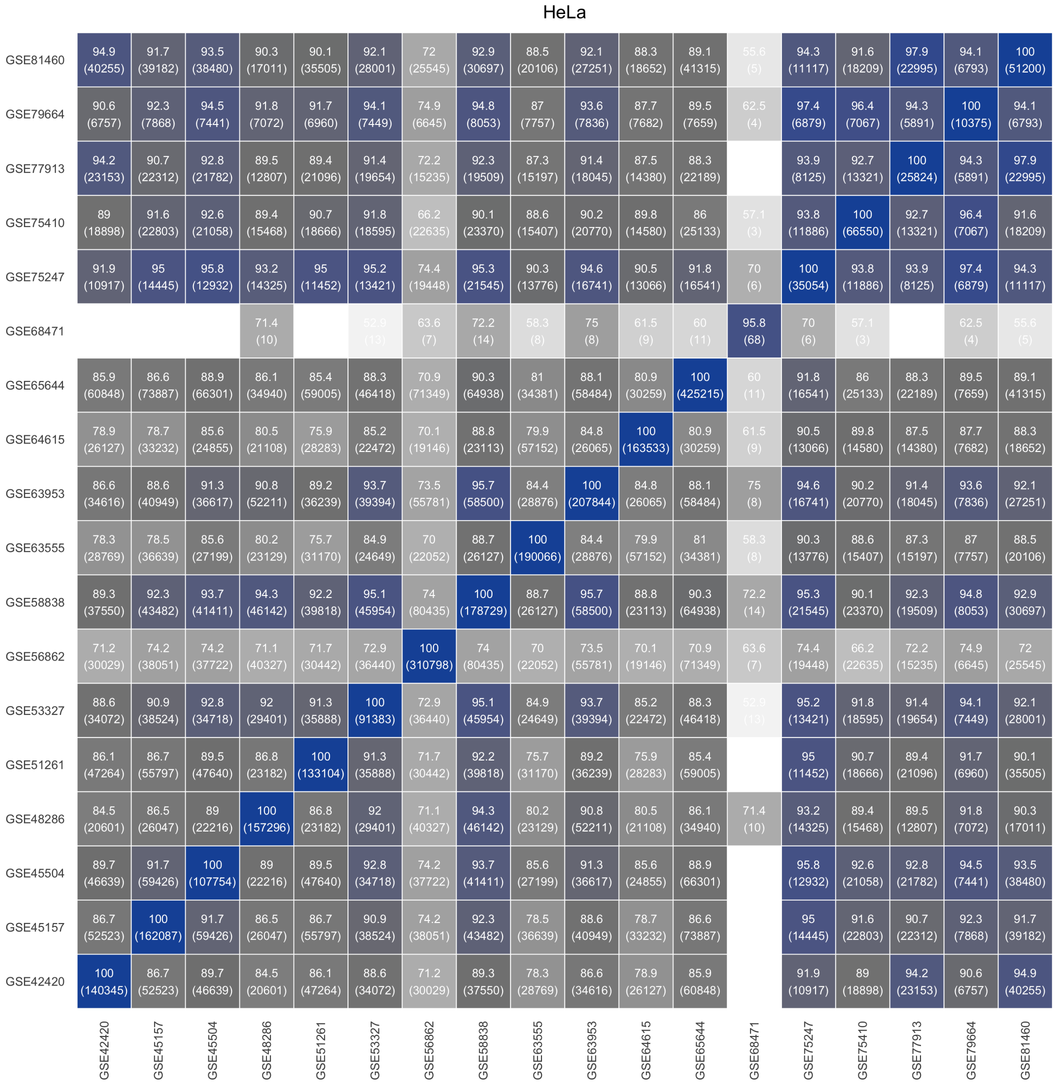

Supplementary Figure 8: Transcriptome-wide, pairwise comparisons for the HeLa cell line.

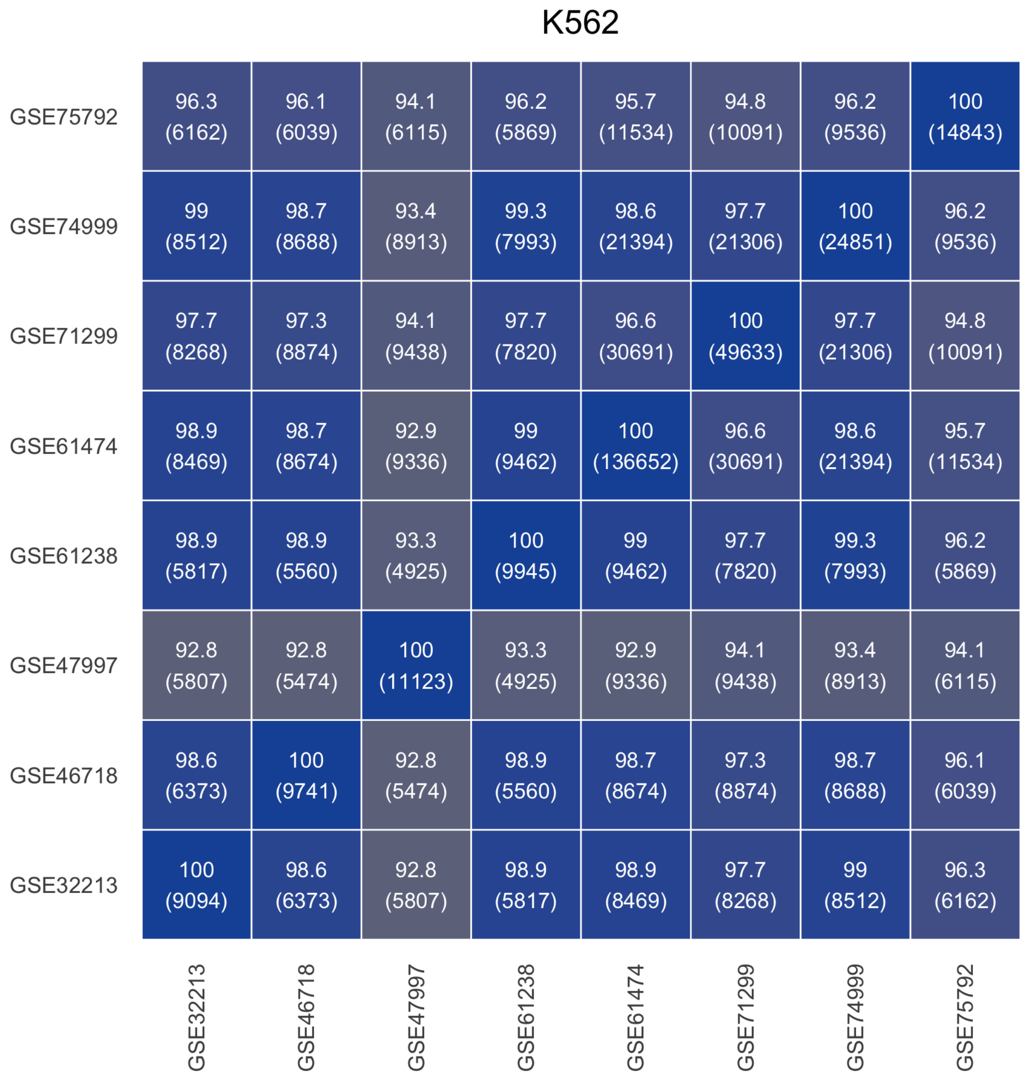

Supplementary Figure 9: Transcriptome-wide, pairwise comparisons for the K562 cell line.

MCF7

	GSE52385	GSE54693	GSE59251	GSE59536	GSE63189	GSE68228	GSE69604	GSE71862	GSE74232	GSE76608	GSE77033	GSE81469
GSE81469	67.3 (6413)	68.5 (567)	68 (1973)	68.3 (1017)	67.1 (7435)	67.9 (9489)	68.3 (11453)	67.2 (3662)	67.6 (4045)	69 (8609)	66.7 (23)	100 (22771)
GSE77033	70.4 (23)			62.5 (12)	66.7 (5)	61.5 (22)	64.7 (30)	57.1 (3)	81.8 (18)	89.7 (25)	93.5 (42)	66.7 (23)
GSE76608	95.4 (10642)	97 (1054)	97.9 (3267)	91 (1688)	97.2 (12833)	95 (16399)	96 (21447)	98 (6725)	97.9 (6787)	100 (24331)	89.7 (25)	69 (8609)
GSE74232	95.3 (5799)	96.8 (783)	97.2 (2683)	91.2 (1496)	97.1 (5806)	95.1 (6842)	96 (7374)	97.8 (3987)	100 (8296)	97.9 (6787)	81.8 (18)	67.6 (4045)
GSE71862	95.2 (5465)	97.1 (902)	96.9 (2552)	89.2 (1145)	97.5 (5775)	95.1 (6346)	96.9 (6956)	100 (7955)	97.8 (3987)	98 (6725)	57.1 (3)	67.2 (3662)
GSE69604	95.7 (12775)	97.9 (1054)	96.6 (3340)	90.9 (1826)	96.1 (17131)	95.5 (24972)	100 (91174)	96.9 (6956)	96 (7374)	96 (21447)	64.7 (30)	68.3 (11453)
GSE68228	97.1 (11547)	96.2 (989)	94.9 (3226)	91.9 (1694)	94.4 (14052)	100 (28355)	95.5 (24972)	95.1 (6346)	95.1 (6842)	95 (16399)	61.5 (22)	67.9 (9489)
GSE63189	95 (10125)	96.1 (969)	97.3 (3244)	90.8 (1305)	100 (18908)	94.4 (14052)	96.1 (17131)	97.5 (5775)	97.1 (5806)	97.2 (12833)	66.7 (5)	67.1 (7435)
GSE59536	91.4 (1561)	90.5 (376)	89.4 (845)	99.9 (2053)	90.8 (1305)	91.9 (1694)	90.9 (1826)	89.2 (1145)	91.2 (1496)	91 (1688)	62.5 (12)	68.3 (1017)
GSE59251	94.5 (3098)	95.4 (719)	99.9 (3521)	89.4 (845)	97.3 (3244)	94.9 (3226)	96.6 (3340)	96.9 (2552)	97.2 (2683)	97.9 (3267)		68 (1973)
GSE54693	95.2 (940)	99.7 (1131)	95.4 (719)	90.5 (376)	96.1 (969)	96.2 (989)	97.9 (1054)	97.1 (902)	96.8 (783)	97 (1054)		68.5 (567)
GSE52385	100 (13478)	95.2 (940)	94.5 (3098)	91.4 (1561)	95 (10125)	97.1 (11547)	95.7 (12775)	95.2 (5465)	95.3 (5799)	95.4 (10642)	70.4 (23)	67.3 (6413)

Supplementary Figure 10: Transcriptome-wide, pairwise comparisons for the MCF7 cell line.

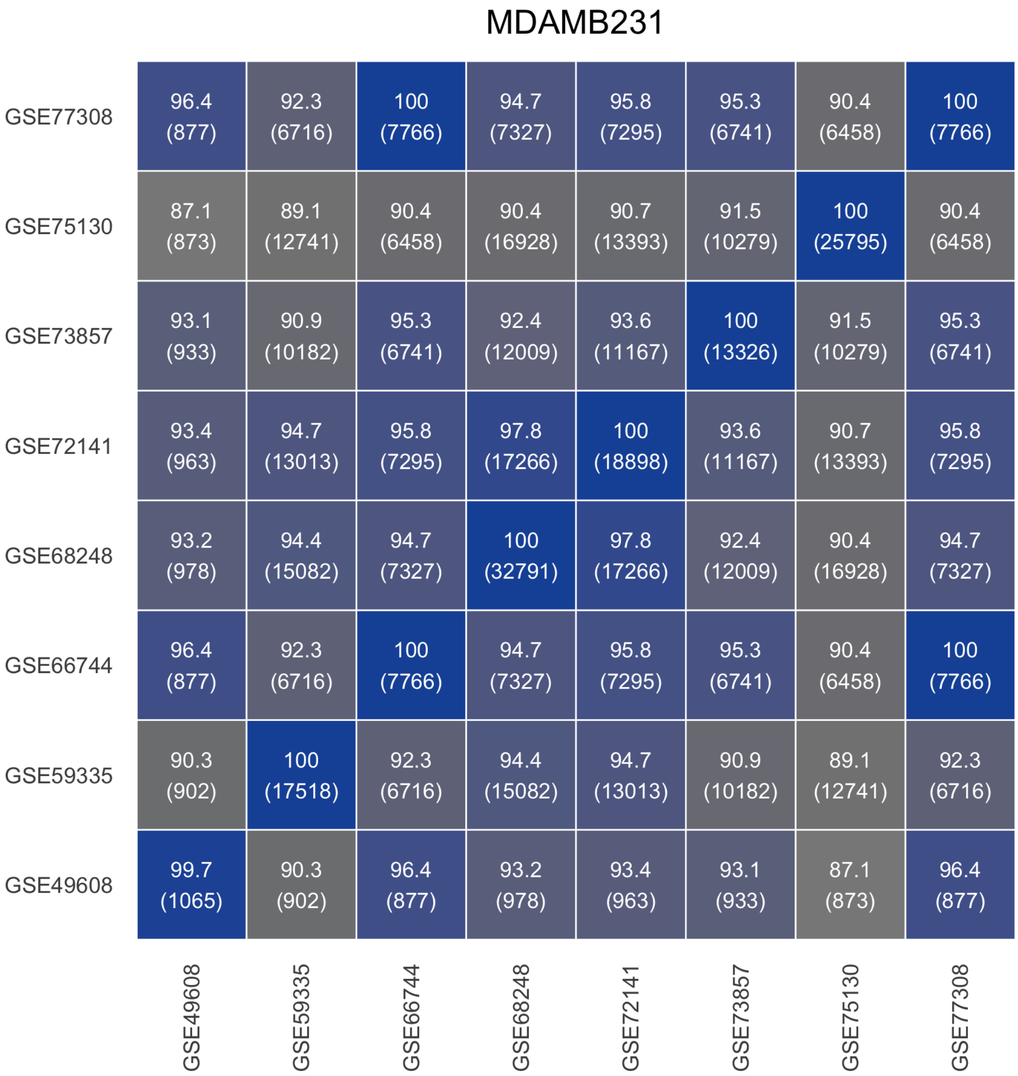

Supplementary Figure 11: Transcriptome-wide, pairwise comparisons for the MDAMB231 cell line.

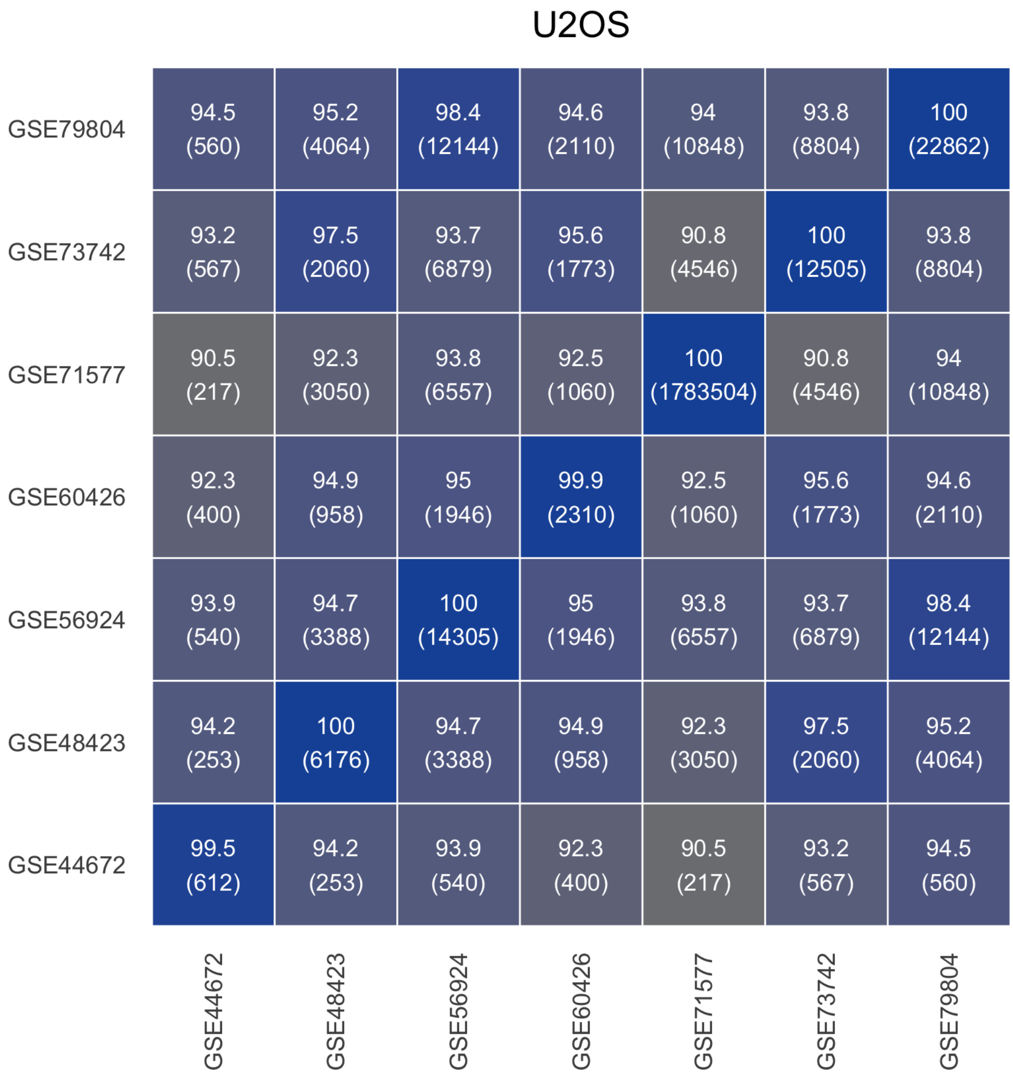

Supplementary Figure 12: Transcriptome-wide, pairwise comparisons for the U2OS cell line.

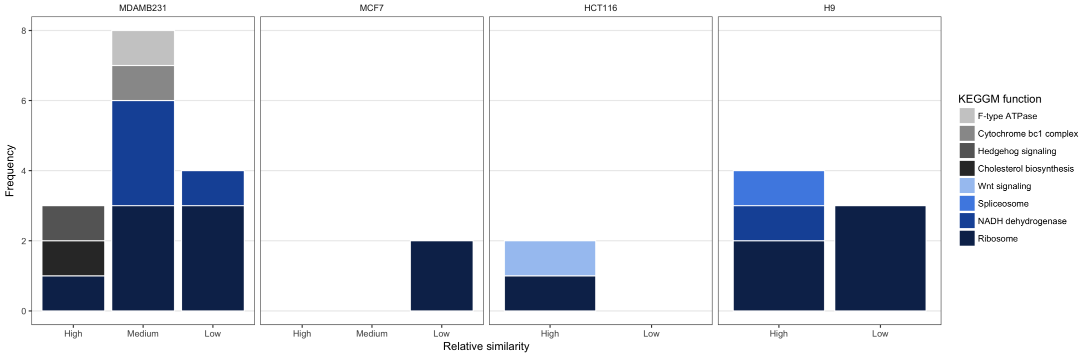

Supplementary Figure 13: KEGGM functional enrichment of differentially expressed genes. Each cell was divided into two or three groups of clustered, same-cell pairwise comparisons depending on the distribution of their respective similarity scores (see **Supplementary figure 14**) and their significance in the DEG correlation analyses, followed by enrichment analysis of KEGGM functional units. There is a greater number of enriched KEGG modules in the two lower similarity groups for MDAMB231 and MCF7 (*i.e.* "Medium" and "Low"), while there are few or none for the "High" group. Both groups are similar for H9, while only the "High" group in HCT116 contain any enrichments. Interestingly, the "Low" group in MCF7 consists of only 2 comparisons with similarity below 90, yet still yields significant enrichments. Pathways related to translation and ribosomal processes are present in all enriched groups (as expected from cancer-related cell lines), [1] while lipid metabolism, the Hedgehog pathway and mitochondrial functions can be seen particularly in MDAMB231 (as expected from a breast cancer cell line). [2, 3, 4]

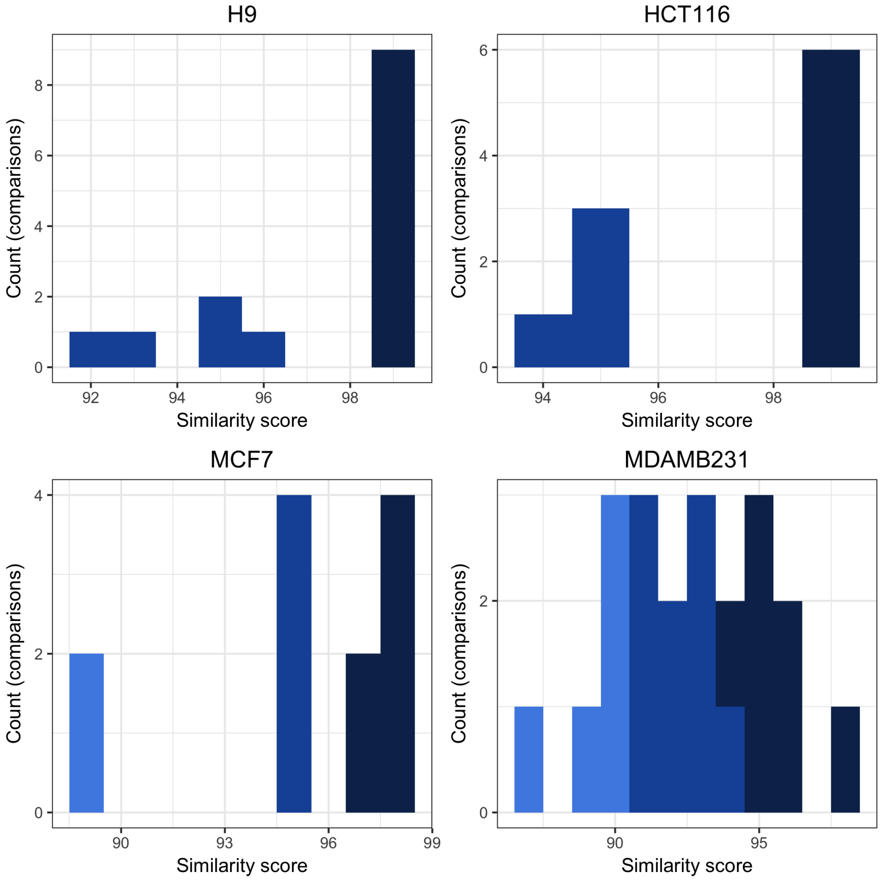

Supplementary Figure 14: Distributions of similarity scores for each cell line with significant correlations with either number of DEGs or fold change of DEGs (from **Figure 3** in the main article). While the MDAMB231 cell line has well-distributed dataset comparisons across the similarity score range, neither of the other three cell lines has such easily divisible data. It is of note, however, that the only significant enrichments for the MCF7 cell line (**Supplementary figure 13**) is for the lowest similarity group, even though it only contains two pairwise comparisons.

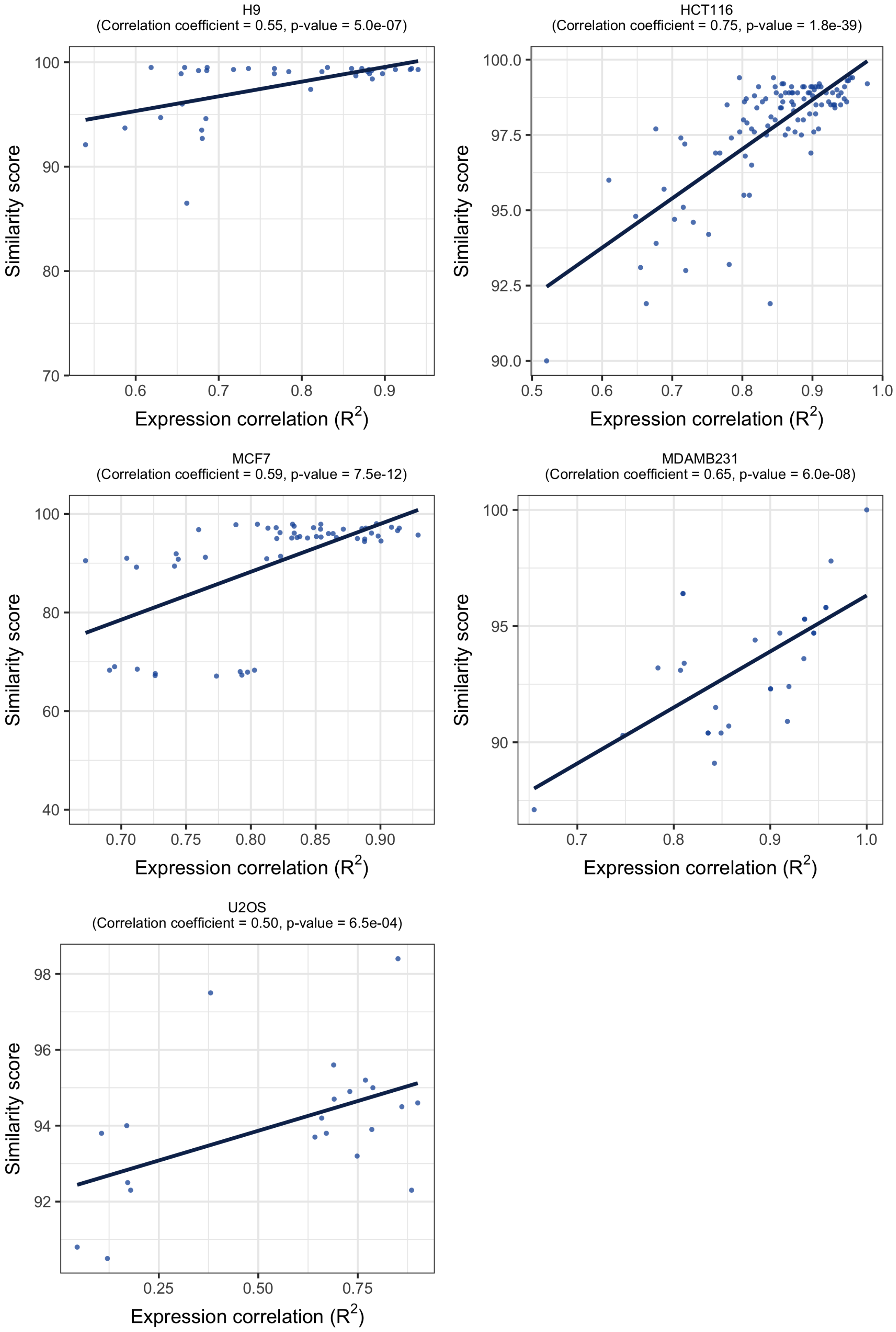

Supplementary Figure 15: Statistically significant correlations between the similarity score and same-cell pairwise gene expression correlations (Pearson's R^2) using log-normalised (TPM + 1); non-significant correlations were not visualised, but are still included in the analysis (see **Supplementary code**). This analysis corroborates the differential expression analysis (**Figure 3** in the main article) by using the Pearson correlation as a different measure of similarity, rather than the original parameters of number of DEGs and fold change of DEGs.

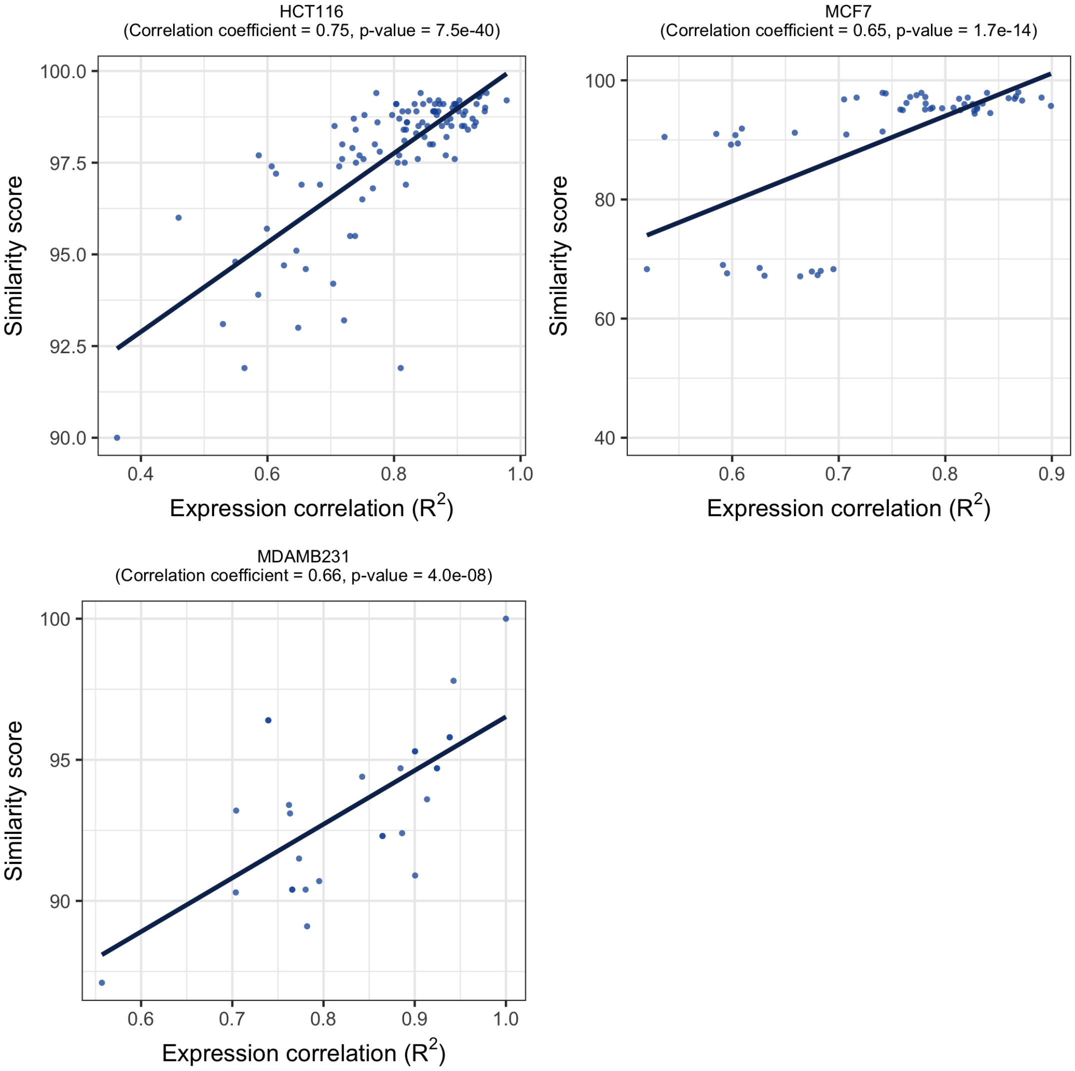

Supplementary Figure 16: Statistically significant correlations between the similarity score and same-cell pairwise gene expression correlations (Pearson's R^2) using log-normalised (TPM + 1) for all genes listed as prognostic markers in the HPA. The cell lines H9, K562 and U2OS were not included in this analysis, as they do not have a corresponding HPA tissue for which prognostic markers are listed. All other cells were included, but only statistically significant correlations were visualised (see **Supplementary code**).

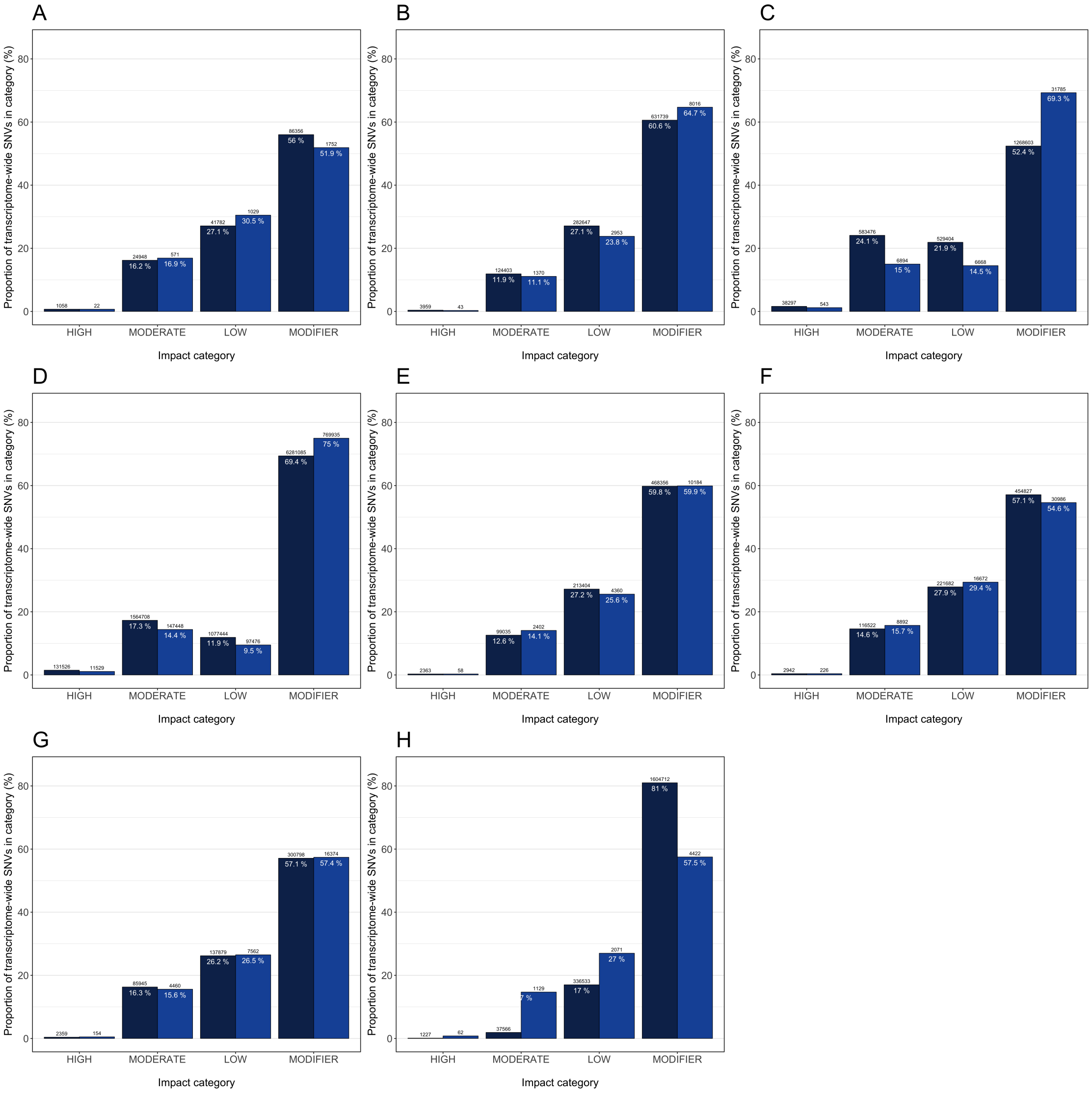

Supplementary Figure 17: Individual variant impact distributions across the different cell lines for the transcriptome-wide comparisons: **(A)** A549, **(B)** H9, **(C)** HCT116, **(D)** HeLa, **(E)** K562, **(F)** MCF7, **(G)** MDAMB231, **(H)** U2OS.

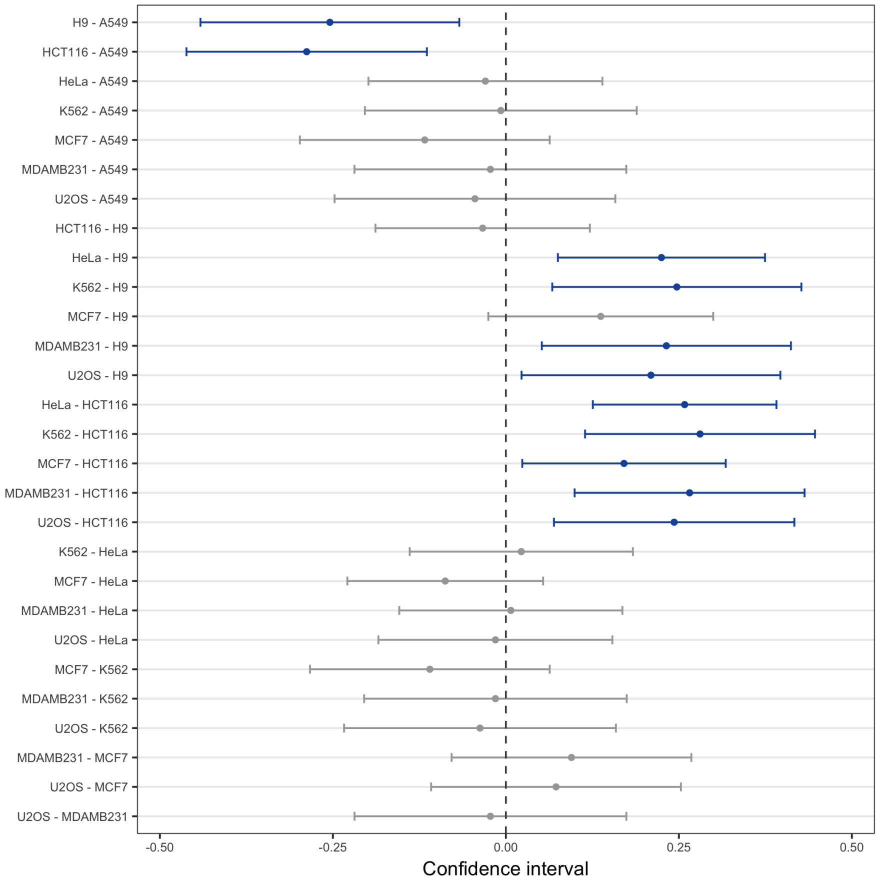

Supplementary Figure 18: Visualisation of ANOVA and Tukey's Honest Significant Difference 99 % confidence intervals. Significant intervals are coloured blue, while non-significant intervals (spanning zero) are grey.

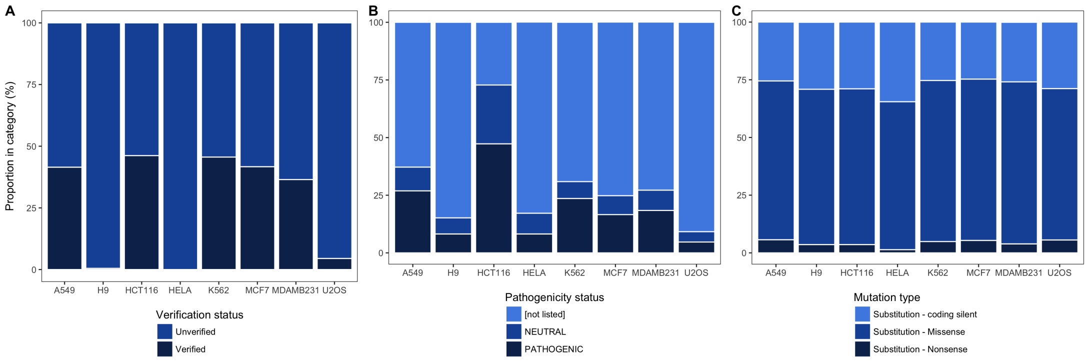

Supplementary Figure 19: Summary of all cell line-specific COSMIC SNV profiles and the different classes they belong to. **(A)** The verification status of COSMIC variants, *i.e.* if they are based on more than a single source or not; **(B)** the pathogenicity of COSMIC variants, *i.e.* if they have been shown to be pathogenic or not; **(C)** the type of mutations for the COSMIC variants, *i.e.* if they are silent, missense or nonsense.

Supplementary Table 2: Total number of datasets for each country of origin for all the datasets in the study.

Country	Datasets
USA	54
Germany	5
United Kingdom	4
Israel	3
Spain	3
Sweden	3
China	2
Italy	2
Australia	1
Belgium	1
Finland	1
Japan	1
Netherlands	1
Singapore	1
South Korea	1
Switzerland	1
Taiwan	1

Supplementary Table 3: Correlations between the number of overlaps and the difference in GEO submission date for each same-cell, pairwise comparisons for the those cell lines that were significantly correlated with the DEG parameters.

		H9	HCT116	MCF7	MDAMB231	U2OS
Overlaps	Correlation	0.132	-0.071	0.250	0.018	0.312
	P-value	0.268	0.304	0.018	0.897	0.044
GEO date	Correlation	0.139	-0.139	0.138	-0.111	-0.052
	P-value	0.244	0.044	0.194	0.417	0.746

References

[1] Stumpf, C. R. and Ruggero, D. (August, 2011) The cancerous translation apparatus.. *Current opinion in genetics & development,* **21**(4), 474–483.

[2] Pitroda, S. P., Khodarev, N. N., Beckett, M. A., Kufe, D. W., and Weichselbaum, R. R. (April, 2009) MUC1-induced alterations in a lipid metabolic gene network predict response of human breast cancers to tamoxifen treatment.. *Proceedings of the National Academy of Sciences of the United States of America,* **106**(14), 5837–5841.

[3] Hui, M., Cazet, A., Nair, R., Watkins, D. N., O'Toole, S. A., and Swarbrick, A. (March, 2013) The Hedgehog signalling pathway in breast development, carcinogenesis and cancer therapy.. *Breast cancer research : BCR,* **15**(2), 203.

[4] Ma, Y., Bai, R.-K., Trieu, R., and Wong, L.-J. C. (January, 2010) Mitochondrial dysfunction in human breast cancer cells and their transmitochondrial cybrids.. *Biochimica et biophysica acta,* **1797**(1), 29–37.

Supplementary code

This R Markdown document demonstrates how to reproduce the figures included in the article "Analysis of public RNA-seq datasets reveal genetic heterogeneity in cell line populations". Data files *sdata.1* through *sdata.7* are available in the supplementary data folder, while the file *transcriptome.collected.txt* is not (due to its large size of more than 1 GB), but can be supplied upon request. Individual, non-aggregated files from differential expression analyses and KEGG enrichments are also available upon request, again due to file size.

Load required packages

```
library("dplyr")
library("ggplot2")
library("grid")
library("gridExtra")
library("multcompView")
library("reshape2")
library("scales")
library("tidyr")
```

Load data

```
# Read GEO metadata
metadata_full <- read.table("sdata.1.metadata.txt", sep = "\t", header = TRUE,
                            quote <- NULL, fill = TRUE)
metadata <- unique(metadata_full[c("GSE", "cell.line")])

# Read COSMIC data
data.cosmic <- read.table("sdata.3.cosmic.stats.txt", header = TRUE, sep = "\t")

# Read transcriptome data
data.transc <- read.table("sdata.4.transcriptome.stats.txt", header = TRUE,
                         sep = "\t")

# Read eSNP-Karyotyping data
data.esnp <- read.table("sdata.5.esnp.stats.txt", header = TRUE, sep = "\t",
                  stringsAsFactors = FALSE)

# Read COSMIC SNV data
data.cosmic.snvs <- read.table("sdata.6.cosmic.collected.txt", header = TRUE,
                              sep = "\t", stringsAsFactors = FALSE, fill = TRUE)

# Read DEG data
data.degs <- read.table("sdata.7.degs.txt", sep = "\t", header = TRUE,
                       stringsAsFactors = FALSE)

# Read prognostic DEG data
data.degs.prog <- read.table("sdata.8.prognostic.degs.txt", sep = "\t", header = TRUE,
                       stringsAsFactors = FALSE)
```

```r
# Load expression data
data.expr <- read.table("sdata.9.expression.correlations.txt", sep = "\t",
                        header = TRUE, stringsAsFactors = FALSE)

# Load expression data
data.expr.prog <- read.table("sdata.10.expression.correlations.prognostic.txt",
                             sep = "\t", header = TRUE, stringsAsFactors = FALSE)
```

```r
# Load transcriptome SNV data
data.transc.snvs <- read.table("transcriptome.collected.txt", header = TRUE,
                               sep = "\t", stringsAsFactors = FALSE)
```

Main article figures

Figure 1

Boxplots of both COSMIC and transcriptome-wide variants and concordances.

```r
# Keep only comparisons within same cell lines
data <- data.transc[data.transc$cell.1 == data.transc$cell.2, ]

# Remove comparisons of the same series
data <- data[data$sample.1 != data$sample.2, ]

# Remove duplicated comparisons
data <- data[!duplicated(data[c("calls", "matches", "concordance", "score")]), ]

# Plot boxplot of all COSMIC calls
gg.ccalls <- ggplot(data.cosmic, aes(x = cell.line, y = cosmic.calls)) +
    stat_boxplot(geom = "errorbar", width = 0.15) +
    geom_boxplot(fill = "grey90", outlier.shape = NA) +
    geom_point(data = data.cosmic[data.cosmic$cosmic.calls == 0, ], colour = "black",
               alpha = 0.75, position = position_jitter(w = 0.3, h = 0), size = 1) +
    geom_point(data = data.cosmic[data.cosmic$cosmic.calls < 10 &
                                  data.cosmic$cosmic.calls != 0, ],
               shape = 21, stroke = 0.5, colour = "black", fill = "white", alpha = 0.75,
               position = position_jitter(w = 0.3, h = 0), size = 1) +
    geom_point(data = data.cosmic[data.cosmic$cosmic.calls >= 10, ], shape = 21,
               colour = "#0d2d59", fill = "#1954a6", stroke = 0.5, alpha = 0.75,
               position = position_jitter(w = 0.3, h = 0), size = 1) +
    theme_bw() +
    theme(legend.position = "none", axis.text = element_text(size = 11),
          plot.margin = unit(c(40, 5, 5, 5), "pt"),
          axis.title = element_text(size = 15)) +
  labs(x = NULL, y = "Number of COSMIC variants")

# Plot boxplot of all COSMIC concordances
gg.cconc <- ggplot(data.cosmic, aes(x = cell.line, y = cosmic.concordance)) +
    stat_boxplot(geom = "errorbar", width = 0.15) +
    geom_boxplot(fill = "grey90", outlier.shape = NA) +
    geom_point(data = data.cosmic[data.cosmic$cosmic.calls == 0, ], colour = "black",
               alpha = 0.75, position = position_jitter(w = 0.3, h = 0), size = 1) +
    geom_point(data = data.cosmic[data.cosmic$cosmic.calls < 10 &
                                    data.cosmic$cosmic.calls != 0, ],
               shape = 21, stroke = 0.5, colour = "black", fill = "white", alpha = 0.75,
               position = position_jitter(w = 0.3, h = 0), size = 1) +
    geom_point(data = data.cosmic[data.cosmic$cosmic.calls >= 10, ], shape = 21,
               colour = "#0d2d59", fill = "#1954a6", stroke = 0.5, alpha = 0.75,
               position = position_jitter(w = 0.3, h = 0), size = 1) +
    geom_hline(aes(yintercept = 90), colour = "black", linetype = "dotted") +
    theme_bw() +
    theme(legend.position = "none", axis.text = element_text(size = 11),
          plot.margin = unit(c(40, 5, 5, 5), "pt"),
          axis.title = element_text(size = 15)) +
  labs(x = NULL, y = "Concordance of COSMIC variants (%)")
```

```r
# Plot boxplot of all transcriptome calls
gg.tcalls <- ggplot(data, aes(x = cell.1, y = calls)) +
  stat_boxplot(geom = "errorbar", width = 0.15) +
  geom_boxplot(fill = "grey90", outlier.shape = NA) +
  geom_point(data = data[data$calls == 0, ], position = position_jitter(w = 0.3, h = 0),
             colour = "black", alpha = 0.75, size = 1) +
  geom_point(data = data[data$calls < 50 & data$calls != 0, ], shape = 21,
             colour = "black", fill = "white", position = position_jitter(w = 0.3, h = 0),
             alpha = 0.75, size = 1) +
  geom_point(data = data[data$calls >= 50, ], position = position_jitter(w = 0.3, h = 0),
             shape = 21, colour = "#0d2d59", fill = "#1954a6", stroke = 0.5, alpha = 0.75,
             size = 1) +
  theme_bw() +
  theme(legend.position = "none", axis.text = element_text(size = 11),
        plot.margin = unit(c(40, 5, 5, 5), "pt"),
        axis.title = element_text(size = 15)) +
  labs(x = NULL, y = "Number of transcriptome-wide variants")

# Plot boxplot of all transcriptome concordances
gg.tconc <- ggplot(data, aes(x = cell.1, y = concordance)) +
  stat_boxplot(geom = "errorbar", width = 0.15) +
  geom_boxplot(fill = "grey90", outlier.shape = NA) +
  geom_hline(aes(yintercept = 90), colour = "black", linetype = "dotted") +
  geom_point(data = data[data$calls == 0, ], position = position_jitter(w = 0.3, h = 0),
             colour = "black", alpha = 0.75, size = 1) +
  geom_point(data = data[data$calls < 50 & data$calls != 0, ], shape = 21,
             colour = "black", fill = "white", position = position_jitter(w = 0.3, h = 0),
             alpha = 0.75, size = 1) +
  geom_point(data = data[data$calls >= 50, ], position = position_jitter(w = 0.3, h = 0),
             shape = 21, colour = "#0d2d59", fill = "#1954a6", stroke = 0.5, alpha = 0.75,
             size = 1) +
  theme_bw() +
  theme(legend.position = "none", axis.text = element_text(size = 11),
        axis.title = element_text(size = 15),
        plot.margin = unit(c(40, 5, 5, 5), "pt")) +
  labs(x = NULL, y = "Concordance of transcriptome-wide variants (%)") +
  coord_cartesian(ylim = c(0, 100))

# Arrange on a 2x2 grid
fig_1 <- cowplot::plot_grid(gg.ccalls, gg.cconc, gg.tcalls, gg.tconc,
                   labels = c("A", "B", "C", "D"), label_size = 20,
                   nrow = 2, ncol = 2)
```

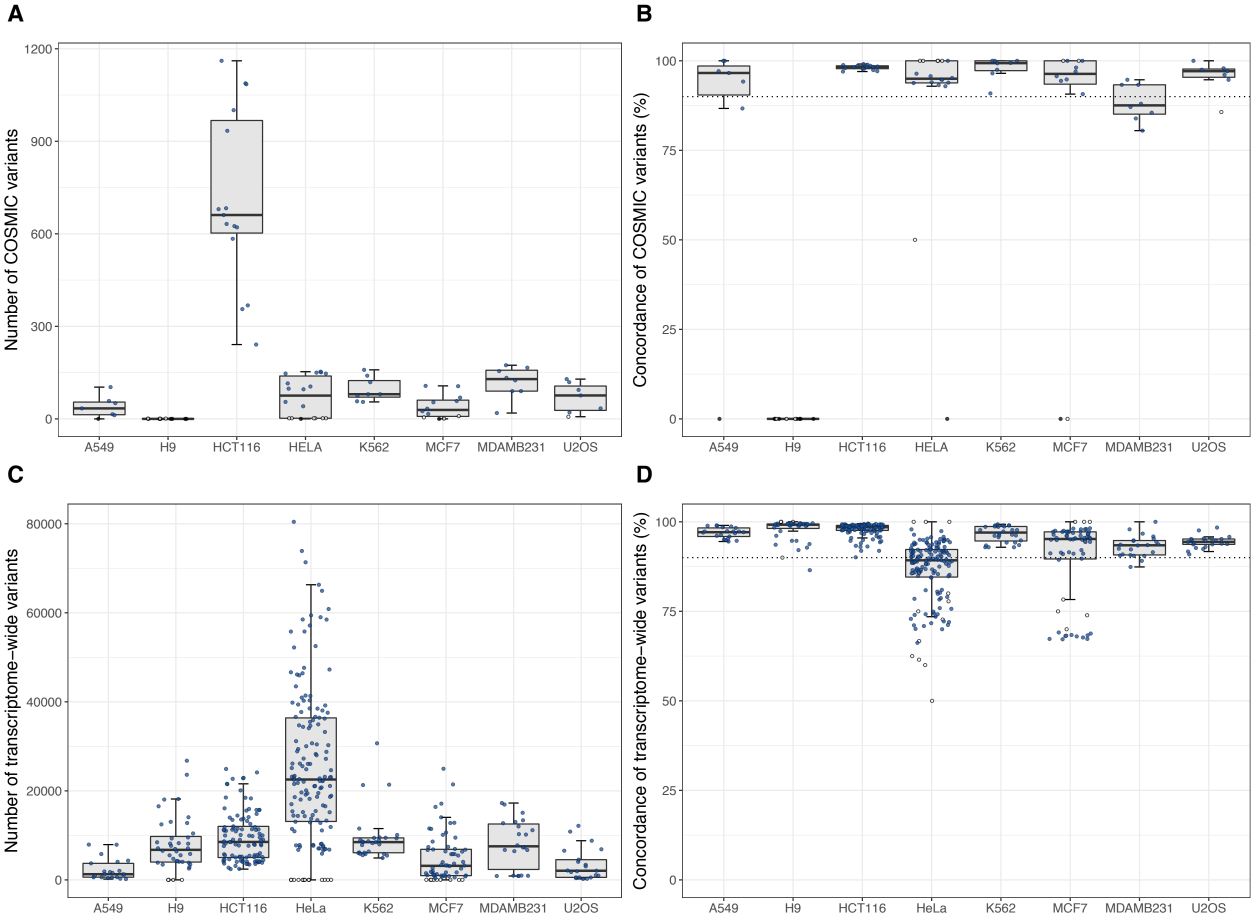
A
B
C
D
Number of COSMIC variants
Concordance of COSMIC variants (%)
Number of transcriptome-wide variants
Concordance of transcriptome-wide variants (%)
A549
H9
HCT116
HELA
HeLa
K562
MCF7
MDAMB231
U2OS
0
300
600
900
1200
25
50
75
100
20000
40000
60000
80000

Figure 2

Clustered heatmap of the transcriptome-wide analyses and results.

```r
# Add cell line name to GSE IDs
data <- data.transc
data$sample.1 <- paste0(data$cell.1, ": ", data$sample.1)
data$sample.2 <- paste0(data$cell.2, ": ", data$sample.2)

# Set colour gradient limits
limits <- c(0, 50, 90, 100)

# Plot
fig_2 <- ggplot(data, aes(x = sample.1, y = sample.2, fill = score)) +
  geom_tile(colour = "white", size = 0.3) +
  coord_equal() +
  theme(axis.ticks = element_blank(),
        panel.background = element_blank(),
        axis.text.x = element_text(angle = 90, hjust = 1, vjust = 0.5),
        legend.key.height = unit(1, "in")) +
  labs(x = NULL, y = NULL, fill = "Score") +
  scale_fill_gradientn(
    colours = c("white", "white", "#808080", "#1954a6"),
    limits = c(0, 100), values = rescale(limits))
```

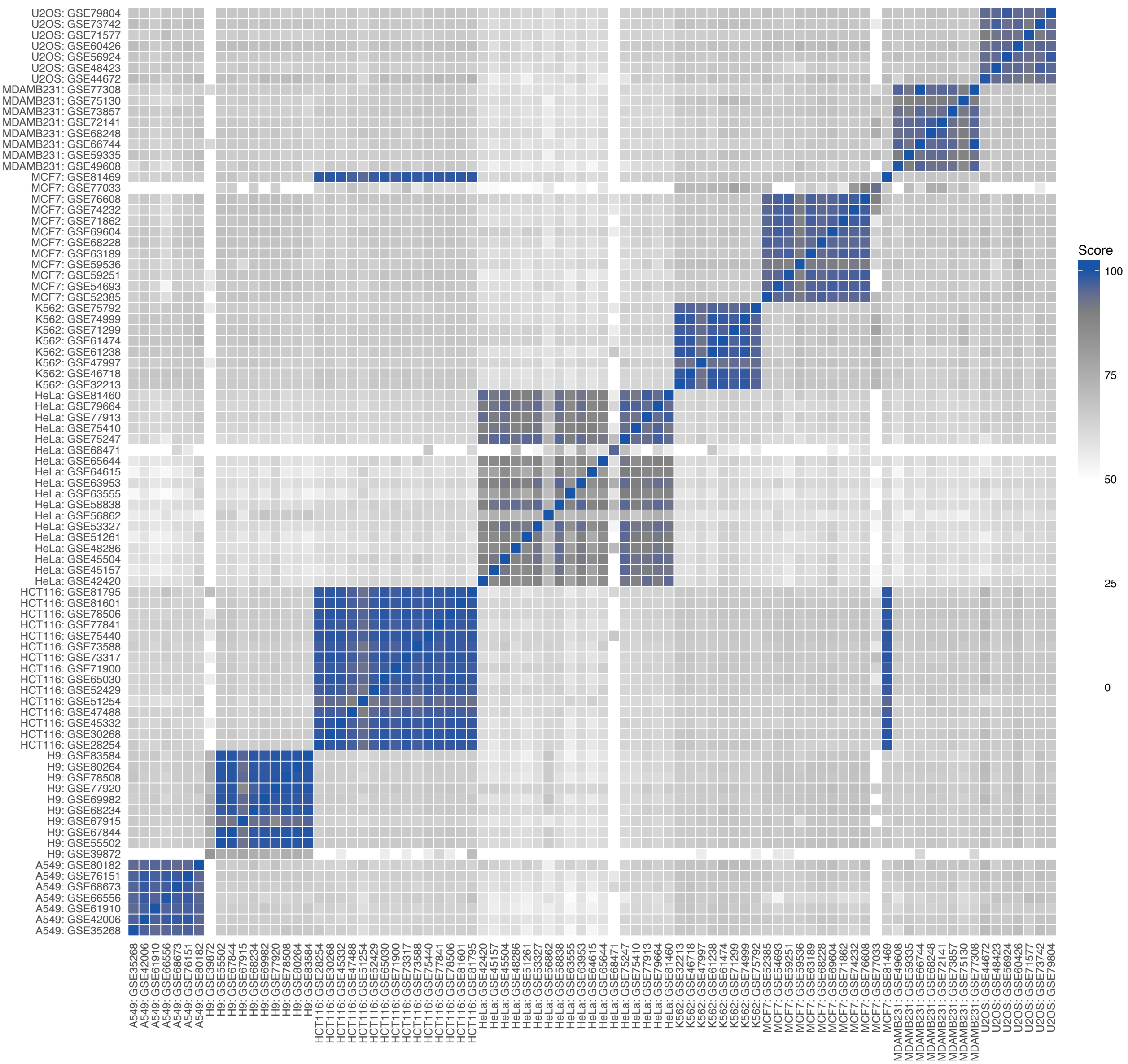
U2OS: GSE79804
U2OS: GSE73742
U2OS: GSE71577
U2OS: GSE60426
U2OS: GSE56924
U2OS: GSE48423
U2OS: GSE44672
MDAMB231: GSE77308
MDAMB231: GSE75130
MDAMB231: GSE73857
MDAMB231: GSE72141
MDAMB231: GSE68248
MDAMB231: GSE66744
MDAMB231: GSE59335
MDAMB231: GSE49608
MCF7: GSE81469
MCF7: GSE77033
MCF7: GSE76608
MCF7: GSE74232
MCF7: GSE71862
MCF7: GSE69604
MCF7: GSE68228
MCF7: GSE63189
MCF7: GSE59536
MCF7: GSE59251
MCF7: GSE54693
MCF7: GSE52385
K562: GSE75792
K562: GSE74999
K562: GSE71299
K562: GSE61474
K562: GSE61238
K562: GSE47997
K562: GSE46718
K562: GSE32213
HeLa: GSE81460
HeLa: GSE79664
HeLa: GSE77913
HeLa: GSE75410
HeLa: GSE75247
HeLa: GSE68471
HeLa: GSE65644
HeLa: GSE64615
HeLa: GSE63953
HeLa: GSE63555
HeLa: GSE58838
HeLa: GSE56862
HeLa: GSE53327
HeLa: GSE51261
HeLa: GSE48286
HeLa: GSE45504
HeLa: GSE45157
HeLa: GSE42420
HCT116: GSE81795
HCT116: GSE81601
HCT116: GSE78506
HCT116: GSE77841
HCT116: GSE75440
HCT116: GSE73588
HCT116: GSE73317
HCT116: GSE71900
HCT116: GSE65030
HCT116: GSE52429
HCT116: GSE51254
HCT116: GSE47488
HCT116: GSE45332
HCT116: GSE30268
HCT116: GSE28254
H9: GSE83584
H9: GSE80264
H9: GSE78508
H9: GSE77920
H9: GSE69982
H9: GSE68234
H9: GSE67915
H9: GSE67844
H9: GSE55502
H9: GSE39872
A549: GSE80182
A549: GSE76151
A549: GSE68673
A549: GSE66556
A549: GSE61910
A549: GSE42006
A549: GSE35268
A549: GSE35268
A549: GSE42006
A549: GSE61910
A549: GSE66556
A549: GSE68673
A549: GSE76151
A549: GSE80182
H9: GSE39872
H9: GSE55502
H9: GSE67844
H9: GSE67915
H9: GSE68234
H9: GSE69982
H9: GSE77920
H9: GSE78508
H9: GSE80264
H9: GSE83584
HCT116: GSE28254
HCT116: GSE30268
HCT116: GSE45332
HCT116: GSE47488
HCT116: GSE51254
HCT116: GSE52429
HCT116: GSE65030
HCT116: GSE71900
HCT116: GSE73317
HCT116: GSE73588
HCT116: GSE75440
HCT116: GSE77841
HCT116: GSE78506
HCT116: GSE81601
HCT116: GSE81795
HeLa: GSE42420
HeLa: GSE45157
HeLa: GSE45504
HeLa: GSE48286
HeLa: GSE51261
HeLa: GSE53327
HeLa: GSE56862
HeLa: GSE58838
HeLa: GSE63555
HeLa: GSE63953
HeLa: GSE64615
HeLa: GSE65644
HeLa: GSE68471
HeLa: GSE75247
HeLa: GSE75410
HeLa: GSE77913
HeLa: GSE79664
HeLa: GSE81460
K562: GSE32213
K562: GSE46718
K562: GSE47997
K562: GSE61238
K562: GSE61474
K562: GSE71299
K562: GSE74999
K562: GSE75792
MCF7: GSE52385
MCF7: GSE54693
MCF7: GSE59251
MCF7: GSE59536
MCF7: GSE63189
MCF7: GSE68228
MCF7: GSE69604
MCF7: GSE71862
MCF7: GSE74232
MCF7: GSE76608
MCF7: GSE77033
MCF7: GSE81469
MDAMB231: GSE49608
MDAMB231: GSE59335
MDAMB231: GSE66744
MDAMB231: GSE68248
MDAMB231: GSE72141
MDAMB231: GSE73857
MDAMB231: GSE75130
MDAMB231: GSE77308
U2OS: GSE44672
U2OS: GSE48423
U2OS: GSE56924
U2OS: GSE60426
U2OS: GSE71577
U2OS: GSE73742
U2OS: GSE79804
Score
100
75
50
25
0

Figure 3

Correlations of similarity scores versus the number of differentially expressed genes (A), the median fold change of differentially expressed genes (B), enrichment of KEGG Module functional units among differentially expressed genes (C), impact-specific similarity scores (D), and ANOVA for mean allelic ratios (E).

```
# [DEGs]
# Correlations per cell
corrs <- data.frame(cell             = character(),
                    rho.degs         = numeric(),
                    pval.degs        = numeric(),
                    rho.fc           = numeric(),
                    pval.fc          = numeric(),
                    stringsAsFactors = FALSE)

# Correlations for each cell (all degs)
for (cell in unique(data.degs$cell)) {

    # Subset for current cell line
    current <- data.degs[data.degs$cell == cell, ]

    # Correlations
    corr.degs <- cor.test(current$degs, current$score,
                          method = "pearson", use = "complete.obs")
    corr.fc <- cor.test(current$median.fc, current$score,
                        method = "pearson", use = "complete.obs")

    # Add results to dataframe
    corrs <- rbind(corrs, data.frame(cell      = cell,
                                     rho.degs  = round(corr.degs$estimate, 2),
                                     pval.degs = round(corr.degs$p.value, 3),
                                     rho.fc    = round(corr.fc$estimate, 2),
                                     pval.fc   = round(corr.fc$p.value, 3)))
}
corrs
```

```
##          cell rho.degs pval.degs rho.fc pval.fc
## cor      A549     0.59     0.598   0.52   0.649
## cor1       H9    -0.71     0.005  -0.61   0.021
## cor2   HCT116    -0.98     0.000  -0.99   0.000
## cor3     HeLa    -0.35     0.146   0.44   0.061
## cor4     K562    -0.12     0.824  -0.31   0.550
## cor5     MCF7     0.70     0.012  -0.84   0.001
## cor6 MDAMB231    -0.68     0.001  -0.58   0.006
## cor7     U2OS     0.71     0.498   0.76   0.452
```

```
# Merge with data
data.corrs <- merge(data.degs, corrs, by = "cell")

# Plot correlations: all DEGs
gg.degs.all <- ggplot(data.corrs[data.corrs$pval.degs  <= 0.01, ],
                 aes(x = degs, y = score, colour = cell, shape = cell)) +
    geom_point() +
    geom_smooth(method = "lm", se = FALSE) +
    theme_bw() +
```

```r
    theme(legend.position = "none",
          plot.margin = unit(c(40, 5, 5, 5), "pt")) +
    labs(x = "Total number of differentially expressed genes",
         y = "Similarity score") +
    scale_colour_manual(name = "",
                        values = c("#4ebfe4", "#e4bf4e", "#4e8ce4")) +
    scale_shape_manual(name = "", values = c(15, 17, 16))

# Plot correlations: fold change
gg.fc.all <- ggplot(data.corrs[data.corrs$pval.fc  <= 0.01, ],
                aes(x = median.fc, y = score, colour = cell, shape = cell)) +
    geom_point() +
    geom_smooth(method = "lm", se = FALSE) +
    theme_bw() +
    theme(legend.position = "none",
          plot.margin = unit(c(40, 5, 5, 5), "pt")) +
    labs(x = "Median fold change of differentially expressed genes",
         y = "Similarity score") +
    scale_colour_manual(name = "",
                        values = c("#e4bf4e", "#4ee499", "#4e8ce4")) +
    scale_shape_manual(name = "", values = c(17, 18, 16))
```

```r
# [Prognostic DEGs]
# Correlations per cell
corrs <- data.frame(cell             = character(),
                    rho.degs         = numeric(),
                    pval.degs        = numeric(),
                    rho.fc           = numeric(),
                    pval.fc          = numeric(),
                    stringsAsFactors = FALSE)

# Correlations for each cell (all degs)
for (cell in unique(data.degs.prog$cell)) {

    # Subset for current cell line
    current <- data.degs.prog[data.degs.prog$cell == cell, ]

    # Correlations
    corr.degs <- cor.test(current$n_prognostic_degs, current$score,
                          method = "pearson", use = "complete.obs")
    corr.fc <- cor.test(current$median_prognostic_fc, current$score,
                        method = "pearson", use = "complete.obs")

    # Add results to dataframe
    corrs <- rbind(corrs, data.frame(cell      = cell,
                                     rho.degs  = round(corr.degs$estimate, 2),
                                     pval.degs = round(corr.degs$p.value, 3),
                                     rho.fc    = round(corr.fc$estimate, 2),
                                     pval.fc   = round(corr.fc$p.value, 3)))
}
corrs
```

```
##          cell rho.degs pval.degs rho.fc pval.fc
## cor      A549     0.69     0.130   0.13   0.811
```

```
## cor1   HCT116    -0.98     0.000  -0.98   0.000
## cor2     HeLa    -0.19     0.227   0.32   0.049
## cor3     MCF7     0.05     0.748  -0.34   0.027
## cor4 MDAMB231    -0.69     0.000  -0.40   0.003
```

```
# Merge with data
data.corrs <- merge(data.degs.prog, corrs, by = "cell")

# Plot correlations: all DEGs
gg.degs.prog <- ggplot(data.corrs[data.corrs$pval.degs  <= 0.01, ],
                  aes(x = n_prognostic_degs, y = score, colour = cell,
                      shape = cell)) +
    geom_point() +
    geom_smooth(method = "lm", se = FALSE) +
    theme_bw() +
    theme(legend.position = "none",
          plot.margin = unit(c(40, 5, 5, 5), "pt")) +
    labs(x = "Total number of differentially expressed prognostic markers",
         y = "Similarity score") +
    scale_colour_manual(name = "", values = c("#e4bf4e", "#4e8ce4")) +
    scale_shape_manual(name = "", values = c(17, 16))

# Plot correlations: fold change
gg.fc.prog <- ggplot(data.corrs[data.corrs$pval.fc  <= 0.01, ],
                aes(x = median_prognostic_fc, y = score, colour = cell,
                    shape = cell)) +
    geom_point() +
    geom_smooth(method = "lm", se = FALSE) +
    theme_bw() +
    theme(legend.position = "none",
          plot.margin = unit(c(40, 5, 5, 5), "pt")) +
    labs(x = "Median fold change of differentially expressed prognostic markers",
         y = "Similarity score") +
    scale_colour_manual(name = "", values = c("#e4bf4e", "#4e8ce4")) +
    scale_shape_manual(name = "", values = c(17, 16))
```

```
# [SNV impacts]
# Remove self-comparisons and NA rows
data <- data.transc.snvs[data.transc.snvs$sample_1 !=
                         data.transc.snvs$sample_2, ]
data <- data[complete.cases(data), ]

# Factorise match/impact columns
data$match <- factor(data$match, levels = c("yes", "no"))
data$impact <- factor(data$impact,
                      levels = c("HIGH", "MODERATE", "LOW", "MODIFIER"))

# Merge
data <- merge(data, metadata, by.x = "sample_1", by.y = "GSE")
data <- merge(data, metadata, by.x = "sample_2", by.y = "GSE")

# Cell line-specific data
data <- data[data$cell.line.x == data$cell.line.y, ]

# Group per cell line, impact and match
```

```r
impact <- data %>% group_by(cell.line.x, impact, match) %>%
    summarise(count = n()) %>%
    mutate(total = sum(count))
impact
```

```
## # A tibble: 64 x 5
## # Groups:   cell.line.x, impact [32]
##    cell.line.x impact   match count total
##    <fct>       <fct>    <fct> <int> <int>
##  1 A549        HIGH     yes     528   550
##  2 A549        HIGH     no       22   550
##  3 A549        MODERATE yes   16429 17000
##  4 A549        MODERATE no      571 17000
##  5 A549        LOW      yes   27469 28498
##  6 A549        LOW      no     1029 28498
##  7 A549        MODIFIER yes   50128 51880
##  8 A549        MODIFIER no     1752 51880
##  9 H9          HIGH     yes    2610  2653
## 10 H9          HIGH     no       43  2653
## # ... with 54 more rows
```

```r
# Calculate score per cell line impact
impact <- impact[impact$match == "yes", ]
impact$score <- (impact$count + 1) / (impact$total + 5) * 100

# Plot impact distributions
gg.impact <- ggplot(impact, aes(x = cell.line.x, y = score, colour = impact,
                               group = impact)) +
    geom_point(size = 3, aes(shape = impact)) +
    theme_bw() +
    labs(x = NULL, y = "Similarity score") +
    theme(panel.grid.minor.y = element_blank(),
          legend.position = "none", plot.margin = unit(c(40,5,5,5), "pt")) +
    scale_colour_manual(name = "",
                        values = c("#a6c6f2", "#4e8ce4", "#1954a6", "#0d2d59"))
```

```r
# [eSNP-Karyotyping]

# Get eSNP-Karyotyping data
data <- data.esnp

# Find treatment and response columns and rename then
names(data)[grep("cell.line", names(data))] <- "treatment"
names(data)[grep("mean_ar", names(data))] <- "response"

# Remove rows where treatment is missing
data <- data[data$treatment != "", ]

# Make into factors
data$treatment <- factor(data$treatment, levels = sort(unique(data$treatment)))

# Perform ANOVA
model <- lm(response ~ treatment, data = data)
aov <- aov(model)
```

```r
# Perform Tukey"s Honest Significant Difference test
tukey.test <- TukeyHSD(aov, conf.level = 0.99)

# Coerce into data frame an get treatment groups
tukey <- as.data.frame(tukey.test$treatment)
tukey$treatment <- row.names(tukey)
names(tukey) <- c("diff", "lwr", "upr", "p.adj", "treatment")

# Separate treatment groups into two columns
tukey <- separate(data = tukey, col = treatment, into = c("treat_1", "treat_2"), sep = "-")
tukey <- arrange(tukey, desc(treat_2), desc(treat_1))
tukey$treatment <- paste(tukey$treat_1, tukey$treat_2, sep = " - ")
tukey$treatment <- factor(tukey$treatment, levels = unique(tukey$treatment))

# Add colour groups based on significance
tukey$colour.groups <- "A"
tukey[tukey$p.adj <= 0.01, "colour.groups"] <- "A"
tukey[tukey$p.adj > 0.01, "colour.groups"] <- "B"

# Proportional width of boxplots
box.width = length(unique(data$treatment)) * 0.09

# Find groups with significant differences
levels <- tukey.test[["treatment"]][,4]
labels <- data.frame(multcompLetters(levels)["Letters"])
labels$treatment <- row.names(labels)
labels <- arrange(labels, treatment)

# Separate groups where applicable
labels <- separate(labels, col = "Letters", into = c("group_1", "group_2"), sep = 1)
labels[labels$group_2 == "", "group_2"] <- labels[labels$group_2 == "",
                                                  "group_1"]

# Calculate quantiles for fill colours of boxplots
quantiles <- data %>% group_by(treatment) %>%
    summarise(y1 = quantile(response, probs = 0.25),
              y2 = quantile(response, probs = 0.75))

# Merge with labels
shading <- merge(labels, quantiles, by = "treatment")

# Find coordinates
shading$x1 <- as.numeric(row.names(shading)) - box.width / 2
shading$x2 <- as.numeric(row.names(shading)) + box.width / 2

# Merge with data
data <- merge(data, shading[c(1,2)], by = "treatment")

# Triangles
group.1 <- c()
group.2 <- c()
triangle <- c()
t1.x <- c()
```

```r
t1.y <- c()
t2.x =  c()
t2.y <- c()
for (n in c(1:nrow(shading))) {
    group.1 <- c(group.1, rep(shading[n, "group_1"], 3))
    group.2 <- c(group.2, rep(shading[n, "group_2"], 3))
    triangle <- c(triangle, n, n, n)
    t1.x <- c(t1.x, shading[n, "x1"], shading[n, "x1"], shading[n, "x2"])
    t1.y <- c(t1.y, shading[n, "y1"], shading[n, "y2"], shading[n, "y2"])
    t2.x <- c(t2.x, shading[n, "x1"], shading[n, "x2"], shading[n, "x2"])
    t2.y <- c(t2.y, shading[n, "y1"], shading[n, "y1"], shading[n, "y2"])
}
triangle.1 <- data.frame(group = group.1, triangle = triangle, x = t1.x, y = t1.y)
triangle.2 <- data.frame(group = group.2, triangle = triangle, x = t2.x, y = t2.y)

# Colour palette
palette <- c("#4e8ce4", "#a6c6f2", "#a6a6a6", "#8c8c8c")

# Change underscores to spaces in treatment names
data$treatment <- gsub("_", " ", data$treatment)

# Boxplot
gg.box <- ggplot(data, aes(x = treatment, y = response, fill = group_1)) +
    geom_boxplot(outlier.shape = NA, colour = "#4d4d4d", width = box.width) +
    geom_polygon(data = triangle.2, aes(x = x, y = y, group = triangle, fill = group)) +
    geom_boxplot(outlier.shape = NA, colour = "#4d4d4d", width = box.width,
                 aes(fill = NA)) +
    stat_boxplot(data = data, geom = "errorbar", width = 0.15,
                 aes(x = treatment, y = response), colour = "#4d4d4d") +
    geom_point(position = position_jitter(w = 0.15, h = 0),
               stroke = 0.5, alpha = 0.75,  size = 1, colour = "#4d4d4d") +
    theme_bw() +
    theme(legend.position = "None", plot.margin = unit(c(40,5,5,5), "pt")) +
    labs(x = NULL, y = "Mean allelic ratio") +
    scale_fill_manual(values = palette)
```

```r
# Add figure labels
gg.degs.all <-  cowplot::plot_grid(gg.degs.all,   labels = "A", label_size = 20)
gg.fc.all <-    cowplot::plot_grid(gg.fc.all,     labels = "B", label_size = 20)
gg.degs.prog <- cowplot::plot_grid(gg.degs.prog,  labels = "C", label_size = 20)
gg.fc.prog <-   cowplot::plot_grid(gg.fc.prog,    labels = "D", label_size = 20)
gg.impact <-    cowplot::plot_grid(gg.impact,     labels = "E", label_size = 20)
gg.box <-       cowplot::plot_grid(gg.box,        labels = "F", label_size = 20)

# Arrange in grid
layout <- c(1, 2,
            3, 4,
            5, 6)
fig_3 <- grid.arrange(arrangeGrob(gg.degs.all, gg.fc.all, nrow = 1),
                      arrangeGrob(gg.degs.prog, gg.fc.prog, nrow = 1),
                      arrangeGrob(gg.impact, gg.box, nrow = 1),
                      nrow = 3)
```

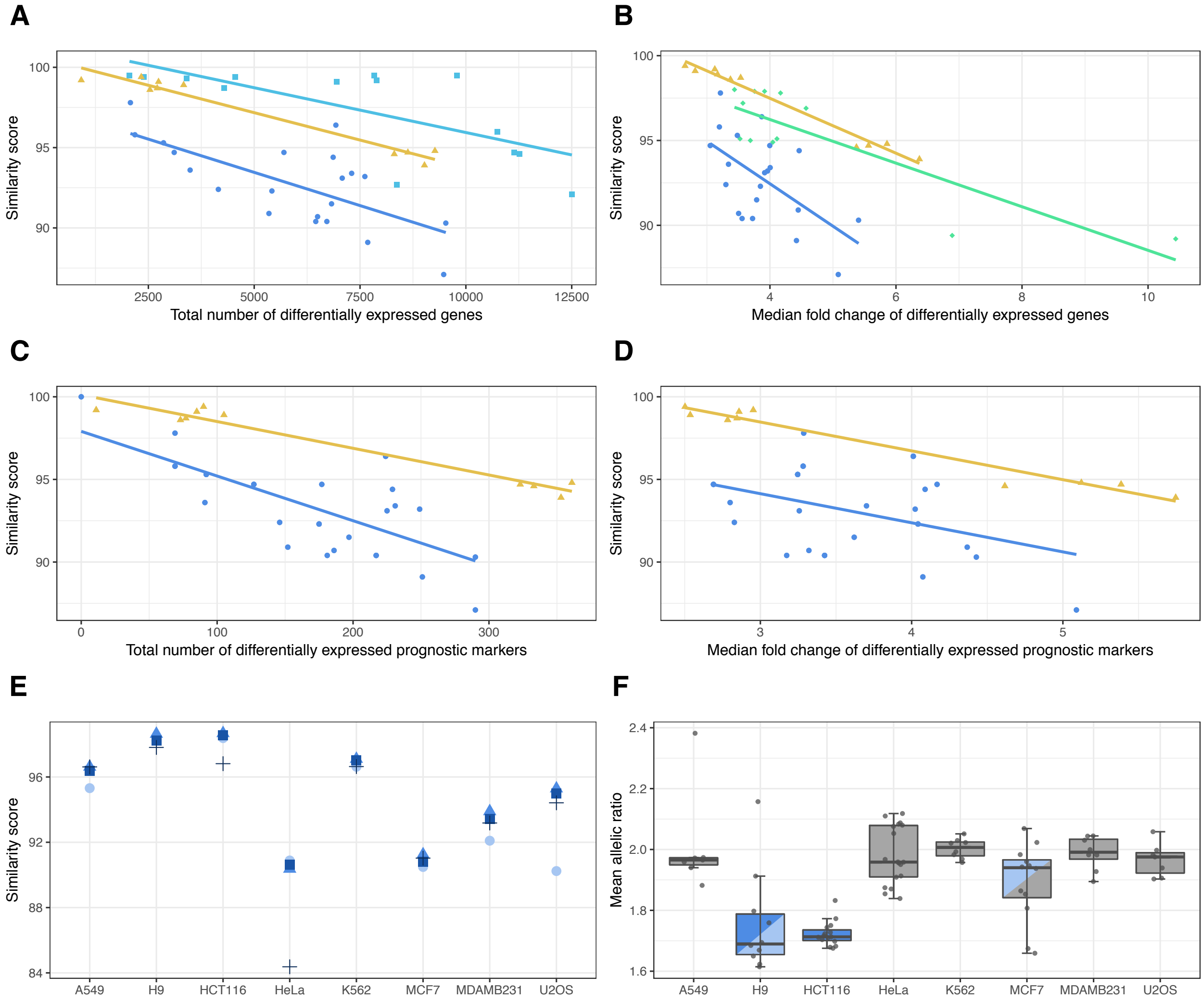

A
Similarity score
Total number of differentially expressed genes
B
Similarity score
Median fold change of differentially expressed genes
C
Similarity score
Total number of differentially expressed prognostic markers
D
Similarity score
Median fold change of differentially expressed prognostic markers
E
Similarity score
A549
H9
HCT116
HeLa
K562
MCF7
MDAMB231
U2OS
F
Mean allelic ratio
A549
H9
HCT116
HeLa
K562
MCF7
MDAMB231
U2OS

Supplementary figures

Supplementary figure 1

COSMIC concordance histograms for each analysed cell line.

```
# Get data from above
data <- data.cosmic

# Get unique cell lines
cells <- unique(data$cell.line)

# Loop over cell lines
plot_list <- list()
labels <- c("A", "B", "C", "D", "E", "F", "G", "H")
n <- 1
for ( cell in cells ) {

  # Subset data for current cell line
  data.cell <- data[data$cell.line == cell, ]

  # Replace NaN with zero
  data.cell[is.na(data.cell$cosmic.concordance), "cosmic.concordance"] <- 0

  # Colour groups
  data.cell[data.cell$cosmic.matches  >=  50, "group"] <- "1"
  data.cell[data.cell$cosmic.matches < 50, "group"] <- "2"
  data.cell[data.cell$cosmic.matches <= 10, "group"] <- "3"
  data.cell$group <- factor(data.cell$group, levels = c("1", "2", "3"))

  # Plot
  gg <- ggplot(data.cell, aes(x = GSE, y = cosmic.concordance)) +
      geom_bar(stat = "identity", position = "dodge", colour = "black", size = 0.3,
               aes(fill = group)) +
    scale_fill_manual(values = c("#0d2d59", "#1954a6", "#4e8ce4")) +
    geom_hline(aes(yintercept = 90), colour = "black", linetype = "dotted") +
    theme_bw() +
    theme(axis.text.y = element_text(size = 8), axis.title = element_text(size = 17.5),
          plot.title = element_text(size = 17.5, hjust = 0.5),
          axis.text.x = element_text(size = 12, angle = 90, vjust = 0.5),
          axis.ticks.x = element_blank(), legend.position = "none",
          plot.margin = unit(c(40, 5, 5, 5), "pt")) +
    labs(x = NULL, y = "Concordance (%)", title = cell) +
    geom_text(data = data.cell, aes(label = paste0(cosmic.matches, "/",
                                                   cosmic.calls)),
              position = position_dodge(width = 0.9), vjust = -0.5, size = 2) +
    geom_text(data = data.cell, aes(label = paste0(cosmic.concordance, "%")),
              position = position_dodge(width = 0.9), vjust = 1.6, size = 2.5,
              colour = "white") +
    ylim(0, 105) +
      annotation_custom(grob = textGrob(label = labels[n], hjust = 0, vjust = -0.625,
                                        gp = gpar(cex = 2.5)),
                        ymin = Inf, ymax = Inf, xmin = -Inf, xmax = -Inf)
```

```
    gg <- ggplot_gtable(ggplot_build(gg))
    gg$layout$clip[gg$layout$name == "panel"] <- "off"

    # Add to plot list
    plot_list[[n]] <- gg
    n <- n + 1
}

sfig_1 <- grid.arrange(plot_list[[1]],
                  plot_list[[2]],
                  plot_list[[3]],
                  plot_list[[4]],
                  plot_list[[5]],
                  plot_list[[6]],
                  plot_list[[7]],
                  plot_list[[8]], ncol = 3)
```

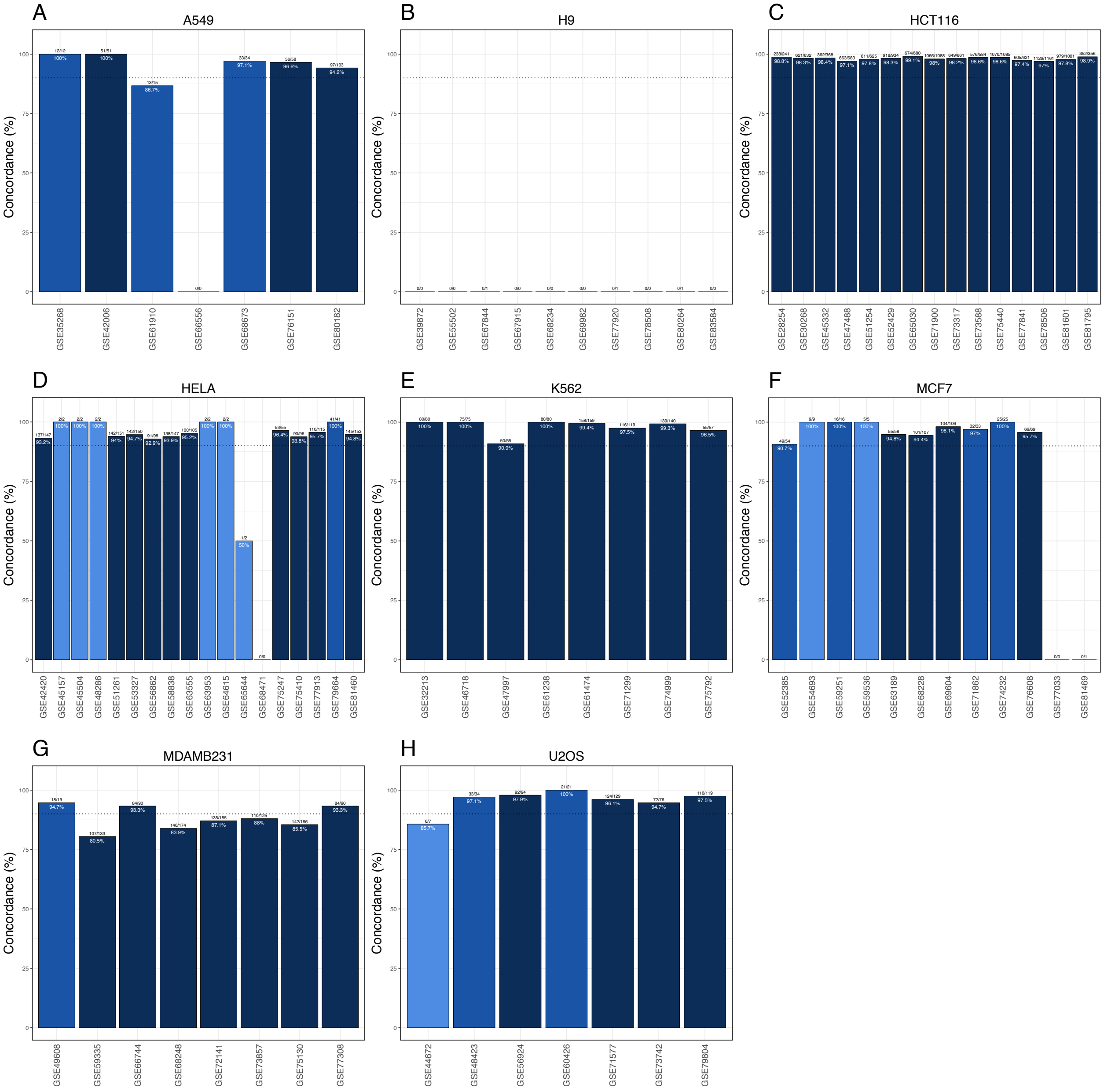
A
A549
Concordance (%)
GSE35268
GSE42006
GSE61910
GSE66556
GSE68673
GSE76151
GSE80182
B
H9
GSE39872
GSE55502
GSE67844
GSE67915
GSE68234
GSE69982
GSE77920
GSE78508
GSE80264
GSE83584
C
HCT116
GSE28254
GSE30268
GSE45332
GSE47488
GSE51254
GSE52429
GSE65030
GSE71900
GSE73317
GSE73588
GSE75440
GSE77841
GSE78506
GSE81601
GSE81795
D
HELA
GSE42420
GSE45157
GSE45504
GSE48286
GSE51261
GSE53327
GSE56862
GSE58838
GSE63555
GSE63953
GSE64615
GSE65644
GSE68471
GSE75247
GSE75410
GSE77913
GSE79664
GSE81460
E
K562
GSE32213
GSE46718
GSE47997
GSE61238
GSE61474
GSE71299
GSE74999
GSE75792
F
MCF7
GSE52385
GSE54693
GSE59251
GSE59536
GSE63189
GSE66228
GSE69604
GSE71862
GSE74232
GSE76608
GSE77033
GSE81469
G
MDAMB231
GSE49608
GSE59335
GSE66744
GSE68248
GSE72141
GSE73857
GSE75130
GSE77308
H
U2OS
GSE44672
GSE48423
GSE56924
GSE60426
GSE71577
GSE73742
GSE79804

Supplementary figure 2

Global impact distributions for both COSMIC and transcriptome-wide data.

```
# Get COSMIC SNV data
data <- data.cosmic.snvs

# Factorise COSMIC match/impact columns
data$match.cosmic <- factor(data$match.cosmic, levels = c("yes", "no"))
data$impact <- factor(data$impact, levels = c("HIGH", "MODERATE", "LOW",
                                               "MODIFIER"))

impact <- data %>% group_by(match.cosmic, impact) %>% summarise(count = n()) %>%
  mutate(prop = round(count/sum(count) * 100, 1))

# Plot impact
gg <- ggplot(impact, aes(x = impact, y = prop, fill = match.cosmic)) +
  geom_bar(stat = "identity", position = "dodge", color = "black", size = 0.3) +
  theme_bw() +
  labs(x = "\nImpact category", y = "Proportion of COSMIC SNVs in category (%)") +
  geom_text(aes(label = count), position = position_dodge(width = 0.9), vjust = -0.5,
            size = 2.5) +
  geom_text(data = impact[impact$prop > 5, ], aes(label = paste0(prop, " %")),
            position = position_dodge(width = 0.9), vjust = 1.6, size = 3.5,
            colour = "white") +
  geom_text(data = impact[impact$prop < 5, ], aes(y = 0, label = paste0(prop, " %")),
            position = position_dodge(width = 0.9), vjust = 1.6, size = 3.5,
            colour = "#4d4d4d") +
  scale_fill_manual(values = c("#0d2d59", "#1954a6")) +
  theme(legend.position = "none", axis.text = element_text(size = 14),
        panel.grid.major.x = element_blank(), axis.title = element_text(size = 17.5),
        plot.margin = unit(c(40, 5, 5, 5), "pt")) +
        annotation_custom(grob = textGrob(label = "A", hjust = 0, vjust = -0.625,
                                          gp = gpar(cex = 2.5)),
                          ymin = Inf, ymax = Inf, xmin = -Inf, xmax = -Inf) +
    ylim(0, 75)

gg <- ggplot_gtable(ggplot_build(gg))
gg$layout$clip[gg$layout$name == "panel"] <- "off"

# Get transcriptome SNV data
data <- data.transc.snvs

# Remove self-comparisons and NA rows (if present)
data <- data[data$sample_1 != data$sample_2, ]
data <- data[complete.cases(data), ]

# Factorise match/impact columns
data$match <- factor(data$match, levels = c("yes", "no"))
data$impact <- factor(data$impact, levels = c("HIGH", "MODERATE", "LOW",
                                               "MODIFIER"))

# Merge with metadata
data <- merge(data, metadata, by.x = "sample_1", by.y = "GSE")
```

```r
data <- merge(data, metadata, by.x = "sample_2", by.y = "GSE")

# Cell line-specific data
data <- data[data$cell.line.x == data$cell.line.y, ]

# Impact stats (transcriptome)
impact <- data %>% group_by(match, impact) %>%
  summarise(count = n()) %>% mutate(prop = round(count/sum(count) * 100, 1))
impact <- impact[impact$match == "yes" | impact$match == "no", ]
impact <- impact[-9, ]

# Plot impact
hh <- ggplot(impact, aes(x = impact, y = prop, fill = match)) +
  geom_bar(stat = "identity", position = "dodge", color = "black", size = 0.3) +
  theme_bw() +
  labs(x = "\nImpact category",
       y = "Proportion of transcriptome-wide SNVs in category (%)") +
    ylim(0, 75) +
  geom_text(aes(label = count), position = position_dodge(width = 0.9), vjust = -0.5,
            size = 2.5) +
  geom_text(data = impact[impact$prop > 5, ], aes(label = paste0(prop, " %")),
            position = position_dodge(width = 0.9), vjust = 1.6, size = 3.5,
            colour = "white") +
  geom_text(data = impact[impact$prop < 5, ], aes(y = 0, label = paste0(prop, " %")),
            position = position_dodge(width = 0.9), vjust = 1.6, size = 3.5,
            colour = "#4d4d4d") +
  scale_fill_manual(values = c("#0d2d59", "#1954a6")) +
  theme(legend.position = "none", axis.text = element_text(size = 14),
        panel.grid.major.x = element_blank(), axis.title = element_text(size = 17.5),
        plot.margin = unit(c(40, 5, 5, 5), "pt")) +
        annotation_custom(grob = textGrob(label = "B", hjust = 0, vjust = -0.625,
                                          gp = gpar(cex = 2.5)),
                          ymin = Inf, ymax = Inf, xmin = -Inf, xmax = -Inf)

hh <- ggplot_gtable(ggplot_build(hh))
hh$layout$clip[hh$layout$name == "panel"] <- "off"

sfig_2 <- grid.arrange(gg, hh, ncol = 2)
```

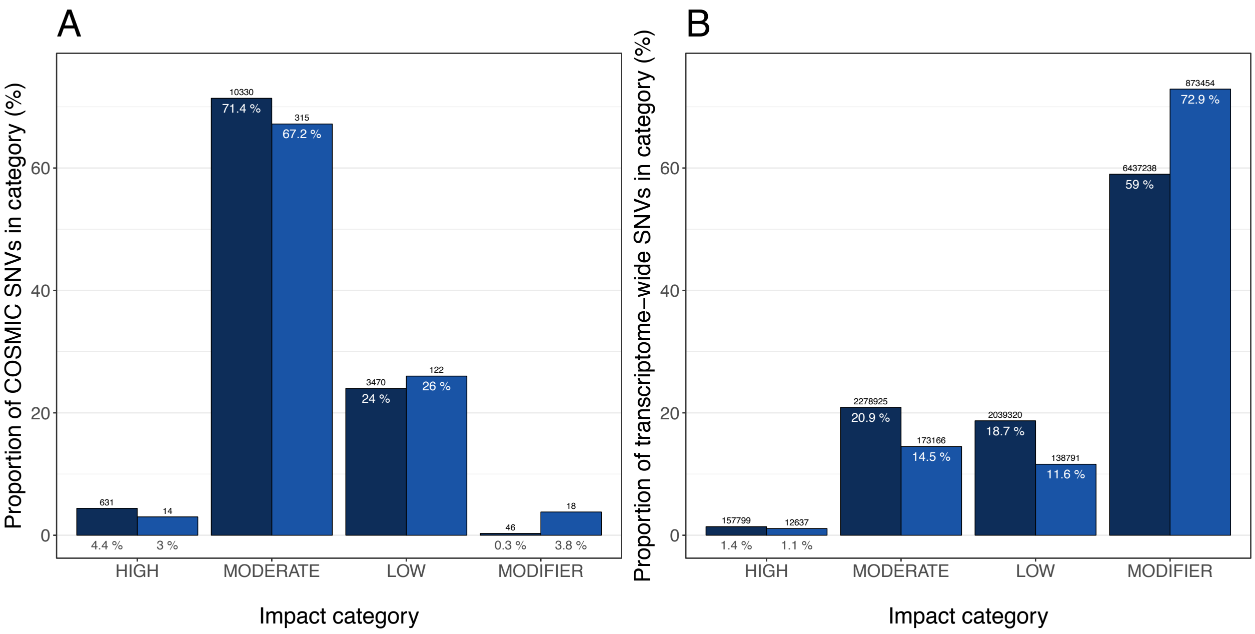
A
Proportion of COSMIC SNVs in category (%)
Impact category
HIGH
MODERATE
LOW
MODIFIER
631
14
10330
315
3470
122
46
18
4.4 %
3 %
71.4 %
67.2 %
24 %
26 %
0.3 %
3.8 %
B
Proportion of transcriptome−wide SNVs in category (%)
157799
12637
2278925
173166
2039320
138791
6437238
873454
1.4 %
1.1 %
20.9 %
14.5 %
18.7 %
11.6 %
59 %
72.9 %

Supplementary figure 3

The distribution of SNV zygosities for COSMIC variants.

```
# Get data
data <- data.cosmic.snvs

# Zygosity
data[data$cosmic_1 == data$cosmic_2, "cosmic.zyg"] <- "Homozygous"
data[data$cosmic_1 != data$cosmic_2, "cosmic.zyg"] <- "Heterozygous"
data[data$sample_1 == data$sample_2, "sample.zyg"] <- "Homozygous"
data[data$sample_1 != data$sample_2, "sample.zyg"] <- "Heterozygous"
data.hom <- data[data$sample.zyg == "Homozygous", ]

# Zygosity stats
zyg <- data %>% group_by(match.cosmic, sample.zyg) %>% summarise(count = n()) %>%
  mutate(prop = round(count/sum(count) * 100, 1))

# Plot zygosity
sfig_3 <- ggplot(zyg, aes(x = sample.zyg, y = prop, fill = match.cosmic)) +
  geom_bar(stat = "identity", position = "dodge", color = "black", size = 0.3) +
  theme_bw() +
  labs(x = "\nGenotype category", y = "Proportion of COSMIC SNVs in category (%)") +
  geom_text(aes(label = count), position = position_dodge(width = 0.9), vjust = -0.5,
            size = 2.5) +
  geom_text(aes(label = paste0(prop, " %")), position = position_dodge(width = 0.9),
            vjust = 1.6, size = 3.5, colour = "white") +
  scale_fill_manual(values = c("#0d2d59", "#1954a6")) +
  theme(legend.position = "none", axis.text = element_text(size = 14),
        panel.grid.major.x = element_blank(), axis.title = element_text(size = 17.5))
```

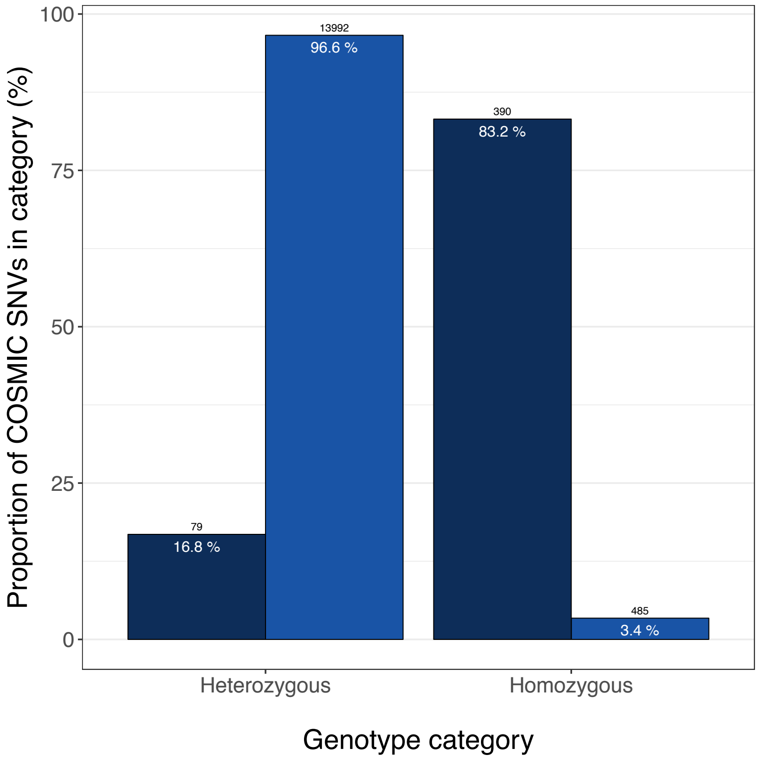

Supplementary figure 4

Transcriptome-wide concordance histograms for each analysed cell line.

```r
# Get transcriptome stats
data <- data.transc

# Unique cells
cells <- unique(data$cell.1)

# Loop over cell lines
labels <- c("A", "B", "C", "D", "E", "F", "G", "H")
n <- 1
plot_list <- list()
for ( cell in cells ) {

    # Subset data for current cell line
    data.cell <- data[data$cell.1 == data$cell.2 & data$cell.1 == cell, ]

    # Median concordance
    data.cell <- data.cell %>% group_by(sample.1) %>%
        summarise(median.conc = round(median(concordance, na.rm = TRUE), 1),
                  median.calls = round(median(calls, na.rm = TRUE), 0),
                  median.matches = round(median(matches, na.rm = TRUE), 0))

    # Colour groups
    data.cell[data.cell$median.matches  >=  50, "group"] <- "1"
    data.cell[data.cell$median.matches < 50, "group"] <- "2"
    data.cell[data.cell$median.matches <= 10, "group"] <- "3"
    data.cell$group <- factor(data.cell$group, levels = c("1", "2", "3"))

    # Plot
    gg <- ggplot(data.cell, aes(x = sample.1, y = median.conc)) +
    geom_bar(stat = "identity", position = "dodge", colour = "black", size = 0.3,
             aes(fill = group)) +
    scale_fill_manual(values = c("#0d2d59", "#1954a6", "#4e8ce4")) +
    geom_hline(aes(yintercept = 90), colour = "black", linetype = "dotted") +
    theme_bw() +
    theme(axis.text.y = element_text(size = 8), axis.title = element_text(size = 17.5),
          plot.title = element_text(size = 17.5, hjust = 0.5),
          axis.text.x = element_text(size = 12, angle = 90, vjust = 0.5),
          axis.ticks.x = element_blank(), legend.position = "none",
          plot.margin = unit(c(40, 5, 5, 5), "pt")) +
    labs(x = NULL, y = "Median concordance (%)", title = cell) +
    geom_text(data = data.cell, aes(label = median.calls),
              position = position_dodge(width = 0.9), vjust = -0.5, size = 2) +
    geom_text(data = data.cell, aes(label = paste0(median.conc, "%")),
              position = position_dodge(width = 0.9), vjust = 1.6, size = 2.5,
              colour = "white") +
    ylim(0, 105) +
        annotation_custom(grob = textGrob(label = labels[n], hjust = 0, vjust = -0.625,
                                          gp = gpar(cex = 2.5)),
                          ymin = Inf, ymax = Inf, xmin = -Inf, xmax = -Inf)
```

```
    gg <- ggplot_gtable(ggplot_build(gg))
    gg$layout$clip[gg$layout$name == "panel"] <- "off"

    plot_list[[n]] <- gg
    n <- n + 1
}


# Save
sfig_4 <- grid.arrange(plot_list[[1]],
                  plot_list[[2]],
                  plot_list[[3]],
                  plot_list[[4]],
                  plot_list[[5]],
                  plot_list[[6]],
                  plot_list[[7]],
                  plot_list[[8]], ncol = 3)
```

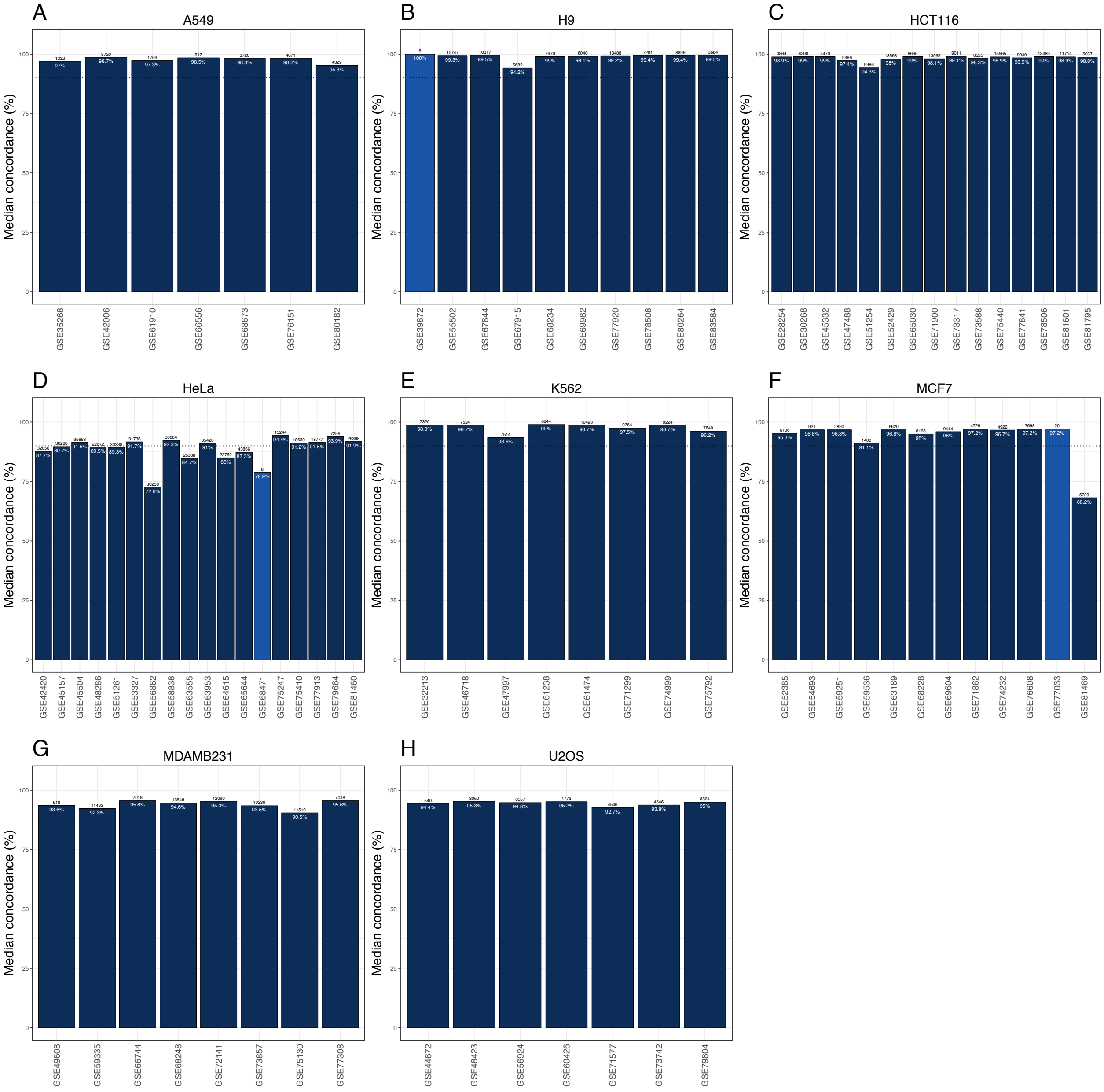
A
A549
Median concordance (%)
GSE35268
GSE42006
GSE61910
GSE66556
GSE68673
GSE76151
GSE80182
B
H9
GSE39872
GSE55502
GSE67844
GSE67915
GSE68234
GSE69982
GSE77920
GSE78508
GSE80264
GSE83584
C
HCT116
GSE28254
GSE30268
GSE45332
GSE47488
GSE51254
GSE52429
GSE65030
GSE71900
GSE73317
GSE73588
GSE75440
GSE77841
GSE78506
GSE81601
GSE81795
D
HeLa
GSE42420
GSE45157
GSE45504
GSE48286
GSE51261
GSE53327
GSE56862
GSE58838
GSE63555
GSE63953
GSE64615
GSE65644
GSE68471
GSE75247
GSE75410
GSE77913
GSE79664
GSE81460
E
K562
GSE32213
GSE46718
GSE47997
GSE61238
GSE61474
GSE71299
GSE74999
GSE75792
F
MCF7
GSE52385
GSE54693
GSE59251
GSE59536
GSE63189
GSE66228
GSE69604
GSE71862
GSE74232
GSE76608
GSE77033
GSE81469
G
MDAMB231
GSE49608
GSE59335
GSE66744
GSE68248
GSE72141
GSE73857
GSE75130
GSE77308
H
U2OS
GSE44672
GSE48423
GSE56924
GSE60426
GSE71577
GSE73742
GSE79804

Supplemetary figures 5 to 12

Individual transcriptome-wide heatmaps per studied cell line.

```
# Get transcriptome data
data.all <- data.transc

# Cells, sizes and sfigure numbering
all_cells <- unique(data.all$cell.1)
sizes <- c(5, 7, 8.5, 11, 6, 7, 6, 5.5)
sfigs <- c(5:12)

# Plot heatmap for each cell line
for (n in 1:length(all_cells)) {

    # Current cell line, size and sfig number
    cell <- all_cells[n]
    size <- sizes[n]
    sfig <- sfigs[n]

    # Subset for current cell line
    data <- data.all[data.all$cell.1 == cell &
                     data.all$cell.2 == cell, ]

    # Set colour gradient limits
    limits <- c(0, 50, 90, 100)

    # Plot
    gg <- ggplot(data, aes(x = sample.1, y = sample.2, fill = score)) +
        geom_tile(colour = "white", size = 0.3) +
        geom_text(colour = "white", size = 3, aes(
            label = paste0(score, "\n(", calls, ")"))) +
        coord_equal() +
        theme(axis.ticks = element_blank(),
              panel.background = element_blank(),
              axis.text.x = element_text(angle = 90, hjust = 1, vjust = 0.5),
              legend.position = "none",
              plot.title = element_text(hjust = 0.5)) +
        labs(x = NULL, y = NULL, title = cell) +
        scale_fill_gradientn(
            colours = c("white", "white", "#808080", "#1954a6"),
            limits = c(0, 100), values = rescale(limits))

    grid.arrange(gg)
    ggsave(paste0("../../images/sfigure_", as.character(sfig), ".png"), gg,
           dpi = 300, width = size, height = size)
}
```

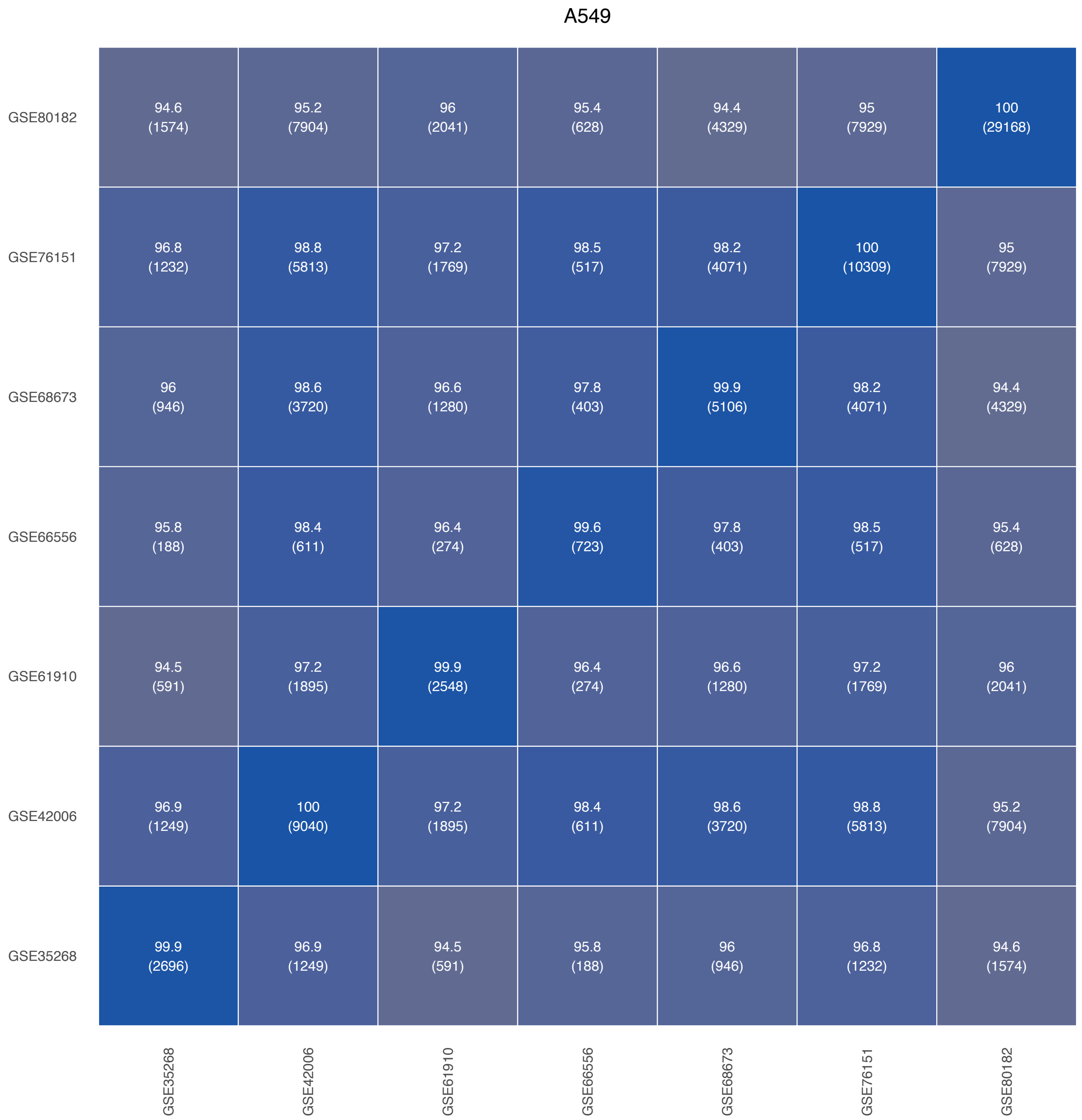
A549
GSE80182
94.6 (1574)
95.2 (7904)
96 (2041)
95.4 (628)
94.4 (4329)
95 (7929)
100 (29168)
GSE76151
96.8 (1232)
98.8 (5813)
97.2 (1769)
98.5 (517)
98.2 (4071)
100 (10309)
95 (7929)
GSE68673
96 (946)
98.6 (3720)
96.6 (1280)
97.8 (403)
99.9 (5106)
98.2 (4071)
94.4 (4329)
GSE66556
95.8 (188)
98.4 (611)
96.4 (274)
99.6 (723)
97.8 (403)
98.5 (517)
95.4 (628)
GSE61910
94.5 (591)
97.2 (1895)
99.9 (2548)
96.4 (274)
96.6 (1280)
97.2 (1769)
96 (2041)
GSE42006
96.9 (1249)
100 (9040)
97.2 (1895)
98.4 (611)
98.6 (3720)
98.8 (5813)
95.2 (7904)
GSE35268
99.9 (2696)
96.9 (1249)
94.5 (591)
95.8 (188)
96 (946)
96.8 (1232)
94.6 (1574)
GSE35268
GSE42006
GSE61910
GSE66556
GSE68673
GSE76151
GSE80182

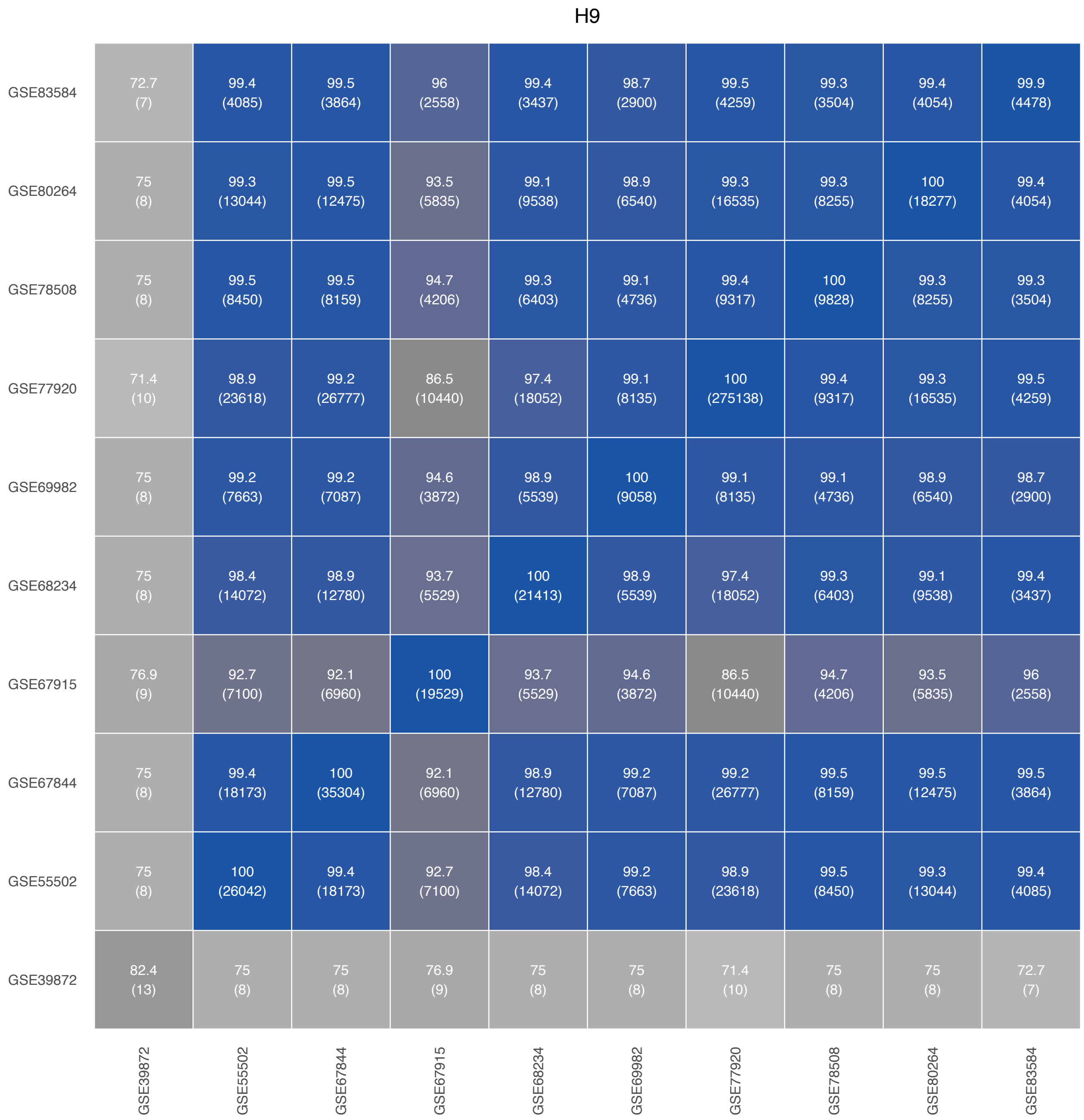
H9
GSE83584
72.7 (7)
99.4 (4085)
99.5 (3864)
96 (2558)
99.4 (3437)
98.7 (2900)
99.5 (4259)
99.3 (3504)
99.4 (4054)
99.9 (4478)
GSE80264
75 (8)
99.3 (13044)
99.5 (12475)
93.5 (5835)
99.1 (9538)
98.9 (6540)
99.3 (16535)
99.3 (8255)
100 (18277)
99.4 (4054)
GSE78508
75 (8)
99.5 (8450)
99.5 (8159)
94.7 (4206)
99.3 (6403)
99.1 (4736)
99.4 (9317)
100 (9828)
99.3 (8255)
99.3 (3504)
GSE77920
71.4 (10)
98.9 (23618)
99.2 (26777)
86.5 (10440)
97.4 (18052)
99.1 (8135)
100 (275138)
99.4 (9317)
99.3 (16535)
99.5 (4259)
GSE69982
75 (8)
99.2 (7663)
99.2 (7087)
94.6 (3872)
98.9 (5539)
100 (9058)
99.1 (8135)
99.1 (4736)
98.9 (6540)
98.7 (2900)
GSE68234
75 (8)
98.4 (14072)
98.9 (12780)
93.7 (5529)
100 (21413)
98.9 (5539)
97.4 (18052)
99.3 (6403)
99.1 (9538)
99.4 (3437)
GSE67915
76.9 (9)
92.7 (7100)
92.1 (6960)
100 (19529)
93.7 (5529)
94.6 (3872)
86.5 (10440)
94.7 (4206)
93.5 (5835)
96 (2558)
GSE67844
75 (8)
99.4 (18173)
100 (35304)
92.1 (6960)
98.9 (12780)
99.2 (7087)
99.2 (26777)
99.5 (8159)
99.5 (12475)
99.5 (3864)
GSE55502
75 (8)
100 (26042)
99.4 (18173)
92.7 (7100)
98.4 (14072)
99.2 (7663)
98.9 (23618)
99.5 (8450)
99.3 (13044)
99.4 (4085)
GSE39872
82.4 (13)
75 (8)
75 (8)
76.9 (9)
75 (8)
75 (8)
71.4 (10)
75 (8)
75 (8)
72.7 (7)
GSE39872
GSE55502
GSE67844
GSE67915
GSE68234
GSE69982
GSE77920
GSE78508
GSE80264
GSE83584

HCT116

	GSE28254	GSE30268	GSE45332	GSE47488	GSE51254	GSE52429	GSE65030	GSE71900	GSE73317	GSE73588	GSE75440	GSE77841	GSE78506	GSE81601	GSE81795
GSE81795	98.6 (2484)	99 (4453)	98.7 (2745)	96.9 (4570)	93 (4158)	97.6 (5612)	98.9 (5107)	97.7 (5418)	99 (5025)	98.1 (4056)	99.1 (5527)	98.5 (5037)	99.2 (5490)	98.9 (5140)	99.9 (5915)
GSE81601	98.9 (3991)	99.1 (9029)	99.1 (5125)	97.4 (14101)	95.1 (13230)	98.4 (18458)	99.1 (9974)	98.4 (18508)	99.1 (10353)	98.4 (11714)	98.6 (22922)	98.5 (11275)	98.9 (20107)	100 (32465)	98.9 (5140)
GSE78506	98.9 (4103)	99.4 (10097)	99.1 (5332)	97.9 (15733)	95.7 (14829)	98.5 (21582)	99.3 (11364)	98.5 (22704)	99.3 (11519)	98.8 (13489)	99.1 (24924)	98.8 (12572)	100 (33423)	98.9 (20107)	99.2 (5490)
GSE77841	98.9 (3864)	98.9 (7985)	98 (4158)	97.7 (11030)	93.9 (7992)	97.7 (11884)	98.6 (8665)	97.5 (12025)	98.7 (9040)	97.6 (7678)	98.6 (11860)	100 (15351)	98.8 (12572)	98.5 (11275)	98.5 (5037)
GSE75440	98.9 (4253)	99.4 (9557)	99.2 (5564)	97.7 (15565)	96.8 (18310)	98.7 (24142)	99.2 (11212)	98.7 (22838)	99.4 (11288)	98.9 (15688)	100 (87571)	98.6 (11860)	99.1 (24924)	98.6 (22922)	99.1 (5527)
GSE73588	97.6 (3602)	98.5 (5764)	98.2 (4636)	96 (8525)	91.9 (13779)	96.5 (13563)	98.3 (8012)	96.9 (13995)	98.6 (7733)	100 (26273)	98.9 (15688)	97.6 (7678)	98.8 (13489)	98.4 (11714)	98.1 (4056)
GSE73317	99.1 (3591)	99.4 (8200)	99.1 (4313)	98 (8847)	94.7 (7779)	98.5 (11368)	99.2 (9011)	98.5 (11389)	100 (12065)	98.6 (7733)	99.4 (11288)	98.7 (9040)	99.3 (11519)	99.1 (10353)	99 (5025)
GSE71900	97.5 (4096)	98.6 (9927)	98 (5225)	95.5 (16079)	93.1 (15705)	96.9 (21512)	98.2 (11092)	100 (40944)	98.5 (11389)	96.9 (13995)	98.7 (22838)	97.5 (12025)	98.5 (22704)	98.4 (18508)	97.7 (5418)
GSE65030	98.8 (3450)	99.1 (7608)	98.9 (4278)	97.6 (8512)	94.6 (7883)	98.4 (10916)	100 (12266)	98.2 (11092)	99.2 (9011)	98.3 (8012)	99.2 (11212)	98.6 (8665)	99.3 (11364)	99.1 (9974)	98.9 (5107)
GSE52429	97.5 (4111)	98.5 (9838)	98 (5220)	95.5 (15938)	91.9 (14452)	100 (46738)	98.4 (10916)	96.9 (21512)	98.5 (11368)	96.5 (13563)	98.7 (24142)	97.7 (11884)	98.5 (21582)	98.4 (18458)	97.6 (5612)
GSE51254	93.2 (3578)	94.8 (5994)	94.2 (4470)	90 (9986)	100 (67446)	91.9 (14452)	94.6 (7883)	93.1 (15705)	94.7 (7779)	91.9 (13779)	96.8 (18310)	93.9 (7992)	95.7 (14829)	95.1 (13230)	93 (4158)
GSE47488	97.4 (4049)	97.8 (8477)	97.2 (3988)	100 (51201)	90 (9986)	95.5 (15938)	97.6 (8512)	95.5 (16079)	98 (8847)	96 (8525)	97.7 (15565)	97.7 (11030)	97.9 (15733)	97.4 (14101)	96.9 (4570)
GSE45332	98.9 (2423)	99.1 (3667)	100 (6155)	97.2 (3988)	94.2 (4470)	98 (5220)	98.9 (4278)	98 (5225)	99.1 (4313)	98.2 (4636)	99.2 (5564)	98 (4158)	99.1 (5332)	99.1 (5125)	98.7 (2745)
GSE30268	98.8 (3143)	100 (10528)	99.1 (3667)	97.8 (8477)	94.8 (5994)	98.5 (9838)	99.1 (7608)	98.6 (9927)	99.4 (8200)	98.5 (5764)	99.4 (9557)	98.9 (7985)	99.4 (10097)	99.1 (9029)	99 (4453)
GSE28254	99.9 (4971)	98.8 (3143)	98.9 (2423)	97.4 (4049)	93.2 (3578)	97.5 (4111)	98.8 (3450)	97.5 (4096)	99.1 (3591)	97.6 (3602)	98.9 (4253)	98.9 (3864)	98.9 (4103)	98.9 (3991)	98.6 (2484)

HeLa

	GSE42420	GSE45157	GSE45504	GSE48286	GSE51261	GSE53327	GSE56862	GSE58838	GSE63555	GSE63953	GSE64615	GSE65644	GSE68471	GSE75247	GSE75410	GSE77913	GSE79664	GSE81460
GSE81460	94.9 (40255)	91.7 (39182)	93.5 (38480)	90.3 (17011)	90.1 (35505)	92.1 (28001)	72 (25545)	92.9 (30697)	88.5 (20106)	92.1 (27251)	88.3 (18652)	89.1 (41315)	55.6 (5)	94.3 (11117)	91.6 (18209)	97.9 (22995)	94.1 (6793)	100 (51200)
GSE79664	90.6 (6757)	92.3 (7868)	94.5 (7441)	91.8 (7072)	91.7 (6960)	94.1 (7449)	74.9 (6645)	94.8 (8053)	87 (7757)	93.6 (7836)	87.7 (7682)	89.5 (7659)	62.5 (4)	97.4 (6879)	96.4 (7067)	94.3 (5891)	100 (10375)	94.1 (6793)
GSE77913	94.2 (23153)	90.7 (22312)	92.8 (21782)	89.5 (12807)	89.4 (21096)	91.4 (19654)	72.2 (15235)	92.3 (19509)	87.3 (15197)	91.4 (18045)	87.5 (14380)	88.3 (22189)		93.9 (8125)	92.7 (13321)	100 (25824)	94.3 (5891)	97.9 (22995)
GSE75410	89 (18898)	91.6 (22803)	92.6 (21058)	89.4 (15468)	90.7 (18666)	91.8 (18595)	66.2 (22635)	90.1 (23370)	88.6 (15407)	90.2 (20770)	89.8 (14580)	86 (25133)	57.1 (3)	93.8 (11886)	100 (66550)	92.7 (13321)	96.4 (7067)	91.6 (18209)
GSE75247	91.9 (10917)	95 (14445)	95.8 (12932)	93.2 (14325)	95 (11452)	95.2 (13421)	74.4 (19448)	95.3 (21545)	90.3 (13776)	94.6 (16741)	90.5 (13066)	91.8 (16541)	70 (6)	100 (35054)	93.8 (11886)	93.9 (8125)	97.4 (6879)	94.3 (11117)
GSE68471				71.4 (10)		52.9 (13)	63.6 (7)	72.2 (14)	58.3 (8)	75 (8)	61.5 (9)	60 (11)	95.8 (68)	70 (6)	57.1 (3)		62.5 (4)	55.6 (5)
GSE65644	85.9 (60848)	86.6 (73887)	88.9 (66301)	86.1 (34940)	85.4 (59005)	88.3 (46418)	70.9 (71349)	90.3 (64938)	81 (34381)	88.1 (58484)	80.9 (30259)	100 (425215)	60 (11)	91.8 (16541)	86 (25133)	88.3 (22189)	89.5 (7659)	89.1 (41315)
GSE64615	78.9 (26127)	78.7 (33232)	85.6 (24855)	80.5 (21108)	75.9 (28283)	85.2 (22472)	70.1 (19146)	88.8 (23113)	79.9 (57152)	84.8 (26065)	100 (163533)	80.9 (30259)	61.5 (9)	90.5 (13066)	89.8 (14580)	87.5 (14380)	87.7 (7682)	88.3 (18652)
GSE63953	86.6 (34616)	88.6 (40949)	91.3 (36617)	90.8 (52211)	89.2 (36239)	93.7 (39394)	73.5 (55781)	95.7 (58500)	84.4 (28876)	100 (207844)	84.8 (26065)	88.1 (58484)	75 (8)	94.6 (16741)	90.2 (20770)	91.4 (18045)	93.6 (7836)	92.1 (27251)
GSE63555	78.3 (28769)	78.5 (36639)	85.6 (27199)	80.2 (23129)	75.7 (31170)	84.9 (24649)	70 (22052)	88.7 (26127)	100 (190066)	84.4 (28876)	79.9 (57152)	81 (34381)	58.3 (8)	90.3 (13776)	88.6 (15407)	87.3 (15197)	87 (7757)	88.5 (20106)
GSE58838	89.3 (37550)	92.3 (43482)	93.7 (41411)	94.3 (46142)	92.2 (39818)	95.1 (45954)	74 (80435)	100 (178729)	88.7 (26127)	95.7 (58500)	88.8 (23113)	90.3 (64938)	72.2 (14)	95.3 (21545)	90.1 (23370)	92.3 (19509)	94.8 (8053)	92.9 (30697)
GSE56862	71.2 (30029)	74.2 (38051)	74.2 (37722)	71.1 (40327)	71.7 (30442)	72.9 (36440)	100 (310798)	74 (80435)	70 (22052)	73.5 (55781)	70.1 (19146)	70.9 (71349)	63.6 (7)	74.4 (19448)	66.2 (22635)	72.2 (15235)	74.9 (6645)	72 (25545)
GSE53327	88.6 (34072)	90.9 (38524)	92.8 (34718)	92 (29401)	91.3 (35888)	100 (91383)	72.9 (36440)	95.1 (45954)	84.9 (24649)	93.7 (39394)	85.2 (22472)	88.3 (46418)	52.9 (13)	95.2 (13421)	91.8 (18595)	91.4 (19654)	94.1 (7449)	92.1 (28001)
GSE51261	86.1 (47264)	86.7 (55797)	89.5 (47640)	86.8 (23182)	100 (133104)	91.3 (35888)	71.7 (30442)	92.2 (39818)	75.7 (31170)	89.2 (36239)	75.9 (28283)	85.4 (59005)		95 (11452)	90.7 (18666)	89.4 (21096)	91.7 (6960)	90.1 (35505)
GSE48286	84.5 (20601)	86.5 (26047)	89 (22216)	100 (157296)	86.8 (23182)	92 (29401)	71.1 (40327)	94.3 (46142)	80.2 (23129)	90.8 (52211)	80.5 (21108)	86.1 (34940)	71.4 (10)	93.2 (14325)	89.4 (15468)	89.5 (12807)	91.8 (7072)	90.3 (17011)
GSE45504	89.7 (46639)	91.7 (59426)	100 (107754)	89 (22216)	89.5 (47640)	92.8 (34718)	74.2 (37722)	93.7 (41411)	85.6 (27199)	91.3 (36617)	85.6 (24855)	88.9 (66301)		95.8 (12932)	92.6 (21058)	92.8 (21782)	94.5 (7441)	93.5 (38480)
GSE45157	86.7 (52523)	100 (162087)	91.7 (59426)	86.5 (26047)	86.7 (55797)	90.9 (38524)	74.2 (38051)	92.3 (43482)	78.5 (36639)	88.6 (40949)	78.7 (33232)	86.6 (73887)		95 (14445)	91.6 (22803)	90.7 (22312)	92.3 (7868)	91.7 (39182)
GSE42420	100 (140345)	86.7 (52523)	89.7 (46639)	84.5 (20601)	86.1 (47264)	88.6 (34072)	71.2 (30029)	89.3 (37550)	78.3 (28769)	86.6 (34616)	78.9 (26127)	85.9 (60848)		91.9 (10917)	89 (18898)	94.2 (23153)	90.6 (6757)	94.9 (40255)

K562

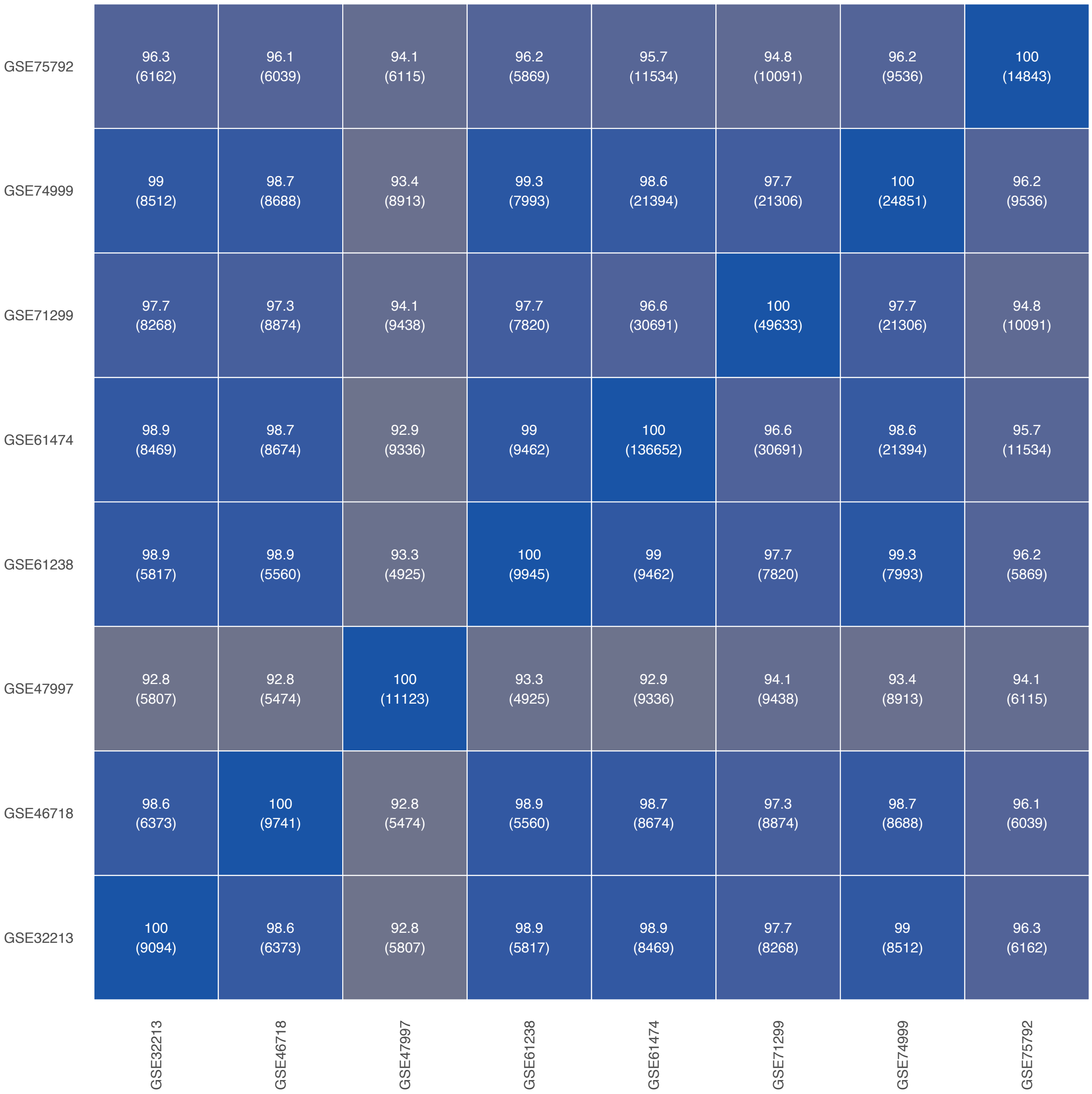
GSE75792
96.3 (6162)
96.1 (6039)
94.1 (6115)
96.2 (5869)
95.7 (11534)
94.8 (10091)
96.2 (9536)
100 (14843)
GSE74999
99 (8512)
98.7 (8688)
93.4 (8913)
99.3 (7993)
98.6 (21394)
97.7 (21306)
100 (24851)
96.2 (9536)
GSE71299
97.7 (8268)
97.3 (8874)
94.1 (9438)
97.7 (7820)
96.6 (30691)
100 (49633)
97.7 (21306)
94.8 (10091)
GSE61474
98.9 (8469)
98.7 (8674)
92.9 (9336)
99 (9462)
100 (136652)
96.6 (30691)
98.6 (21394)
95.7 (11534)
GSE61238
98.9 (5817)
98.9 (5560)
93.3 (4925)
100 (9945)
99 (9462)
97.7 (7820)
99.3 (7993)
96.2 (5869)
GSE47997
92.8 (5807)
92.8 (5474)
100 (11123)
93.3 (4925)
92.9 (9336)
94.1 (9438)
93.4 (8913)
94.1 (6115)
GSE46718
98.6 (6373)
100 (9741)
92.8 (5474)
98.9 (5560)
98.7 (8674)
97.3 (8874)
98.7 (8688)
96.1 (6039)
GSE32213
100 (9094)
98.6 (6373)
92.8 (5807)
98.9 (5817)
98.9 (8469)
97.7 (8268)
99 (8512)
96.3 (6162)
GSE32213
GSE46718
GSE47997
GSE61238
GSE61474
GSE71299
GSE74999
GSE75792

MCF7

	GSE52385	GSE54693	GSE59251	GSE59536	GSE63189	GSE68228	GSE69604	GSE71862	GSE74232	GSE76608	GSE77033	GSE81469
GSE81469	67.3 (6413)	68.5 (567)	68 (1973)	68.3 (1017)	67.1 (7435)	67.9 (9489)	68.3 (11453)	67.2 (3662)	67.6 (4045)	69 (8609)	66.7 (23)	100 (22771)
GSE77033	70.4 (23)			62.5 (12)	66.7 (5)	61.5 (22)	64.7 (30)	57.1 (3)	81.8 (18)	89.7 (25)	93.5 (42)	66.7 (23)
GSE76608	95.4 (10642)	97 (1054)	97.9 (3267)	91 (1688)	97.2 (12833)	95 (16399)	96 (21447)	98 (6725)	97.9 (6787)	100 (24331)	89.7 (25)	69 (8609)
GSE74232	95.3 (5799)	96.8 (783)	97.2 (2683)	91.2 (1496)	97.1 (5806)	95.1 (6842)	96 (7374)	97.8 (3987)	100 (8296)	97.9 (6787)	81.8 (18)	67.6 (4045)
GSE71862	95.2 (5465)	97.1 (902)	96.9 (2552)	89.2 (1145)	97.5 (5775)	95.1 (6346)	96.9 (6956)	100 (7955)	97.8 (3987)	98 (6725)	57.1 (3)	67.2 (3662)
GSE69604	95.7 (12775)	97.9 (1054)	96.6 (3340)	90.9 (1826)	96.1 (17131)	95.5 (24972)	100 (91174)	96.9 (6956)	96 (7374)	96 (21447)	64.7 (30)	68.3 (11453)
GSE68228	97.1 (11547)	96.2 (989)	94.9 (3226)	91.9 (1694)	94.4 (14052)	100 (28355)	95.5 (24972)	95.1 (6346)	95.1 (6842)	95 (16399)	61.5 (22)	67.9 (9489)
GSE63189	95 (10125)	96.1 (969)	97.3 (3244)	90.8 (1305)	100 (18908)	94.4 (14052)	96.1 (17131)	97.5 (5775)	97.1 (5806)	97.2 (12833)	66.7 (5)	67.1 (7435)
GSE59536	91.4 (1561)	90.5 (376)	89.4 (845)	99.9 (2053)	90.8 (1305)	91.9 (1694)	90.9 (1826)	89.2 (1145)	91.2 (1496)	91 (1688)	62.5 (12)	68.3 (1017)
GSE59251	94.5 (3098)	95.4 (719)	99.9 (3521)	89.4 (845)	97.3 (3244)	94.9 (3226)	96.6 (3340)	96.9 (2552)	97.2 (2683)	97.9 (3267)		68 (1973)
GSE54693	95.2 (940)	99.7 (1131)	95.4 (719)	90.5 (376)	96.1 (969)	96.2 (989)	97.9 (1054)	97.1 (902)	96.8 (783)	97 (1054)		68.5 (567)
GSE52385	100 (13478)	95.2 (940)	94.5 (3098)	91.4 (1561)	95 (10125)	97.1 (11547)	95.7 (12775)	95.2 (5465)	95.3 (5799)	95.4 (10642)	70.4 (23)	67.3 (6413)

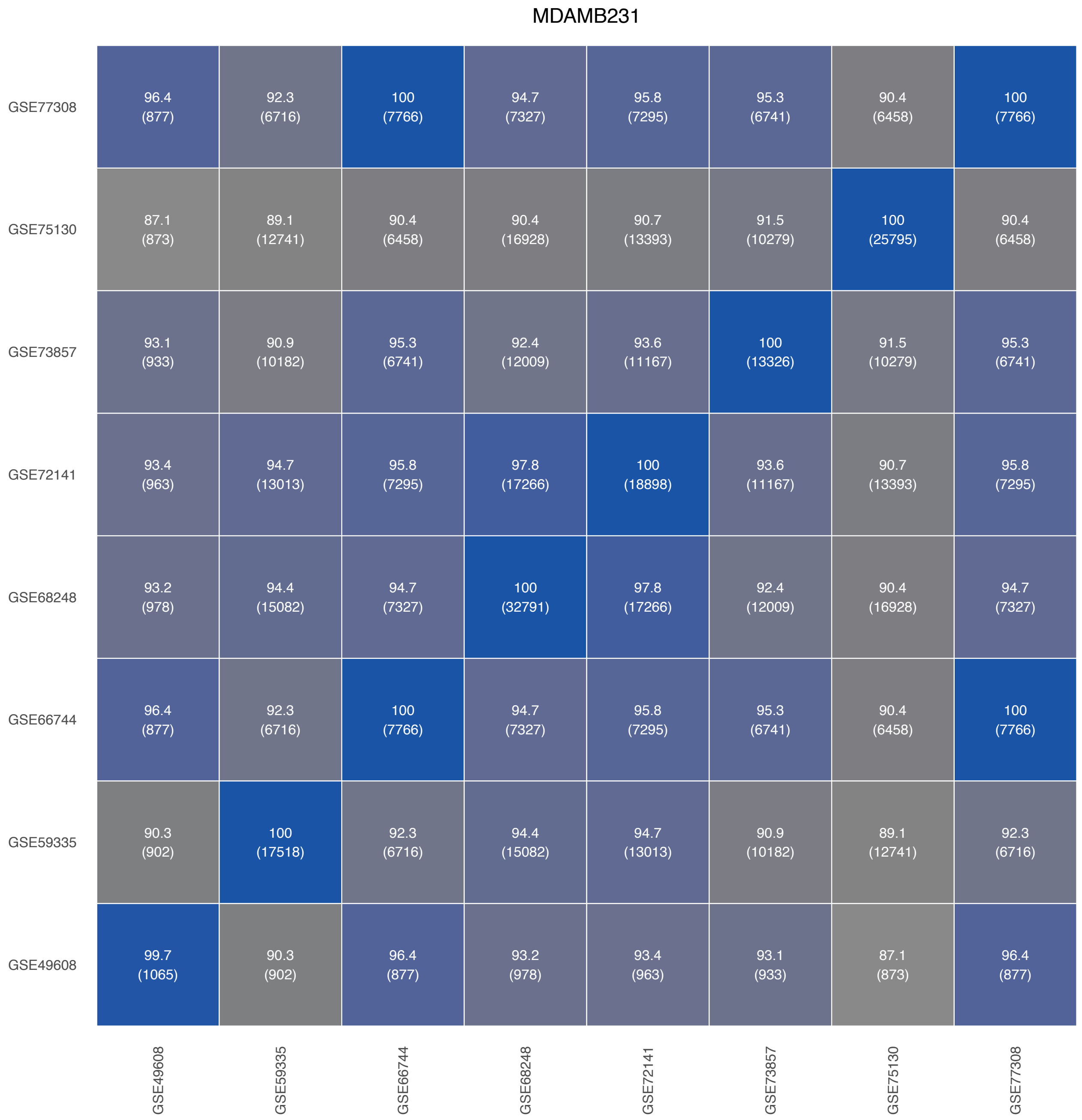
MDAMB231
GSE77308
96.4 (877)
92.3 (6716)
100 (7766)
94.7 (7327)
95.8 (7295)
95.3 (6741)
90.4 (6458)
100 (7766)
GSE75130
87.1 (873)
89.1 (12741)
90.4 (6458)
90.4 (16928)
90.7 (13393)
91.5 (10279)
100 (25795)
90.4 (6458)
GSE73857
93.1 (933)
90.9 (10182)
95.3 (6741)
92.4 (12009)
93.6 (11167)
100 (13326)
91.5 (10279)
95.3 (6741)
GSE72141
93.4 (963)
94.7 (13013)
95.8 (7295)
97.8 (17266)
100 (18898)
93.6 (11167)
90.7 (13393)
95.8 (7295)
GSE68248
93.2 (978)
94.4 (15082)
94.7 (7327)
100 (32791)
97.8 (17266)
92.4 (12009)
90.4 (16928)
94.7 (7327)
GSE66744
96.4 (877)
92.3 (6716)
100 (7766)
94.7 (7327)
95.8 (7295)
95.3 (6741)
90.4 (6458)
100 (7766)
GSE59335
90.3 (902)
100 (17518)
92.3 (6716)
94.4 (15082)
94.7 (13013)
90.9 (10182)
89.1 (12741)
92.3 (6716)
GSE49608
99.7 (1065)
90.3 (902)
96.4 (877)
93.2 (978)
93.4 (963)
93.1 (933)
87.1 (873)
96.4 (877)
GSE49608
GSE59335
GSE66744
GSE68248
GSE72141
GSE73857
GSE75130
GSE77308

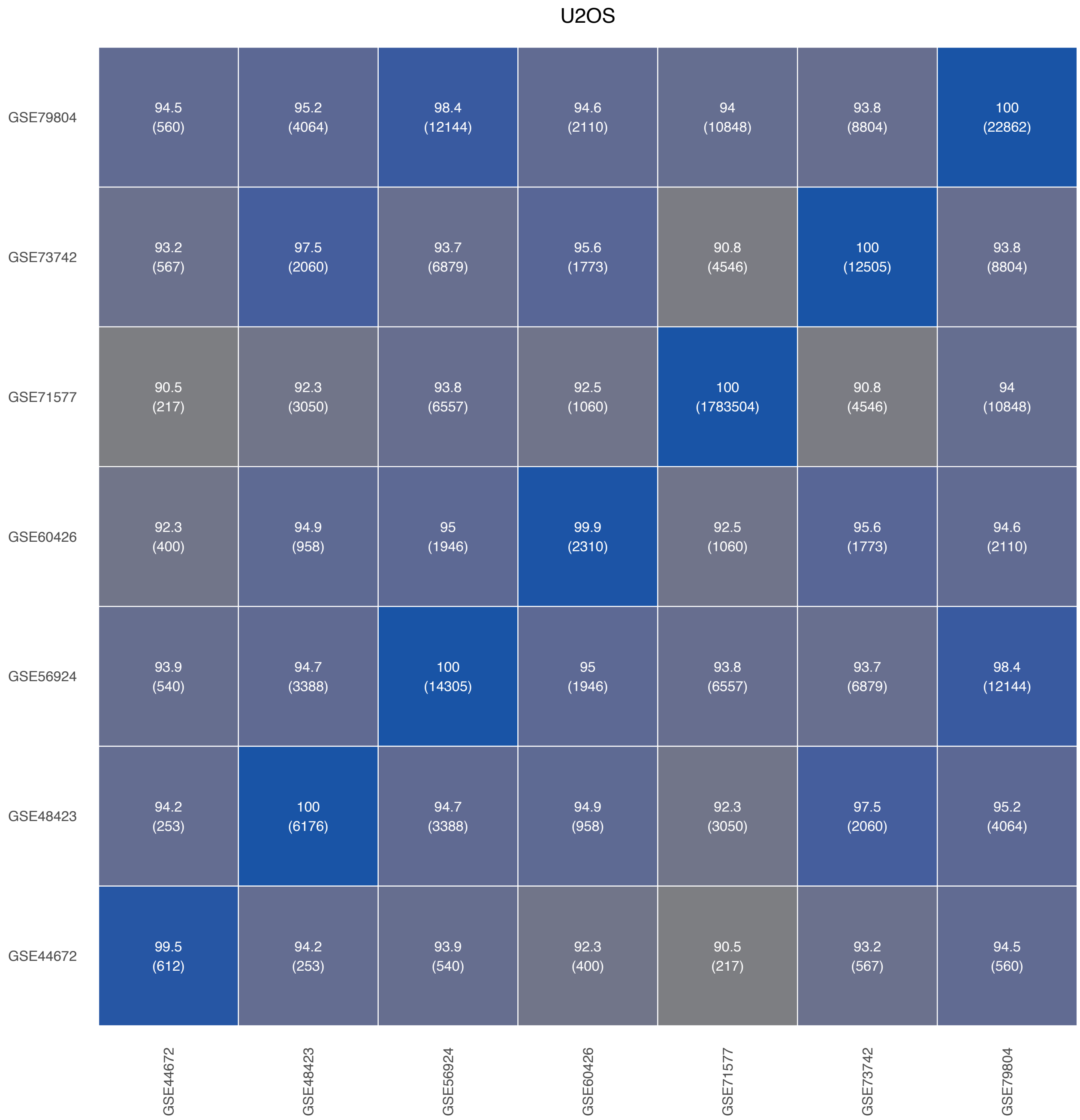
U2OS
GSE79804
94.5 (560)
95.2 (4064)
98.4 (12144)
94.6 (2110)
94 (10848)
93.8 (8804)
100 (22862)
GSE73742
93.2 (567)
97.5 (2060)
93.7 (6879)
95.6 (1773)
90.8 (4546)
100 (12505)
93.8 (8804)
GSE71577
90.5 (217)
92.3 (3050)
93.8 (6557)
92.5 (1060)
100 (1783504)
90.8 (4546)
94 (10848)
GSE60426
92.3 (400)
94.9 (958)
95 (1946)
99.9 (2310)
92.5 (1060)
95.6 (1773)
94.6 (2110)
GSE56924
93.9 (540)
94.7 (3388)
100 (14305)
95 (1946)
93.8 (6557)
93.7 (6879)
98.4 (12144)
GSE48423
94.2 (253)
100 (6176)
94.7 (3388)
94.9 (958)
92.3 (3050)
97.5 (2060)
95.2 (4064)
GSE44672
99.5 (612)
94.2 (253)
93.9 (540)
92.3 (400)
90.5 (217)
93.2 (567)
94.5 (560)
GSE44672
GSE48423
GSE56924
GSE60426
GSE71577
GSE73742
GSE79804

Supplementary figure 13

Visualisation of KEGGM functional unit enrichments across similarity score distributions.

```
# Cells with significant correlations
cells.sig <- c("MDAMB231", "MCF7", "HCT116", "H9")
data.degs <- data.degs[data.degs$cell %in% cells.sig, ]
data.degs$cell <- factor(data.degs$cell, levels = cells.sig)

# MDAMB231 bins
mdamb231 <- data.degs[data.degs$cell == "MDAMB231", ]
range <- (max(mdamb231$score) - min(mdamb231$score)) / 3
mdamb231_bins <- c(min(mdamb231$score) + range * 2,
                   min(mdamb231$score) + range,
                   min(mdamb231$score))

# Bins for similarity score
bins <- list(list(97.5, 97.49, 0),
             list(97.5, 90, 0),
             list(96.0, 90, 0),
             mdamb231_bins)

# Add bins to data
for (n in seq(1, 4)) {

    # Get bins for each cell
    current_list <- bins[[n]]
    cell <- rev(cells.sig)[n]

    # Add bins
    data.degs[data.degs$cell == cell &
              data.degs$score >= current_list[[3]] &
              data.degs$score < current_list[[2]], "bin"] <- "Low"
    data.degs[data.degs$cell == cell &
              data.degs$score >= current_list[[2]] &
              data.degs$score < current_list[[1]], "bin"] <- "Medium"
    data.degs[data.degs$cell == cell &
              data.degs$score >= current_list[[1]], "bin"] <- "High"
}

# Factorise bins
data.degs$bin <- factor(data.degs$bin,
                        levels = c("High", "Medium", "Low"))

# Convert to long format and get enriched KEGG modules
long <- melt(data.degs[c("cell", "enriched.1", "enriched.2", "enriched.3",
                         "enriched.4", "enriched.5", "enriched.6",
                         "enriched.7", "enriched.8", "enriched.9",
                         "enriched.10", "bin")],
             id.vars = c("cell", "bin"),
             value.name = "category")

# Remove empty enrichments
long <- long[!is.na(long$category), ]
```

```r
long$variable <- NULL

# Trim long KEGGM category names
long$category <- gsub(",.*", "", long$category)
long$category <- gsub("\\ \\(.*", "", long$category)

# Count frequency of categories
long <- long %>%
    group_by(cell, bin, category) %>%
    summarise(count = n())

# Reorder categories
long$category <- factor(long$category,
                        levels = rev(c("Ribosome",
                                       "NADH dehydrogenase",
                                       "Spliceosome",
                                       "Wnt signaling",
                                       "Cholesterol biosynthesis",
                                       "Hedgehog signaling",
                                       "Cytochrome bc1 complex",
                                       "F-type ATPase")))

# Colour palette
palette <- rev(c("#0d2d59", "#1954a6", "#4e8ce4", "#a6c6f2",
                 "#333333", "#666666", "#999999", "#cccccc"))
```

```r
# Add missing info for consistent facets
long[nrow(long) + 1, "cell"] <- "HCT116"
long[nrow(long), "bin"]      <- "Low"
long[nrow(long), "category"] <- ""
long[nrow(long) + 1, "cell"] <- "MCF7"
long[nrow(long), "bin"]      <- "Medium"
long[nrow(long), "category"] <- ""
long[nrow(long) + 1, "cell"] <- "MCF7"
long[nrow(long), "bin"]      <- "High"
long[nrow(long), "category"] <- ""

# Plot
sfig_13 <- ggplot(long, aes(x = bin, y = count, fill = category)) +
    geom_bar(stat = "identity", colour = "white") +
    theme_bw() +
    theme(panel.grid.major.x = element_blank(),
          panel.grid.minor   = element_blank(),
          strip.background   = element_blank(),
          plot.title         = element_text(hjust = 0.5)) +
          #plot.margin        = unit(c(40, 5, 5, 5), "pt")) +
    labs(x = "Relative similarity",
         y = "Frequency",
         fill = "KEGGM function") +
    scale_fill_manual(values = palette) +
    scale_y_continuous(limits = c(0, 8), breaks = seq(0, 8, by = 2)) +
    facet_grid(~ cell, scales = "free_x")

ggsave("../../images/sfigure_13.png", sfig_13, width = 15, height = 5)
```

```
sfig_13
```

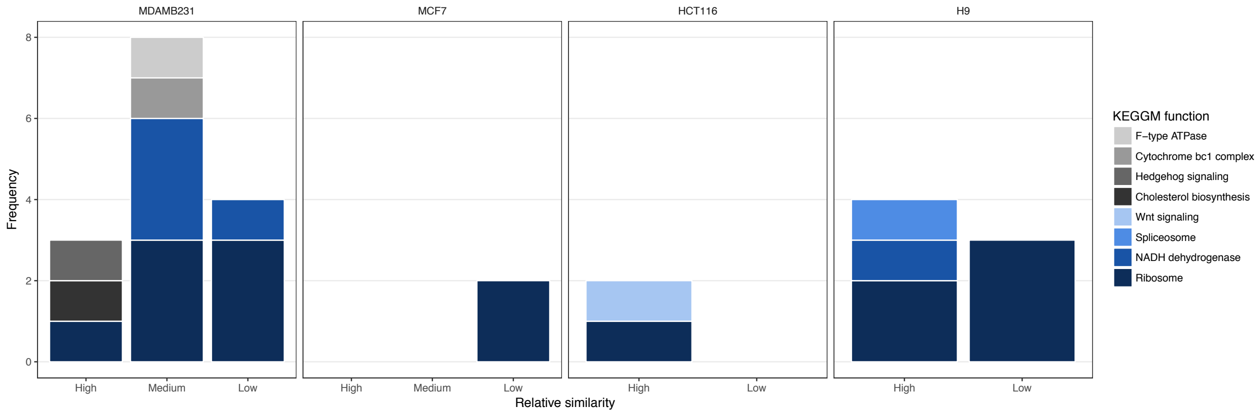

MDAMB231
MCF7
HCT116
H9
Frequency
0
2
4
6
8
High
Medium
Low
Relative similarity
KEGGM function
F−type ATPase
Cytochrome bc1 complex
Hedgehog signaling
Cholesterol biosynthesis
Wnt signaling
Spliceosome
NADH dehydrogenase
Ribosome

Supplementary figure 14

Distributions of similarity scores for each cell line with significant correlations with either number of DEGs or fold change of DEGs, used to pick similarity groups for *Sfigure 13*. For calculations, please see the section of *Figure 4* (above).

```
plot_list <- list()
for (cell in unique(data.degs$cell)) {
    current <- data.degs[data.degs$cell == cell, ]
    gg <- ggplot(current, aes(x = score, fill = bin)) +
        geom_histogram(binwidth = 1) +
        theme_bw() +
        theme(legend.position  = "none",
              plot.title       = element_text(hjust = 0.5)) +
        ylim(c(0, 8)) +
        labs(title = cell,
             x     = "Similarity score",
             y     = "Count (comparisons)") +
        scale_fill_manual(values = c("#0d2d59", "#1954a6", "#4e8ce4")) +
        scale_y_continuous(breaks = seq(0, 10, 2))
    plot_list[[length(plot_list) + 1]] <- gg
}
```

```
## Scale for 'y' is already present. Adding another scale for 'y', which
## will replace the existing scale.
## Scale for 'y' is already present. Adding another scale for 'y', which
## will replace the existing scale.
## Scale for 'y' is already present. Adding another scale for 'y', which
## will replace the existing scale.
## Scale for 'y' is already present. Adding another scale for 'y', which
## will replace the existing scale.
```

```
sfig_14 <- cowplot::plot_grid(plotlist = plot_list, ncol = 2)
```

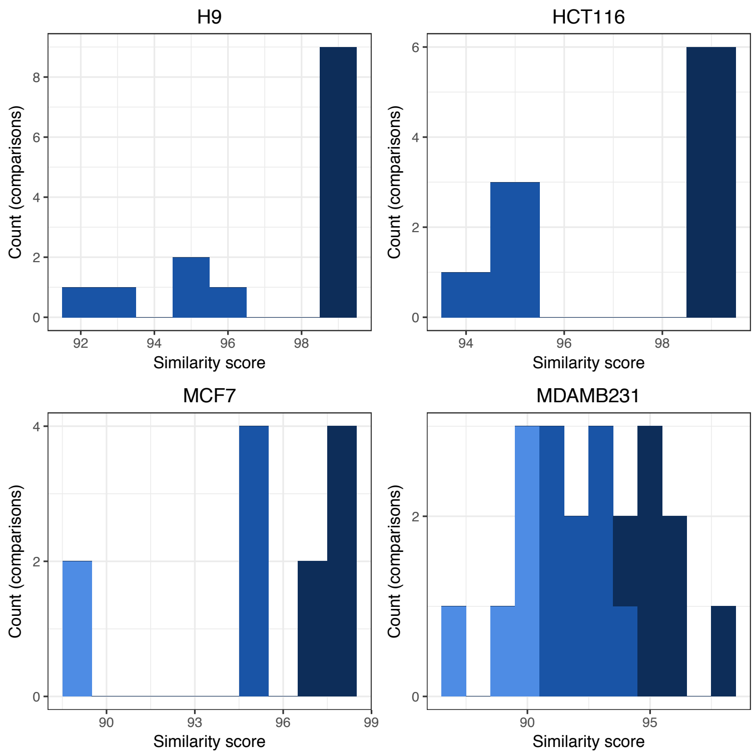
H9
HCT116
MCF7
MDAMB231
Count (comparisons)
Similarity score
0
2
4
6
8
92
94
96
98
90
93
99
95

Supplementary figure 15

Correlations between the similarity score and Pearson correlations of all expressed genes (measures in TPM). All correlations were performed with log-normalised (TPM + 1) measures.

```
# Set current data to expression correlation data
data <- data.expr

# Initialise correlations list
corrs <- data.frame(cell             = character(),
                    corr             = numeric(),
                    pval             = numeric(),
                    stringsAsFactors = FALSE)

# Correlation type
type <- "pearson"

# Correlate per cell
for (cell in unique(data$cell)) {

    # Subset for current cell line
    current <- data[data$cell == cell, ]

    # Correlations
    corr <- cor.test(x      = current[[paste0(type, "_r2")]],
                     y      = current$score,
                     method = type,
                     use    = "complete.obs")

    # Add siginificant correlations
    corrs <- rbind(corrs, data.frame(cell = cell,
                                     corr = corr$estimate,
                                     pval = corr$p.value))
}

# Merge correlations with data
data <- merge(data, corrs, by = "cell")

# Remove non-significant correlations
data <- data[abs(data$corr) >= 0.25 & data$pval <= 0.01, ]

# Labels
data$label <- paste0(data$cell, "\n(Correlation coefficient = ",
                     format(data$corr, digits = 2),
                     ", p-value = ", format(data$pval, digits = 2), ")")

# Plot
plot_list <- list()
for (label in unique(data$label)) {
    current <- data[data$label == label, ]
    gg <- ggplot(current, aes(x = pearson_r2, y = score)) +
        geom_point(colour = "#1954a6",
                   alpha = 0.5,
                   size = 1,
```

```r
                   aes(shape = "a")) +
        geom_smooth(method = "lm", se = FALSE, colour = "#0d2d59") +
        theme_bw() +
        labs(title = label,
             x     = expression(paste("Expression correlation (", R ^ 2, ")")),
             y     = "Similarity score") +
        theme(legend.position = "none",
              plot.title      = element_text(hjust = 0.5, size = 8))
    plot_list[[length(plot_list) + 1]] <- gg
}

# Save plot
sfig_15 <- cowplot::plot_grid(plotlist = plot_list, ncol = 2)
ggsave("../../images/sfigure_15.png", sfig_15, dpi = 300, width = 7,
       height = 10.5)
sfig_15
```

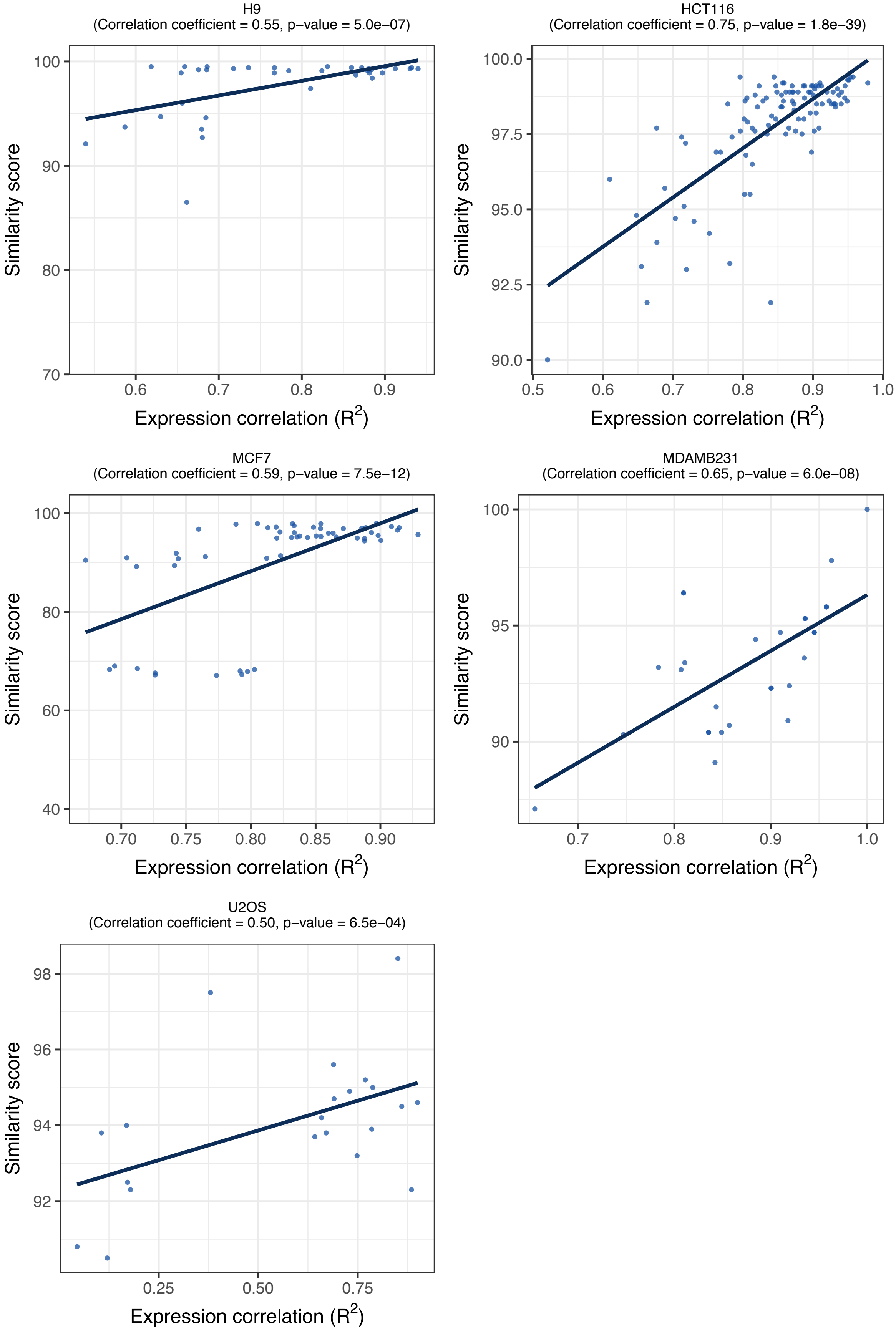
H9
(Correlation coefficient = 0.55, p−value = 5.0e−07)
Similarity score
Expression correlation (R2)
HCT116
(Correlation coefficient = 0.75, p−value = 1.8e−39)
Similarity score
Expression correlation (R2)
MCF7
(Correlation coefficient = 0.59, p−value = 7.5e−12)
Similarity score
Expression correlation (R2)
MDAMB231
(Correlation coefficient = 0.65, p−value = 6.0e−08)
Similarity score
Expression correlation (R2)
U2OS
(Correlation coefficient = 0.50, p−value = 6.5e−04)
Similarity score
Expression correlation (R2)

Supplementary figure 16

Correlations between the similarity score and Pearson correlations of prognostic genes (expression measures in TPM). All correlations were performed with log-normalised (TPM + 1) measures.

```r
# Set current data to expression correlation data
data <- data.expr.prog

# Initialise correlations list
corrs <- data.frame(cell             = character(),
                    corr             = numeric(),
                    pval             = numeric(),
                    stringsAsFactors = FALSE)

# Correlate per cell
for (cell in unique(data$cell)) {

    # Skip cell lines without corresponding HPA tissue
    if (cell %in% c("H9", "K562", "U2OS")) {
        next
    }

    # Subset for current cell line
    current <- data[data$cell == cell, ]

    # Correlations
    corr <- cor.test(x      = current$pearson_r2,
                     y      = current$score,
                     method = "pearson",
                     use    = "complete.obs")

    # Add siginificant correlations
    corrs <- rbind(corrs, data.frame(cell = cell,
                                     corr = corr$estimate,
                                     pval = corr$p.value))
}

# Merge correlations with data
data <- merge(data, corrs, by = "cell")

# Remove non-significant correlations
data <- data[abs(data$corr) >= 0.25 & data$pval <= 0.01, ]

# Labels
data$label <- paste0(data$cell, "\n(Correlation coefficient = ",
                     format(data$corr, digits = 2),
                     ", p-value = ", format(data$pval, digits = 2), ")")

# Plot
plot_list <- list()
for (label in unique(data$label)) {
    current <- data[data$label == label, ]
    gg <- ggplot(current, aes(x = pearson_r2, y = score)) +
        geom_point(colour = "#1954a6",
```

```r
                    alpha = 0.5,
                    size = 1,
                    aes(shape = "a")) +
        geom_smooth(method = "lm", se = FALSE, colour = "#0d2d59") +
        theme_bw() +
        labs(title = label,
             x     = expression(paste("Expression correlation (", R ^ 2, ")")),
             y     = "Similarity score") +
        theme(legend.position = "none",
              plot.title      = element_text(hjust = 0.5, size = 8))
    plot_list[[length(plot_list) + 1]] <- gg
}

# Save plot
sfig_16 <- cowplot::plot_grid(plotlist = plot_list, ncol = 2)
ggsave("../../images/sfigure_16.png", sfig_16, dpi = 300, width = 7,
       height = 7)
sfig_16
```

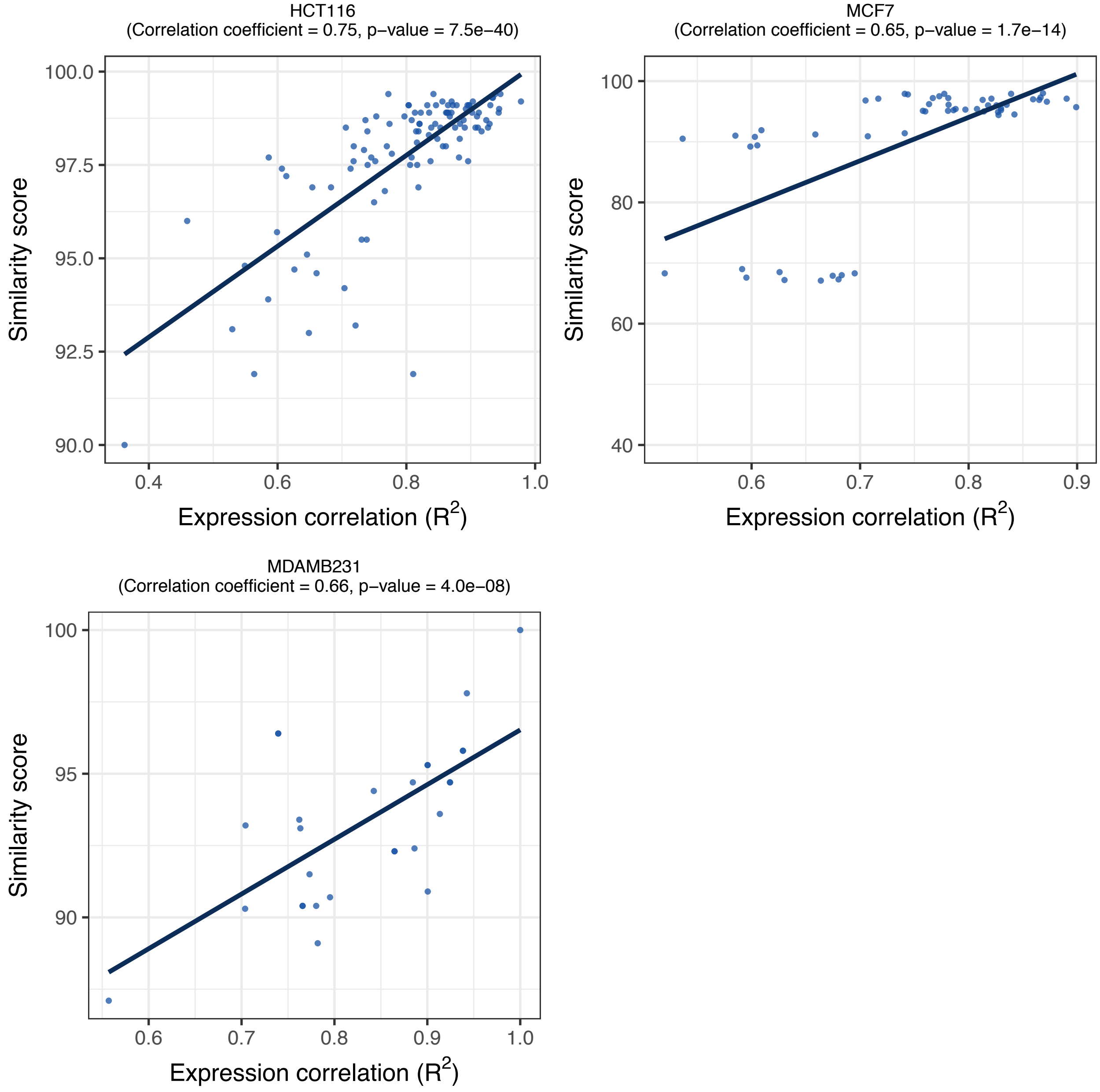
HCT116
(Correlation coefficient = 0.75, p−value = 7.5e−40)
Similarity score
100.0
97.5
95.0
92.5
90.0
0.4
0.6
0.8
1.0
Expression correlation (R^2)
MCF7
(Correlation coefficient = 0.65, p−value = 1.7e−14)
Similarity score
100
80
60
40
0.6
0.7
0.8
0.9
Expression correlation (R^2)
MDAMB231
(Correlation coefficient = 0.66, p−value = 4.0e−08)
Similarity score
100
95
90
0.6
0.7
0.8
0.9
1.0
Expression correlation (R^2)

Supplementary figure 17

Individual transriptome-wide impact distributions for each studied cell line.

```
# Get transcriptome SNV data
data <- data.transc.snvs

# Merge with cell line metadata
data <- merge(data, metadata, by.x = "sample_1", by.y = "GSE")
data <- merge(data, metadata, by.x = "sample_2", by.y = "GSE")

# Keep only comparisons within same cell lines
data <- data[data$cell.line.x == data$cell.line.y, ]

# Summary statistics
stats <- data %>%
    group_by(cell.line.x, match, impact) %>%
    summarise(count = n()) %>%
    mutate(prop = round(count / sum(count) * 100, 1))

stats$impact <- factor(stats$impact,
                       levels = c("HIGH", "MODERATE", "LOW", "MODIFIER"))
stats$match <- factor(stats$match, levels = c("yes", "no"))

# Plot impact for each cell line
plot_list <- list()
labels <- c("A", "B", "C", "D", "E", "F", "G", "H")
n <- 1
for (cell in unique(stats$cell.line.x)) {

    # Current cell line
    impact <- stats[stats$cell.line.x == cell, ]

    # Label
    label <- labels[n]

    gg <- ggplot(impact, aes(x = impact, y = prop, fill = match)) +
        geom_bar(stat = "identity", position = "dodge", color = "black", size = 0.3) +
        theme_bw() +
        labs(x = "\nImpact category", y = "Proportion of transcriptome-wide SNVs in category (%)") +
        geom_text(aes(label = count), position = position_dodge(width = 0.9), vjust = -0.5,
                size = 2.5) +
        geom_text(data = impact[impact$prop >= 2.5, ], aes(label = paste0(prop, " %")),
                position = position_dodge(width = 0.9), vjust = 1.6, size = 3.5,
                colour = "white") +
        geom_text(data = impact[impact$prop < 2.5, ], aes(y = -0.1, label = paste0(prop, " %")),
                position = position_dodge(width = 0.9), vjust = 1.6, size = 3.5,
                colour = "#4d4d4d") +
        scale_fill_manual(values = c("#0d2d59", "#1954a6")) +
        ylim(0, 85) +
        theme(legend.position = "none", axis.text = element_text(size = 14),
            panel.grid.major.x = element_blank(), axis.title = element_text(size = 15),
            plot.margin = unit(c(40, 5, 5, 5), "pt")) +
        annotation_custom(grob = textGrob(label = label, hjust = 0, vjust = -0.625,
```

```r
                                            gp = gpar(cex = 2.5)),
                             ymin = Inf, ymax = Inf, xmin = -Inf, xmax = -Inf)

    gg <- ggplot_gtable(ggplot_build(gg))
    gg$layout$clip[gg$layout$name == "panel"] <- "off"

    # Add to plot list
    plot_list[[n]] <- gg
    n <- n + 1
}

sfig_17 <- grid.arrange(plot_list[[1]],
                        plot_list[[2]],
                        plot_list[[3]],
                        plot_list[[4]],
                        plot_list[[5]],
                        plot_list[[6]],
                        plot_list[[7]],
                        plot_list[[8]], ncol = 3)
```

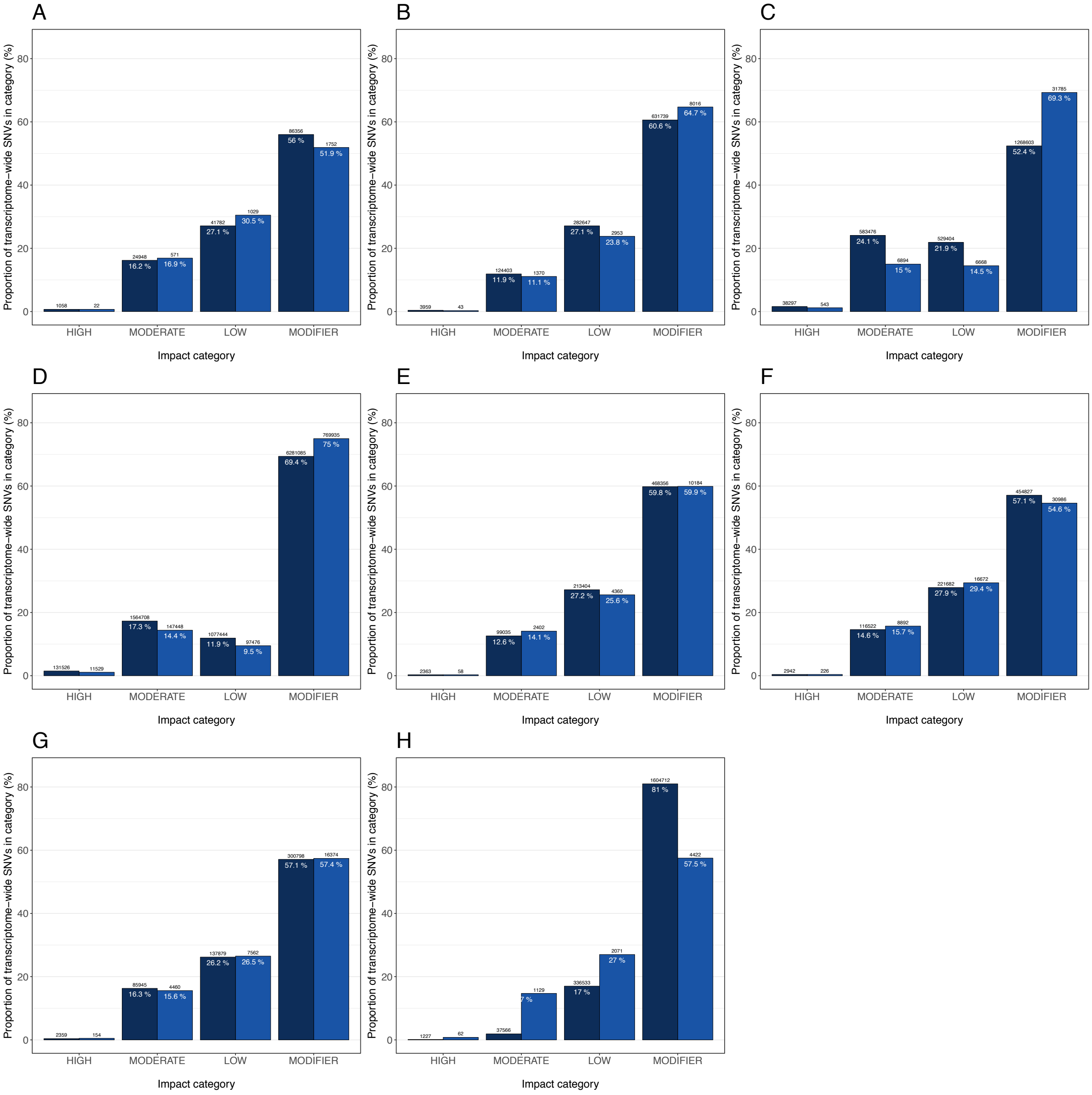
A
B
C
D
E
F
G
H
Proportion of transcriptome−wide SNVs in category (%)
Impact category
HIGH
MODERATE
LOW
MODIFIER
0
20
40
60
80
16.2 %
16.9 %
27.1 %
30.5 %
56 %
51.9 %
11.9 %
11.1 %
27.1 %
23.8 %
60.6 %
64.7 %
24.1 %
15 %
21.9 %
14.5 %
52.4 %
69.3 %
17.3 %
14.4 %
11.9 %
9.5 %
69.4 %
75 %
12.6 %
14.1 %
27.2 %
25.6 %
59.8 %
59.9 %
14.6 %
15.7 %
27.9 %
29.4 %
57.1 %
54.6 %
16.3 %
15.6 %
26.2 %
26.5 %
57.1 %
57.4 %
17 %
27 %
81 %
57.5 %

Supplementary figure 18

Confidence intervals for the ANOVA test in *Figure 4E*. For calculations, please see the section on *Figure 4* (above).

```
# Confidence interval plot
limit <- max(abs(tukey$lwr), tukey$upr) * 1.05
sfig_18 <- ggplot(tukey, aes(x = treatment, y = diff, ymin = lwr, ymax = upr,
                              colour = colour.groups)) +
    coord_flip(ylim = c(-limit, limit)) +
    geom_pointrange(shape = 20, size = 0.3) +
    geom_errorbar(width = 0.3) +
    theme_bw() +
    geom_hline(yintercept = 0, colour = "#4d4d4d", linetype = 2) +
    theme(panel.grid.major.x = element_blank(),
          panel.grid.minor.x = element_blank(),
          axis.text = element_text(size = 7),
          plot.title = element_text(hjust = 0.5),
          legend.position = "none") +
    labs(y = "Confidence interval" , x = NULL) +
    scale_colour_manual(values = c("#1954a6", "#a6a6a6"))
```

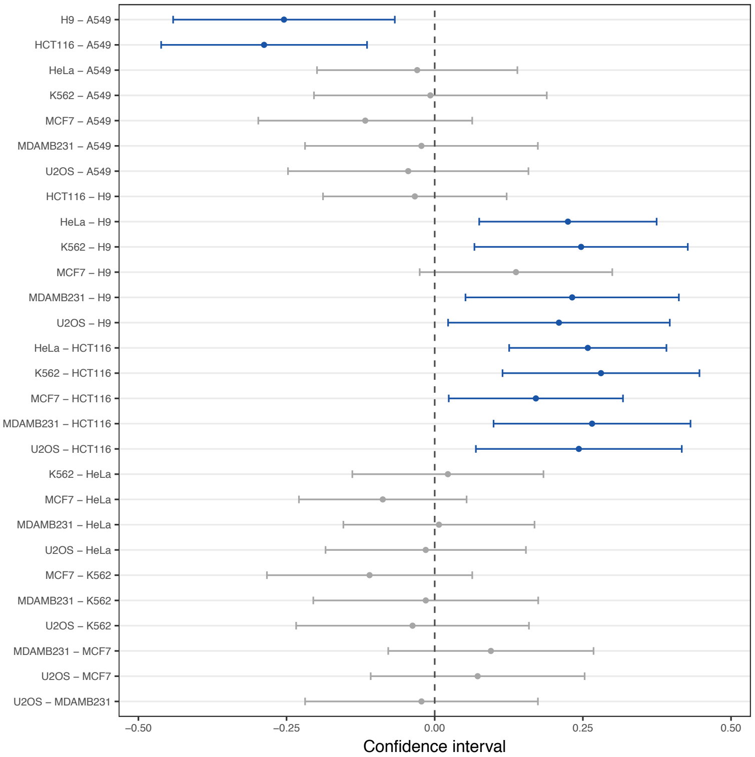

Supplementary figure 19

Characterisations of COSMIC variants for each cell line. The input file is not available in the supplementary data files, as COSMIC does not allow sharing of their data (see the COSMIC website to download the data yourself).

```
# Read COSMIC data
cosmic <- read.table("CosmicCLP_MutantExport.GRCh38.txt", header = TRUE,
                     sep = "\t", fill = TRUE, quote = "",
                     stringsAsFactors = FALSE)
```

```
# Fix column and cell line names
names(cosmic) <- tolower(gsub("\\.", "_", names(cosmic)))
cosmic$sample_name <- toupper(gsub("[-. ]", "", cosmic$sample_name))
cells <- c("A549", "H9", "HCT116", "HELA", "K562", "MCF7", "MDAMB231", "U2OS")
cosmic <- cosmic[cosmic$sample_name %in% cells, ]

# Keep unique SNVs
cosmic <- cosmic[grep("Substitution", cosmic$mutation_description), ]
cosmic <- cosmic[cosmic$mutation_genome_position != "", ]
cosmic$gene_name <- gsub("_ENST\\d+", "", cosmic$gene_name)
cosmic <- cosmic[!duplicated(cosmic[c("gene_name",
                                      "mutation_genome_position")]), ]

# Group and plot COSMIC verification status
verified <- cosmic %>%
    group_by(sample_name, mutation_verification_status) %>%
    summarise(count = n()) %>%
    mutate(prop = round(count / sum(count) * 100, 1))

gg.verified <- ggplot(verified, aes(x = sample_name, y = prop,
                                    fill = mutation_verification_status)) +
    geom_bar(stat = "identity", colour = "white") +
    theme_bw() +
    labs(x = NULL, y = "Proportion in category (%)",
         fill = "Verification status") +
    scale_fill_manual(values = c("#1954a6", "#0d2d59")) +
    theme(panel.grid.major = element_blank(),
          panel.grid.minor = element_blank(),
          legend.position = "bottom", legend.direction = "vertical")

# Group and plot COSMIC pathogenenicity
cosmic[cosmic$fathmm_score == "", "fathmm_score"] <- "[not listed]"
pathogenicity <- cosmic %>%
    group_by(sample_name, fathmm_score) %>%
    summarise(count = n()) %>%
    mutate(prop = round(count / sum(count) * 100, 1))

gg.patho <- ggplot(pathogenicity, aes(x = sample_name, y = prop,
                                      fill = fathmm_score)) +
    geom_bar(stat = "identity", colour = "white") +
    theme_bw() +
    labs(x = NULL, y = NULL, fill = "Pathogenicity status") +
    scale_fill_manual(values = c("#4e8ce4","#1954a6", "#0d2d59")) +
    theme(panel.grid.major = element_blank(),
```

```
          panel.grid.minor = element_blank(),
          legend.position = "bottom", legend.direction = "vertical")

    # Group and plot COSMIC variant types
mutation <- cosmic %>%
    group_by(sample_name, mutation_description) %>%
    summarise(count = n()) %>%
    mutate(prop = round(count / sum(count) * 100, 1))

gg.mutation <- ggplot(mutation, aes(x = sample_name, y = prop,
                                    fill = mutation_description)) +
    geom_bar(stat = "identity", colour = "white") +
    theme_bw() +
    labs(x = NULL, y = NULL, fill = "Mutation type") +
    scale_fill_manual(values = c("#4e8ce4","#1954a6", "#0d2d59")) +
    theme(panel.grid.major = element_blank(),
          panel.grid.minor = element_blank(),
          legend.position = "bottom", legend.direction = "vertical")

# Plot in grid
sfig_19 <- cowplot::plot_grid(gg.verified, gg.patho, gg.mutation, nrow = 1,
                              labels = c("A", "B", "C"), label_size = 14,
                              vjust = 1.05)
```

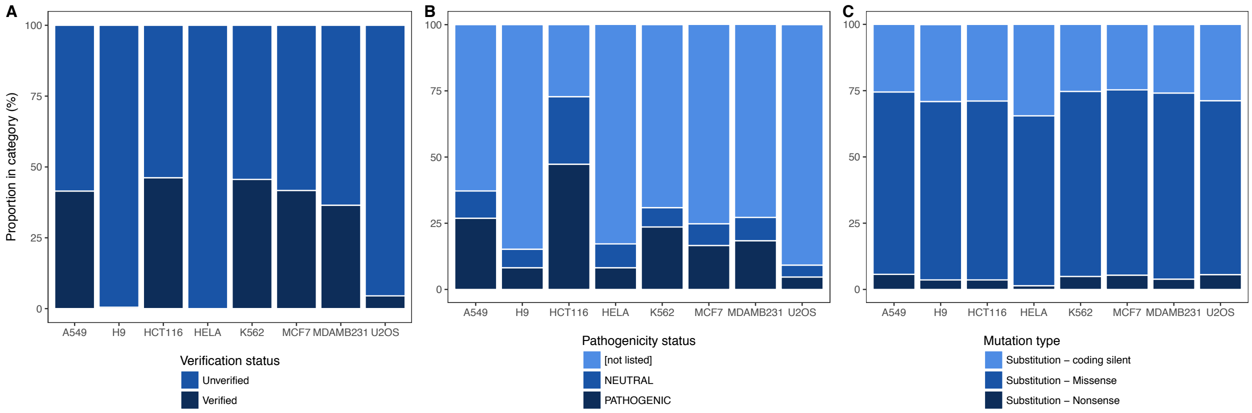

Supplementary table 3

Both the read length and the number of variants called for each dataset are confounding factors for the correlation analyses previously performed. Here we correlate the each parameter to the similarity score, to rule out that they contribute to the correlations between similarity score and DEG parameters.

```r
# Get same-cell, high quality datasets
data.transc <- read.table("sdata.4.transcriptome.stats.txt", header = TRUE,
                          sep = "\t")
data <- data.transc[data.transc$cell.1 == data.transc$cell.2, ]
data <- data[data$sample.1 != data$sample.2, ]
data <- data[c("sample.1", "sample.2", "calls", "score", "cell.1")]
err_datasets <- c("GSE81469", "GSE77033", "GSE68471", "GSE39872")
data <- data[!(data$sample.1 %in% err_datasets) &
             !(data$sample.2 %in% err_datasets), ]

# Get date metadata
metadata_full <- read.table("sdata.1.metadata.txt", sep = "\t", header = TRUE,
                            quote = "", fill = TRUE, stringsAsFactors = FALSE,
                            comment.char = "")
metadata_conf <- metadata_full[c("GSE", "LoadDate")]

# Convert dates to numeric for correlations
metadata_conf$LoadDate <- as.Date(metadata_conf$LoadDate,
                                  format = "%d-%m-%y")
metadata_conf$time <- as.numeric(as.POSIXct(metadata_conf$LoadDate,
                                            format = "%d-%m-%y",
                                            tz = "GMT"))

# Merge data and metadata
data <- merge(data, metadata_conf, by.x = "sample.1", by.y = "GSE",
              all.x = TRUE)
data <- merge(data, metadata_conf, by.x = "sample.2", by.y = "GSE",
              all.x = TRUE)
names(data) <- gsub("\\.y", ".2", gsub("\\.x", ".1", names(data)))
data <- data[!duplicated(data[c("sample.1", "sample.2")]), ]
data$delta_time <- abs(data$time.1 - data$time.2)

# Perform correlations
cells <- c("H9", "HCT116", "MCF7", "MDAMB231", "U2OS")
corrs <- data.frame()
for (cell in cells) {
    current <- data[data$cell.1 == cell, ]
    cor_calls <- cor.test(current$score, current$calls, method = "pearson")
    cor_date <- cor.test(current$score, current$delta_time, method = "pearson")
    corrs <- rbind(corrs, data.frame(cell = cell,
                                     cor_calls = round(cor_calls$estimate, 3),
                                     p_calls = round(cor_calls$p.value, 3),
                                     cor_date = round(cor_date$estimate, 3),
                                     p_date = round(cor_date$p.value, 3)))
}

# Finalise and format
results <- as.data.frame(t(corrs[, -1]))
```

```r
names(results) <- corrs$cell
results
```

```
##              H9 HCT116  MCF7 MDAMB231   U2OS
## cor_calls 0.132 -0.071 0.250    0.018  0.312
## p_calls   0.268  0.304 0.018    0.897  0.044
## cor_date  0.139 -0.139 0.138   -0.111 -0.052
## p_date    0.244  0.044 0.194    0.417  0.746
```

Session info

```
## R version 3.5.0 (2018-04-23)
## Platform: x86_64-apple-darwin15.6.0 (64-bit)
## Running under: macOS High Sierra 10.13.4
##
## Matrix products: default
## BLAS: /Library/Frameworks/R.framework/Versions/3.5/Resources/lib/libRblas.0.dylib
## LAPACK: /Library/Frameworks/R.framework/Versions/3.5/Resources/lib/libRlapack.dylib
##
## locale:
## [1] en_US.UTF-8/en_US.UTF-8/en_US.UTF-8/C/en_US.UTF-8/en_US.UTF-8
##
## attached base packages:
## [1] grid      stats     graphics  grDevices utils     datasets  methods
## [8] base
##
## other attached packages:
## [1] bindrcpp_0.2.2     tidyr_0.8.1        scales_0.5.0
## [4] reshape2_1.4.3     multcompView_0.1-7 gridExtra_2.3
## [7] ggplot2_2.2.1      dplyr_0.7.5
##
## loaded via a namespace (and not attached):
##  [1] Rcpp_0.12.17     pillar_1.2.2     compiler_3.5.0   plyr_1.8.4
##  [5] bindr_0.1.1      tools_3.5.0      digest_0.6.15    evaluate_0.10.1
##  [9] tibble_1.4.2     gtable_0.2.0     pkgconfig_2.0.1  rlang_0.2.0
## [13] cli_1.0.0        yaml_2.1.19      stringr_1.3.1    knitr_1.20
## [17] rprojroot_1.3-2  tidyselect_0.2.4 cowplot_0.9.2    glue_1.2.0
## [21] R6_2.2.2         rmarkdown_1.9    purrr_0.2.4      magrittr_1.5
## [25] backports_1.1.2  htmltools_0.3.6  assertthat_0.2.0 colorspace_1.3-2
## [29] labeling_0.3     utf8_1.1.3       stringi_1.2.2    lazyeval_0.2.1
## [33] munsell_0.4.3    crayon_1.3.4
```
